# CbtA toxin of *Escherichia coli* inhibits cell division and cell elongation via direct and independent interactions with FtsZ and MreB

**DOI:** 10.1371/journal.pgen.1007007

**Published:** 2017-09-20

**Authors:** Danielle M. Heller, Mrinalini Tavag, Ann Hochschild

**Affiliations:** Department of Microbiology and Immunobiology, Harvard Medical School, Boston, MA, United States of America; University of Geneva Medical School, SWITZERLAND

## Abstract

The toxin components of toxin-antitoxin modules, found in bacterial plasmids, phages, and chromosomes, typically target a single macromolecule to interfere with an essential cellular process. An apparent exception is the chromosomally encoded toxin component of the *E*. *coli* CbtA/CbeA toxin-antitoxin module, which can inhibit both cell division and cell elongation. A small protein of only 124 amino acids, CbtA, was previously proposed to interact with both FtsZ, a tubulin homolog that is essential for cell division, and MreB, an actin homolog that is essential for cell elongation. However, whether or not the toxic effects of CbtA are due to direct interactions with these predicted targets is not known. Here, we genetically separate the effects of CbtA on cell elongation and cell division, showing that CbtA interacts directly and independently with FtsZ and MreB. Using complementary genetic approaches, we identify the functionally relevant target surfaces on FtsZ and MreB, revealing that in both cases, CbtA binds to surfaces involved in essential cytoskeletal filament architecture. We show further that each interaction contributes independently to CbtA-mediated toxicity and that disruption of both interactions is required to alleviate the observed toxicity. Although several other protein modulators are known to target FtsZ, the CbtA-interacting surface we identify represents a novel inhibitory target. Our findings establish CbtA as a dual function toxin that inhibits both cell division and cell elongation via direct and independent interactions with FtsZ and MreB.

## Author summary

Bacterially encoded toxin-antitoxin systems, which consist of a small toxin protein that is co-produced with a neutralizing antitoxin, are a potential avenue for the identification of novel antibiotic targets. These toxins typically target essential cellular processes, causing growth arrest or cell death when unchecked by the antitoxin. Our study is focused on the CbtA toxin of *E*. *coli*, which was known to inhibit both bacterial cell division and also bacterial cell elongation (the process by which rod-shaped bacteria grow prior to cell division). We report that the effects of CbtA on cell division and cell elongation are genetically separable, and that they are due to direct and independent interactions with its targets FtsZ and MreB, essential cytoskeletal proteins that direct cell division and cell elongation, respectively. Our genetic analysis defines the functionally relevant target surfaces on FtsZ and MreB; in the case of FtsZ this surface represents a novel inhibitory target. As a dual-function toxin that independently targets two essential cytoskeletal elements, CbtA could guide the design of dual-function antibiotics whose ability to independently target more than one essential cellular process might impede the development of drug resistance, which is a growing public health threat.

## Introduction

In *E*. *coli*, as in most other bacteria, cell shape is defined by the peptidoglycan sacculus [1], which is built by the coordinated efforts of two major protein complexes, the cell elongation complex and the cell division complex (reviewed in [2–4]). The cell elongation complex directs the insertion of new cell wall material into the *E*. *coli* lateral sidewall, causing a newly divided rod cell to increase in length (while maintaining a constant width). Once the elongated cell has approximately doubled its mass, the division complex (or divisome) builds a new septal wall at mid-cell, forming two equivalently sized rod-shaped daughter cells [2,5,6]. Functional disruption of either of these two complexes in *E*. *coli* results in striking cell morphological alterations. Cells that fail to divide form long filaments [7], whereas cells that are blocked for cell elongation lose their rod-shape and become spherical [8,9].

Peptidoglycan insertion by the cell division and cell elongation complexes is directed by a dedicated bacterial cytoskeletal element. Cell division is governed by the broadly conserved bacterial tubulin homolog and GTPase, FtsZ. FtsZ polymerizes into dynamic filaments that coalesce into a ring structure (referred to as the Z ring) at mid-cell. Once properly assembled at mid-cell, this Z ring serves as a scaffold for a large set of essential and non-essential protein components, resulting in formation of the mature division complex, which constructs the new septum (reviewed in [7,10]). Cell elongation in the majority of rod-shaped bacteria is mediated by the actin-homolog and ATPase, MreB [11–14]. MreB polymerizes to form antiparallel double filaments [15] that are peripherally associated with the inner leaflet of the cytoplasmic membrane [16]. *In vivo* fluorescence imaging studies have shown that MreB forms dynamic filament patches that move circumferentially along the long axis of the cell, directing the lateral incorporation of cell wall material [17–19].

The polymerization, assembly, and dynamics of these bacterial cytoskeletal elements are dictated by their inherent biochemical properties and further influenced by diverse modulatory proteins. FtsZ assembly is controlled by a complex set of positive and negative “house-keeping” regulators that spatiotemporally coordinate Z ring formation with the cell cycle [7,20–25]. FtsZ is also the target of several inhibitors that block its assembly in response to specific environmental cues. For example, in response to cellular DNA damage, the SOS inhibitor SulA blocks FtsZ assembly by sequestering FtsZ monomers [26–28]. Several exogenous inhibitors of FtsZ function, including phage-encoded proteins and small molecule inhibitors, have also been described in recent years [29–31].

The best-characterized MreB inhibitor is the small molecule antibiotic A22, which binds within the nucleotide-binding pocket of MreB and blocks double filament formation [15]. However, relatively few protein modulators of MreB function have been described [32–37] and the physiological relevance of their effects is unknown.

Among proteins that can alter cell shape, the CbtA (formerly known as YeeV) protein of *E*. *coli* is unusual in being able to inhibit both cell division and cell elongation. Previously proposed to target both FtsZ and MreB [32], CbtA is the toxin component of the prophage-encoded CbtA/CbeA chromosomal toxin-antitoxin system found in *E*. *coli* and other closely related species [38].Toxin-antitoxin systems are genetic modules that encode a small, stable toxin protein and a labile, cotranscribed antitoxin (reviewed in [39–41]). Capable of causing growth arrest or cell death, the toxins typically target essential cellular processes. Toxin-antitoxin systems are abundant in prokaryotic genomes [42] and have been implicated in the bacterial stress response [40,43,44].

Overexpression of the *cbtA* toxin gene in *E*. *coli* was shown by Tan et al. [32] to result in a cell growth defect and a loss of rod shape. Over the course of several hours, cells induced for *cbtA* expression formed swollen lemon-shaped cells with distinct poles; with prolonged induction, these lemon-shaped cells eventually lysed [32]. This morphology is reminiscent of the change in cell shape induced by a simultaneous block of cell division and cell elongation pathways in *E*. *coli*–specifically by the combined inhibition of FtsZ (via overexpression of *sulA*) and MreB (with A22 treatment) [45]. Consistent with the striking lemon-like morphology seen with *cbtA* overexpression, Tan et al. detected interactions between the CbtA toxin and both FtsZ and MreB *in vivo* (by yeast two-hybrid) and *in vitro* (by pull-down assay) [32]. Nonetheless, whether or not these interactions are directly responsible for the effects of CbtA overproduction on cell shape and cell growth has not been established; in particular, it is not known if CbtA, a small protein of only 124 amino acids, interacts independently with FtsZ and MreB to mediate its effects on cell shape and whether its interaction with these or other proteins is responsible for its toxic effects. Moreover, in light of evidence that in *E*. *coli* cell division and septum formation depend on an interaction between FtsZ and MreB [46], CbtA might conceivably exert its effects by interacting directly with only one or the other of these cytoskeletal elements [32].

Here, we genetically dissect the reported interactions of CbtA with FtsZ and MreB. Our analysis indicates that these interactions are direct and independent. We show further that both of these interactions are functionally relevant, contributing independently to CbtA-mediated toxicity and cell-shape perturbations. Our findings thus establish CbtA as a bona fide dual inhibitor of bacterial cell elongation and cell division. Moreover, by identifying the surface of each cytoskeletal element that is bound by CbtA, our findings describe new inhibitory surfaces that can be targeted to block cytoskeletal function.

## Results

### CbtA overproduction is toxic and causes drastic cell shape changes

**Fig 1:**
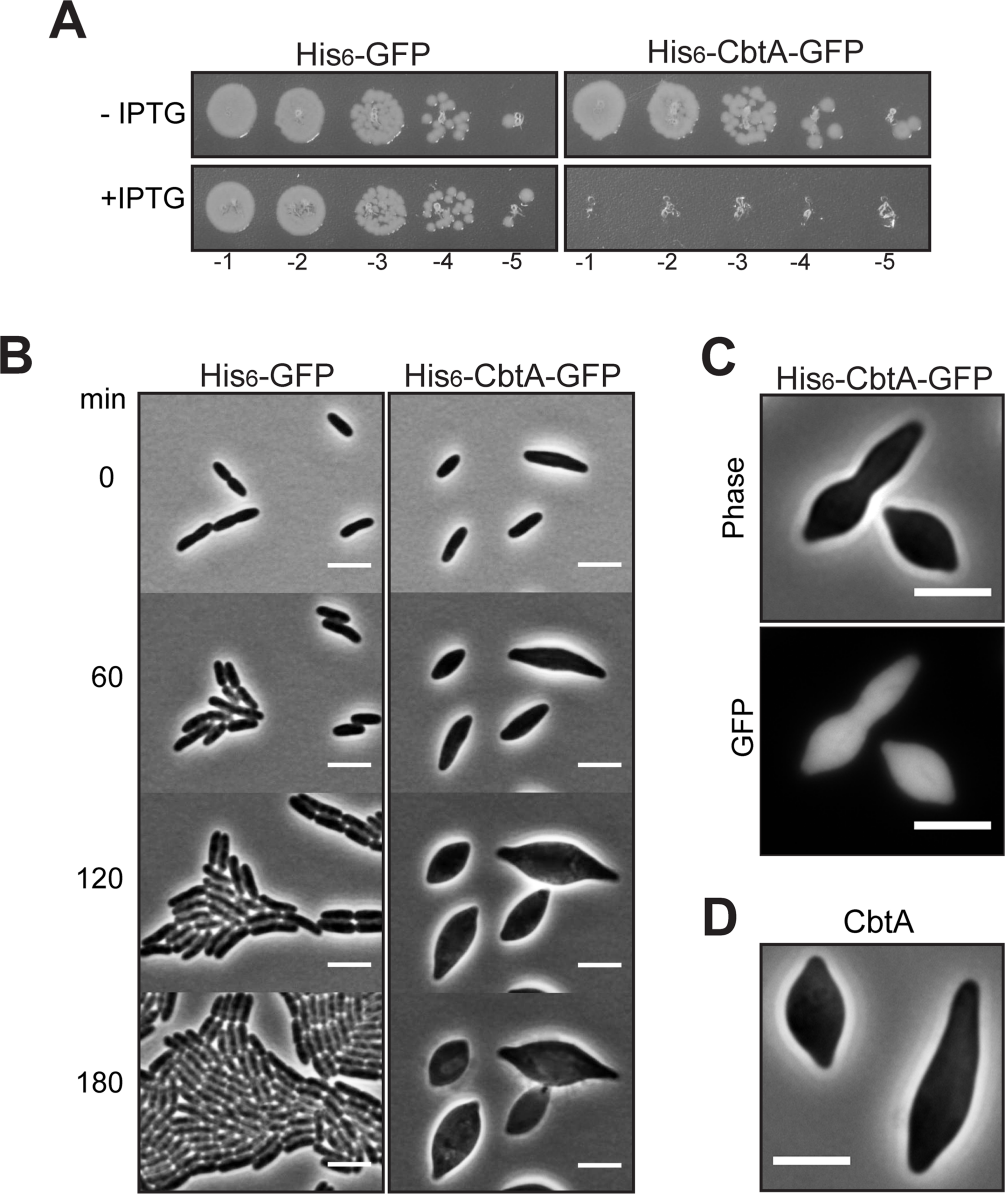
Overproduction of CbtA causes lemon-like morphology. (A) Spot dilution assay shows that IPTG-dependent production of His_6_-CbtA-GFP from *pT5-lac* on the multi-copy plasmid pMT139 causes a decrease in the viability of *E*. *coli* BW27785. The same strain producing His_6_-GFP from pMT136 is shown for comparison. For this experiment, all growth steps were done at 37°C. Late-log cultures were serially diluted and spotted on LB (Cm) with or without added IPTG (100 μM). Dilutions 10^−1^ to 10^−5^ are shown. (B) Cell morphology phenotypes. Cells of strains BW27785/pMT136 and BW27785/pMT139 were imaged every 3 min for 3 h at 30°C on 2% agarose pads containing LB and 100 μM IPTG. Images taken after 0, 60, 120, and 180 min are shown. (C) GFP fluorescence imaging of BW27785/ pMT139 induced with 100 μM IPTG for 2 h at 30°C shows that His_6_-CbtA-GFP is diffuse throughout the cell. (D) Overexpression of *cbtA* (untagged allele) from the multi-copy plasmid pDH325 in BW27785 also causes cells to adopt lemon-like morphology. Expression was induced with 200 μM IPTG for 2 h at 30°C. For all panels, scale bars represent 5 μm.

Consistent with previous reports, we observed that overproduction of CbtA (as a His_6_-CbtA-GFP fusion protein) under the control of a hybrid *T5-lac* promoter [47] in *E*. *coli* resulted in a severe decrease in viability (**Fig 1A**). Furthermore, time-lapse microscopy confirmed that upon overproduction of His_6_-CbtA-GFP, cells failed to divide, adopting a morphology resembling swollen lemons, and eventually lysed (**Fig 1B**). Observation of GFP fluorescence in these cells revealed that the His_6_-CbtA-GFP fusion protein was distributed diffusely throughout the entire bloated cell (**Fig 1C**); a high background of diffuse cytoplasmic fluorescence even early after the induction of fusion protein synthesis obscured any possible co-localization with cytoskeletal elements at earlier time points. Importantly, we found that overproduction of untagged CbtA yielded an identical lemon-shape phenotype (**Fig 1D**). As the various images in **Fig 1** illustrate, whereas essentially all the cells visualized manifested drastic morphological change, the individual lemon-shaped cells displayed striking heterogeneity. Many cells resembled smooth lemons, while others had pronounced tubular projections at one or both poles; bi-lobed lemon-shaped cells (such as the one shown in **Fig 1C**) were seen by time-lapse microscopy to form from pre-constricted cells (see **S1A Fig**). These morphologies are consistent with the varied cell shapes observed by Varma et al. upon combined FtsZ and MreB inhibition [45].

### Transcription-based bacterial two-hybrid assay detects interaction between CbtA and both FtsZ and MreB

**Fig 2:**
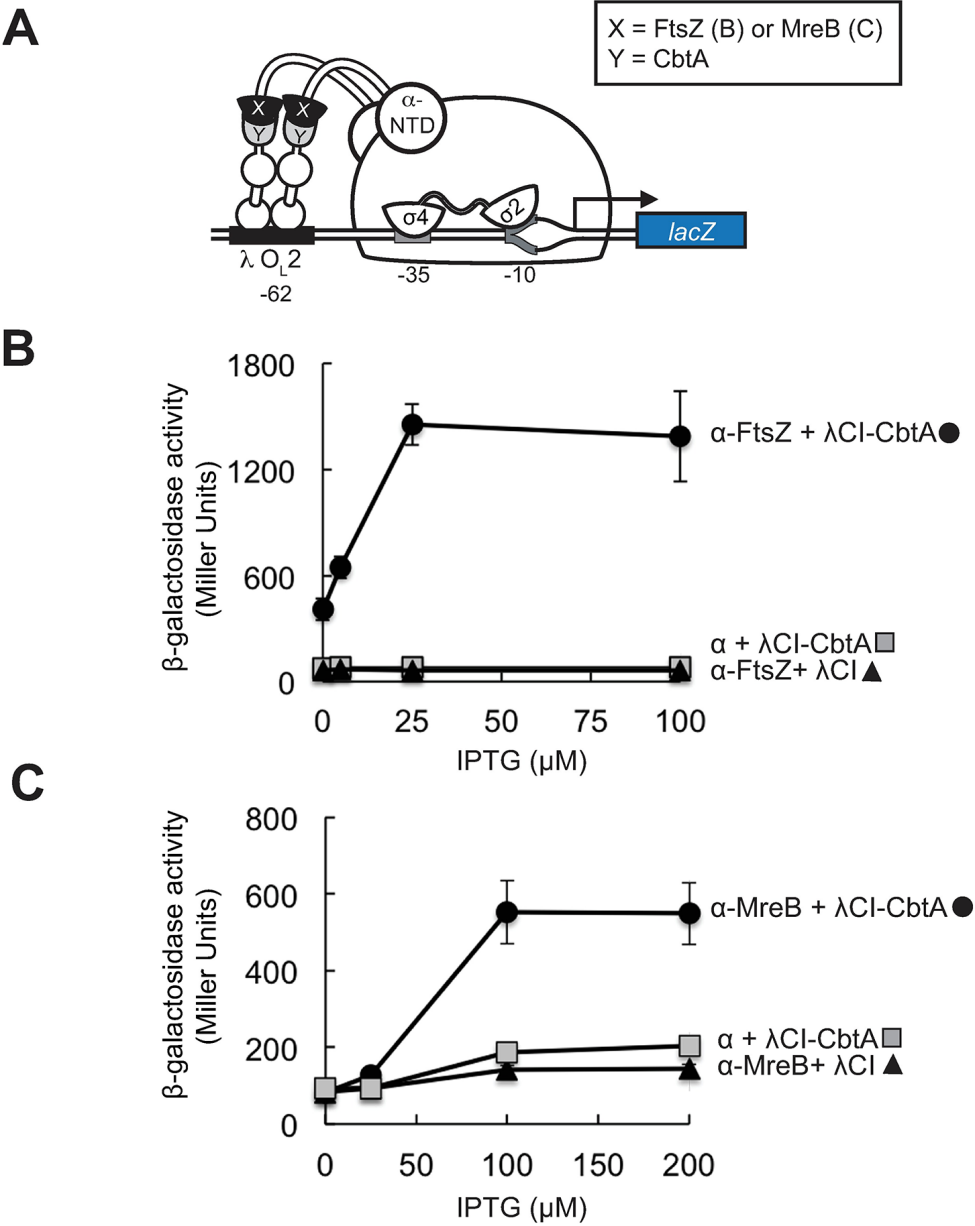
Detection of CbtA interaction with FtsZ and MreB in a transcription-based bacterial two-hybrid system. (A) Bacterial two-hybrid assay used to detect interactions of CbtA. Cartoon depicts how interaction between protein moieties X and Y, fused respectively to the N-terminal domain of the α subunit of *E*. *coli* RNA polymerase (α-NTD) and the λ CI protein (λCI), activates transcription from test promoter p*lac*O_L_2–62, which bears the λ operator O_L_2 centered 62 bp upstream from the *lac* core promoter. In reporter strain FW102 O_L_2–62, test promoter p*lac*O_L_2–62 is located on an F′ episome and drives the expression of a linked *lacZ* gene. (B, C) Results of β-galactosidase assays performed with FW102 O_L_2–62 cells that contained two compatible plasmids: one that encoded the λCI-CbtA fusion protein or λCI and another that encoded the indicated α fusion protein (α-FtsZ in (B) and α-MreB in (C)) or wild-type α. The plasmids directed the synthesis of the fusion proteins under the control of IPTG-inducible promoters, and the cell cultures were assayed at the indicated concentrations of IPTG (0, 5, 25 and 100 μM IPTG for (B); 0, 25, 100, and 200 μM IPTG for (C)). Each point represents the average of triplicate values; error bars represent standard deviation.

Tan et al. detected interaction between CbtA and its proposed cytoskeletal targets in a yeast two-hybrid system [32]. Similarly, we detected interaction between CbtA and both FtsZ and MreB in a bacterial two-hybrid system developed in our lab [48,49]. In this assay, contact between a protein domain (X) fused to the α subunit of *E*. *coli* RNA polymerase and a partner domain (Y) fused to the CI protein of bacteriophage λ (λCI) activates transcription of a *lacZ* reporter gene under the control of a test promoter bearing an upstream λCI-binding site (**Fig 2A**). In this case, we fused CbtA to λCI and either FtsZ or MreB to α. We detected an 18-fold increase in *lacZ* expression in the presence of the λCI-CbtA and α-FtsZ fusion proteins (**Fig 2B**) and a 3-fold increase in *lacZ* expression in the presence of the λCI-CbtA and α-MreB fusion proteins (**Fig 2C**). Whereas these results are consistent with the idea that CbtA can interact directly with both FtsZ and MreB, they do not exclude the possibility that chromosomally encoded FtsZ may serve as a protein bridge linking the fused CbtA and MreB moieties. Our genetic analysis below addresses this possibility.

### Identification of amino acid substitutions in CbtA that compromise its ability to interact with either FtsZ or MreB

**Fig 3:**
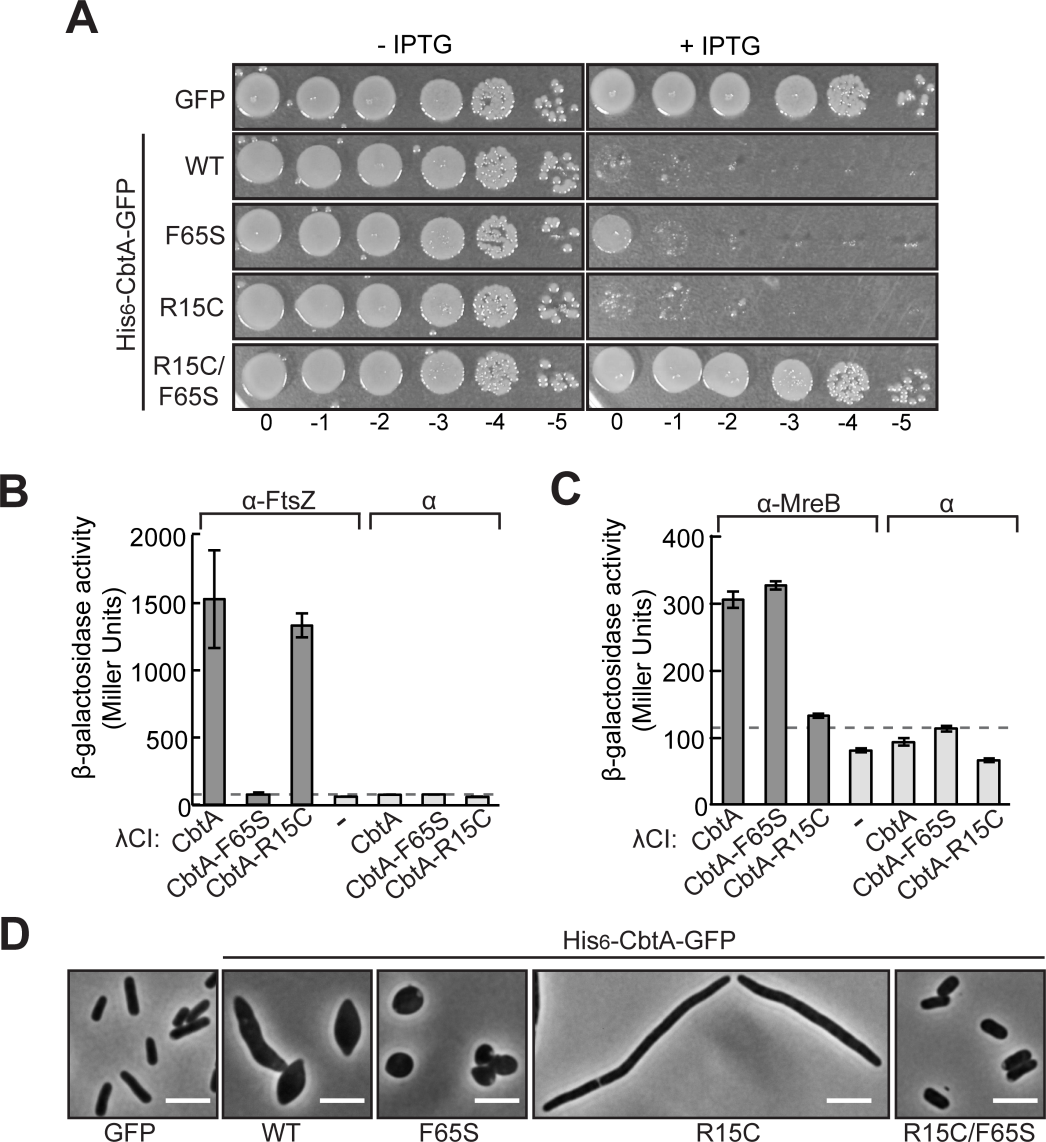
CbtA interactions with FtsZ and MreB are genetically separable. (A) BW27785 cells transformed with the relevant plasmids (pMT136, pMT139, pMT146, pDH253, and pDH262, from top to bottom) were grown in LB (Cm) at 30°C without induction until they reached mid to late-log phase. Cultures were normalized, serially diluted, and spotted on LB (Cm) with or without 50 μM IPTG. Plates were incubated overnight at 30°C. Dilutions 10^0^ to 10^−5^ are shown. (B, C) Results of β-galactosidase assays performed with two-hybrid reporter strain cells (see **Fig 2** legend) that contained two compatible plasmids: one that encoded the indicated λCI-CbtA fusion protein or λCI and another that encoded the indicated α fusion protein (α-FtsZ in (B) and α-MreB in (C)) or wild-type α. The cells were grown in the presence of 25 μM IPTG in (B) and 100 μM IPTG in (C). Bars represent averages of triplicate values and error bars represent standard deviation; dashed lines designate highest basal *lacZ* expression, i.e. the Miller Unit value of the highest empty vector control. (D) Cultures of the same transformed cells as described in (A) were imaged after 2 h induction with 50 μM IPTG at 30°C. Scale bars represent 5 μm.

To determine if CbtA can interact independently with FtsZ and MreB and to examine whether these interactions contribute directly to CbtA-mediated toxicity, we sought to identify mutations that specifically disrupt each of these interactions. We began by testing a CbtA variant that we had isolated on the basis of reduced toxicity (**S1 Text** and **S1 Fig**), which bore the substitution F65S. Although further analysis revealed that mutant CbtA-F65S was only slightly less toxic than wild-type CbtA when overproduced in *E*. *coli* (**Fig 3A**), bacterial two-hybrid analysis revealed that substitution F65S in the CbtA moiety of the λCI-CbtA fusion protein specifically disrupted its interaction with FtsZ (**Fig 3B**), without compromising its interaction with MreB (**Fig 3C**). Morphological observations were consistent with these results; upon overproduction of His_6_-CbtA-F65S-GFP to an intracellular level comparable to that of the wild-type protein (**S1C Fig**), cells adopted a sphere-like rather than a lemon-like morphology (**Fig 3D**). As seen by time-lapse microscopy (**S1A Fig**), over the course of a three-hour induction period, cells producing His_6_-CbtA-F65S-GFP lost their rod shape, becoming spheroidal. These spheroidal cells continued to divide for one to two generations, gradually increasing in diameter until they lysed, a phenotype that mirrors that observed upon depletion of MreB [8,9]. We conclude that CbtA’s ability to block cell division is due to a direct interaction with FtsZ. In addition, these findings suggest that CbtA-F65S is able to interact with MreB and mediate a block in cell elongation even in the absence of an interaction with FtsZ.

To further evaluate the proposition that CbtA’s ability to block cell elongation is due to a direct interaction with MreB, we sought to identify a CbtA variant with the opposite interaction profile: strong FtsZ interaction and abrogated MreB interaction. To do this, we used a two-hybrid-based screening strategy (see Materials and Methods). Specifically, we introduced random mutations into the gene fragment encoding the CbtA moiety of the λCI-CbtA fusion protein, transformed the mutagenized library into reporter strain cells containing the α-MreB fusion protein, and screened for clones with reduced expression of the *lacZ* reporter gene. λCI-CbtA mutants identified in this manner were then counter-screened to identify those that supported high levels of *lacZ* expression in the presence of the α-FtsZ fusion protein. Using this two-step screening procedure, we identified substitution R15C, which specifically disrupted the interaction of CbtA with MreB (**Fig 3C**), without compromising its interaction with FtsZ (**Fig 3B**). Consistent with these two-hybrid data, induction of His_6_-CbtA-R15C-GFP production was toxic and caused the cells to form filaments, rather than adopting the lemon shape observed when the wild-type protein was produced at comparable levels (**Fig 3A, Fig 3D, S1C Fig**). We conclude that CbtA’s ability to block cell elongation is due to a direct interaction with MreB, such that the CbtA-R15C variant, which still interacts with FtsZ, blocks cell division without blocking cell elongation. Together, the two-hybrid data and morphological phenotypes produced by the CbtA-F65S and CbtA-R15C variants demonstrate that the inhibitory functions of the CbtA toxin are independent and genetically separable.

### Compromising the ability of CbtA to interact with both FtsZ and MreB relieves cell toxicity

Although cells overproducing either His_6_-CbtA- F65S-GFP or His_6_-CbtA-R15C-GFP were still inviable (**Fig 3A**), we found that overproduction of the His_6_-CbtA-R15C/F65S-GFP double mutant, which accumulated to comparable levels as the wild-type protein (**S1C Fig**), did not influence viability (**Fig 3A**). Furthermore, cells producing this variant maintained their rod shape, exhibiting only minor morphological perturbations (**Fig 3D**). These findings establish the functional relevance of both the CbtA-FtsZ interaction and the CbtA-MreB interaction, each of which contributes to the lemon-like morphology and the viability defect observed upon CbtA overproduction.

As an additional readout of the physiological perturbations caused by each of our CbtA variants, Z ring formation was monitored in cells producing the untagged mutant proteins. We first used a strain that constitutively produces a ZapA-GFP fusion from its native locus [50]. Because ZapA-GFP forms fluorescent ring structures that require proper assembly of the FtsZ ring, this fusion protein can serve as a proxy for FtsZ localization [50,51]. When the ZapA-GFP strain was transformed with an empty vector, fluorescent ZapA-GFP bands were observed at mid-cell in the majority of cells (**S1D Fig).** In contrast, after two hours of *cbtA* expression, ZapA-GFP exhibited patchy, cloud-like localization throughout the resulting lemon-shaped cells, suggesting disruption of Z ring formation (**S1D Fig**). When ZapA-GFP localization was observed in cells producing CbtA-R15C, the majority of cell filaments did not contain visible ring structures, and ZapA-GFP again formed cloud-like structures (**S1D Fig**). In cells overproducing the less toxic CbtA-R15C/F65S variant, rod shape was maintained, and ZapA-GFP rings were observed in most cells (**S1D Fig**). Importantly, overproduction of the untagged CbtA variants resulted in identical morphologies to those observed with the His_6_/GFP constructs (compare **Fig 3D** phase contrast images with those shown **in S1D Fig**).

A recent study reported similar ZapA-GFP cloud-like structures under conditions where FtsZ assembly and localization were disrupted, indicating that ZapA is able to localize in an FtsZ-independent manner [52]. To determine whether the patchy ZapA-GFP localization we saw upon overproduction of CbtA and CbtA-R15C was similarly occurring in an FtsZ-independent manner, we examined the localization of GFP-FtsZ (overproduced in a strain also containing wild-type endogenous *ftsZ*) in the presence of our untagged CbtA variants. Consistent with our ZapA-GFP data, we saw that after two hours of induction of CbtA or CbtA-R15C production, the majority of cells did not contain Z rings; however, unlike ZapA-GFP, GFP-FtsZ exhibited diffuse localization throughout the cell with no cloud-like structures observed (**S1E Fig**). Thus, it seems likely that the ZapA-GFP patches seen in **S1D Fig** are forming independently of FtsZ. GFP-FtsZ was found to localize to mid-cell ring structures in cells transformed with an empty vector and in cells producing the CbtA-F65S/R15C double mutant variant (**S1E Fig**). Taken together, the ZapA-GFP and GFP-FtsZ localization patterns suggest that wild-type CbtA and CbtA-R15C are able to disrupt FtsZ assembly and localization, blocking cell division, whereas the double mutant variant does not. The results of these analyses are consistent with the genetic evidence indicating that CbtA inhibits cell division and cell elongation via independent and separable interactions.

### The CTT of FtsZ does not mediate interaction with CbtA

We next sought to identify the CbtA interaction sites on FtsZ and MreB required for CbtA to mediate its inhibitory effects on cell division and cell elongation. Tan et al. reported that removal of the last 66 residues of FtsZ eliminated the yeast two-hybrid interaction detected between CbtA and FtsZ as well as the interaction between MreB and FtsZ [32]. The last 66 residues of *E*. *coli* FtsZ include the conserved 15 amino acid C-terminal tail domain (CTT), which serves as a site of interaction for a variety of protein factors that regulate FtsZ assembly [53–62], raising the possibility that CbtA too binds the CTT. We sought to test this possibility using our bacterial two-hybrid assay.

**Fig 4:**
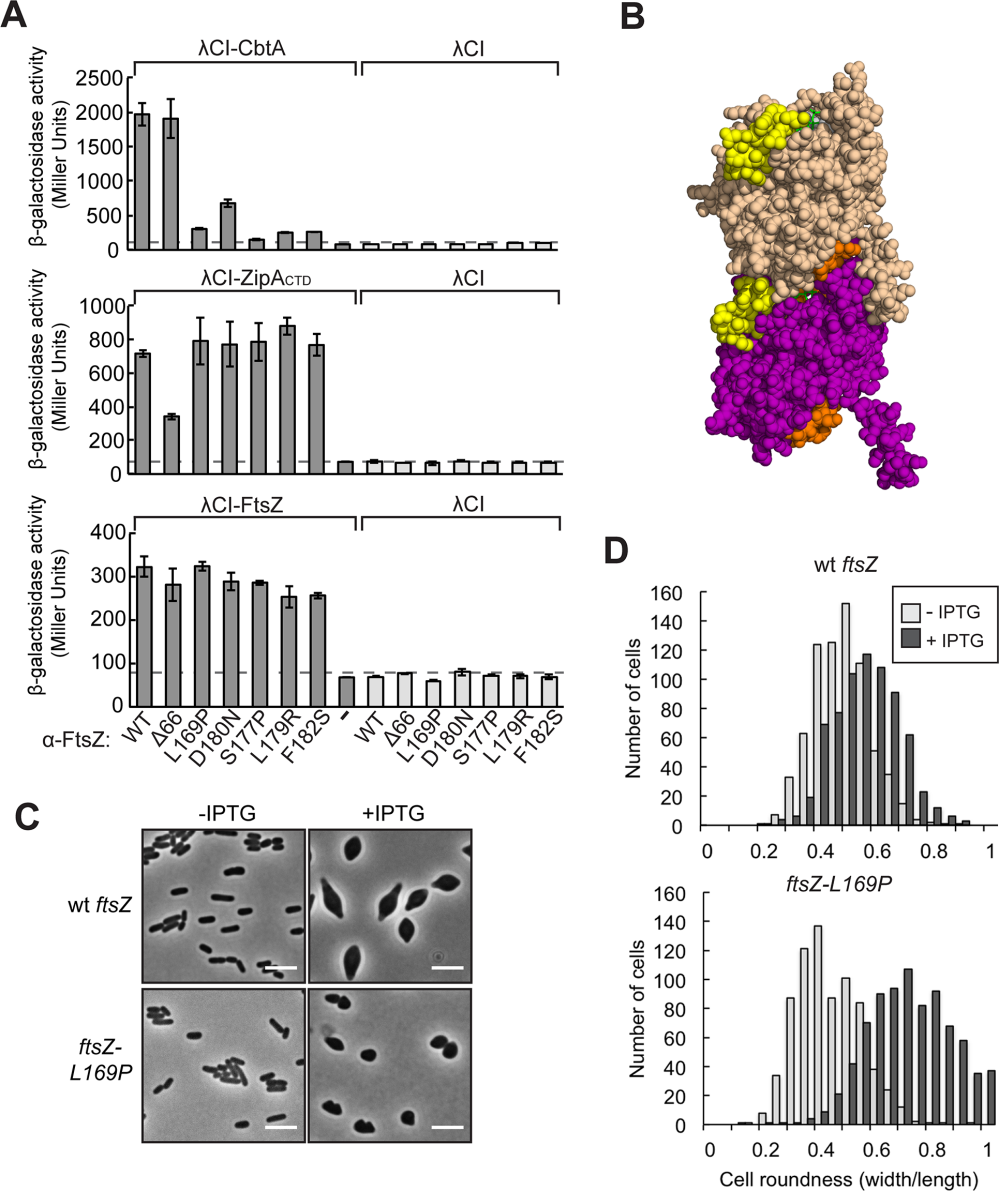
Residues in the H6/H7 loop are necessary for CbtA-FtsZ interaction. (A) Graphs show the results of β-galactosidase assays performed with two-hybrid reporter strain cells containing two compatible plasmids: one that encoded the indicated α-FtsZ variant or wild-type α (all three panels) and another that encoded the indicated λCI fusion protein (λCI-CbtA, top; λCI-ZipA_CTD_ (residues 186–328), middle; λCI-FtsZ, bottom) or λCI. The cells were grown in the presence of 100 μM IPTG (top), 25 μM IPTG (middle), and 100 μM IPTG (bottom). Bars represent the average β-galactosidase activity from three independent measurements, and error bars represent standard deviations. Dashed lines designate highest basal *lacZ* expression, i.e. the Miller Unit value of the highest empty vector control. (B) Crystal structure depicting two *Methanococcus jannaschii* FtsZ monomers (space-filled in wheat and purple, PDB 1W5B [64]) in head-to-tail arrangement. The T7 loop (shown in orange) contacts the GTP nucleotide (green) bound within the GTP-binding pocket. The loop connecting helix 6 and helix 7 (H6/H7 loop) is shown in yellow. (C) Phase contrast images were taken of either wild-type BW27785 or BW27785 *ftsZ-L169P* (DH73) cells overproducing (or not) His_6_-CbtA-GFP (encoded on plasmid pMT139). Overnight cultures grown at 30°C in M9 maltose (0.4% maltose, 1mM MgSO_4_, 0.01% casamino acids) were back-diluted 1:3,000 into fresh medium and grown at 30°C for ~14 h until they reached an OD600 of 0.3. Cultures were then induced with 100 μM IPTG (or not) and grown for 8 h at 30°C. Scale bars represent 5 μm. (D) Quantification of cell roundness (cell width/ cell length) from cells in (C). Histograms include compiled measurements from three-independent experiments (*n* = 725, wild-type without IPTG; *n* = 706, wild-type + IPTG; *n* = 739, L169P without IPTG; *n* = 813, L169P +IPTG). Width and length measurements were made manually in ImageJ[65] using the ObjectJ plugin.

As a positive control, we first tested the ability of the FtsZ membrane-anchoring protein ZipA (its cytoplasmic C-terminal domain) to interact with FtsZ. Structural data indicate that the C-terminal domain (CTD) of ZipA (ZipA_CTD_) binds the FtsZ-CTT [57]. We detected a strong interaction (resulting in a 10-fold increase in *lacZ* expression) between FtsZ and the ZipA_CTD_, and this interaction was compromised by removal of the C-terminal 66 residues of the FtsZ moiety (**Fig 4A**), consistent with the structural data [57] and previously reported yeast two-hybrid analysis [63]. However, surprisingly, we found that FtsZ-Δ66 maintained an interaction with CbtA comparable to that of the wild-type protein (**Fig 4A**), suggesting that the FtsZ-CTT is not necessary for the CbtA-FtsZ interaction. We were unable to detect an interaction between wild-type FtsZ and *E*. *coli* MreB in our two-hybrid system (**S2A Fig**) and thus could not determine whether or not this C-terminal truncation had any effect on that reported interaction.

### Substitutions in the H6/H7 loop of FtsZ disrupt the CbtA-FtsZ interaction and prevent CbtA from inhibiting cell division

Because, in our bacterial two-hybrid system, the last 66 residues of FtsZ did not appear to mediate the interaction with CbtA, we sought to identify substitutions in FtsZ that specifically disrupt its interaction with CbtA. To do this, we introduced random mutations into the gene fragment encoding the FtsZ moiety of the α-FtsZ fusion protein, introduced the mutagenized library into reporter strain cells containing the λCI-CbtA fusion protein and screened for colonies with reduced *lacZ* expression on appropriate indicator medium (see Materials and Methods). To identify FtsZ mutants specifically deficient for interaction with CbtA as opposed to generally destabilized variants, we performed a counter-screen based on the ability of FtsZ to interact with itself. Specifically, we detected a 4-fold increase in *lacZ* expression in reporter strain cells containing both the α-FtsZ fusion protein and a λCI-FtsZ fusion protein (**Fig 4A**); a similar interaction was previously reported in the context of both the yeast two-hybrid system [58] and an alternative bacterial two-hybrid system [46]. Thus, we screened for amino acid substitutions in the FtsZ moiety of the α-FtsZ fusion protein that reduced *lacZ* reporter gene expression in cells containing the λCI-CbtA fusion protein, but not in cells containing the λCI-FtsZ fusion protein. Among those amino acid substitutions that reduced *lacZ* expression by at least 60% in cells containing λCI-CbtA and by less than 25% in cells containing λCI-FtsZ, all localized to a small region encompassing residues 169–182 (**Fig 4A**). These amino acid substitutions did not compromise the interaction between FtsZ and the ZipA_CTD_ (**Fig 4A**).

FtsZ residues 168–182 make up a loop region connecting α-helices 6 and 7 (the H6/H7 loop) in the GTP-binding N-terminal domain of FtsZ. FtsZ protofilament crystal structures from several bacterial species show that the H6/H7 loop (shown in yellow in **Fig 4B**) lies at the longitudinal interface formed by stacked FtsZ monomers [64,66,67].

To evaluate whether or not the H6/H7 loop residues identified in our genetic screen are functionally important for the CbtA-FtsZ interaction, we sought to test the effect of CbtA overproduction in an *E*. *coli* strain bearing one of the H6/H7 loop mutations at the endogenous *ftsZ* locus. We found that the *ftsZ-L169P* allele was able to support growth when introduced into the chromosomal *ftsZ* locus (**S2 Fig**). Although this strain did not fully support cell division in fast-growth conditions (LB at 37°C)–we saw a subset of filamented cells and notable heterogeneity in cell length (**S2C Fig**)–this division defect was partially rescued by slower growth in LB at 30°C and fully rescued by growth in M9 minimal medium at 30°C (**S2C Fig**). Indeed, in minimal medium, we observed comparable cell lengths for the wild-type and *ftsZ-L169P* strains (**S2D Fig**). Western blot analysis with an FtsZ-recognizing antibody indicated that the FtsZ-L169P mutant protein accumulated within cells to levels comparable to that of the wild-type protein (**S2E Fig**).

We were therefore in a position to test whether or not the FtsZ L169P substitution specifically blocked the ability of CbtA to inhibit cell division. We found that when wild-type His_6_-CbtA-GFP was overproduced in the *ftsZ-L169P* strain, in M9 maltose at 30°C, cells lost their rod shape, but formed small spherical or sphere-like cells rather than lemon-shaped cells (**Fig 4C**), the expected phenotype for a defect in cell elongation. In particular, previous studies have shown that growth in minimal medium at low temperature can suppress the lethality of a cell elongation defect, such that the cells do not form large spheres and lyse, but instead are able to propagate as small spheres [9]. Overproduction of His_6_-CbtA-GFP in the wild-type background in these same growth conditions resulted in the formation of lemon-shaped cells, as expected (**Fig 4C**). We quantified these morphological observations by measuring cell roundness (width divided by length), confirming that His_6_-CbtA-GFP overproduction in the *ftsZ-L169P* strain caused a more pronounced increase in roundness than in the wild-type strain (**Fig 4D**). These findings are consistent with the two-hybrid data and provide strong support for the idea that H6/H7 loop region is functionally implicated in the CbtA-FtsZ interaction.

**Fig 5:**
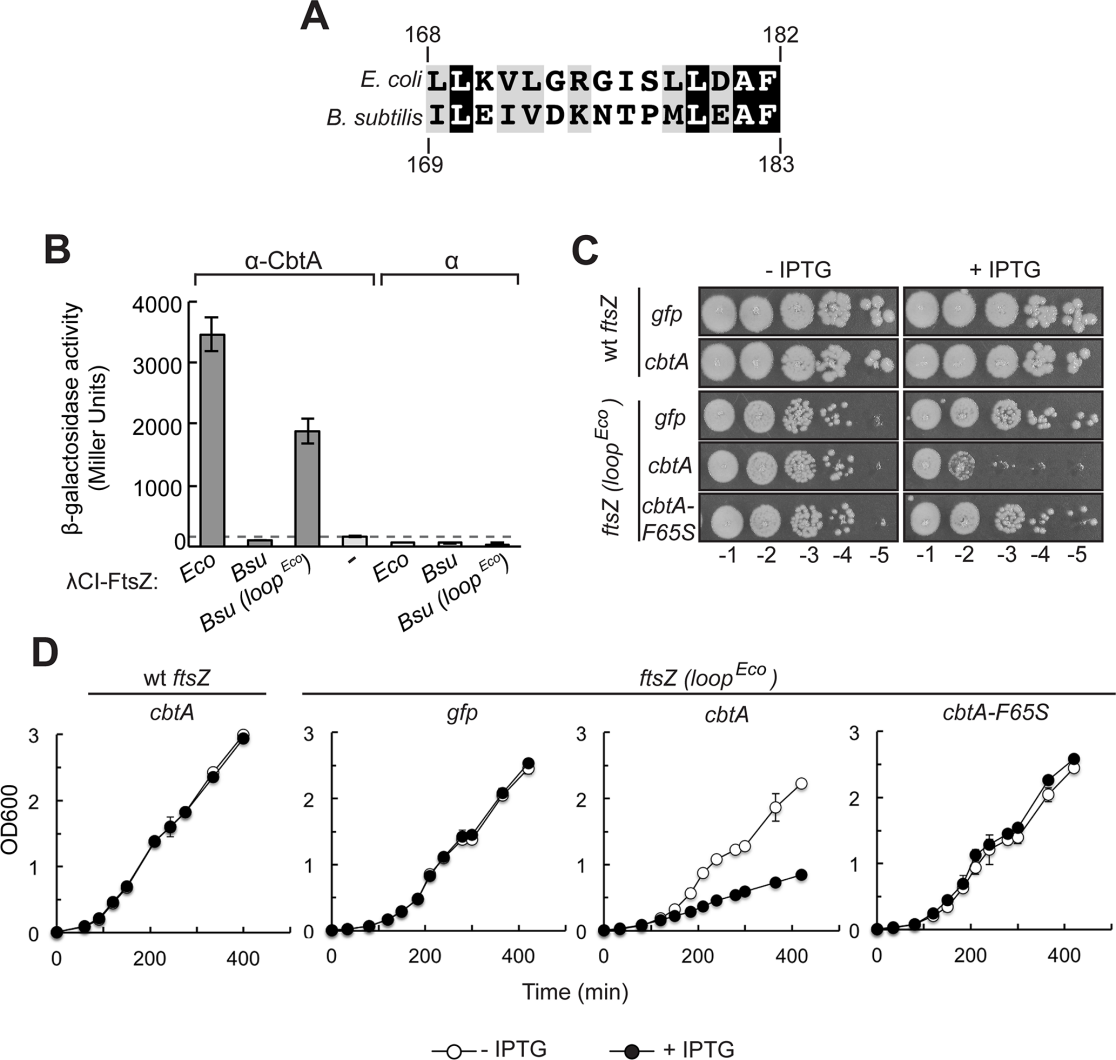
CbtA interacts with *Bsu* FtsZ chimera containing *Eco* H6/H7 loop sequence. (A) Amino acid sequence alignment of the H6/H7 loop sequences from *E*. *coli* (residues 168–182) and *B*. *subtilis* (residues 169–183) is shown. Identical residues are shown in black; similar residues are shown in gray. Alignment was prepared using Boxshade. (B) Two-hybrid analysis shows that a *Bsu* FtsZ chimera containing the H6/H7 loop sequence from *Eco* FtsZ (residues 169–183 of *Bsu* FtsZ are replaced with residues 168–182 of *Eco* FtsZ) can interact with CbtA. Reporter strain cells containing compatible plasmids encoding the indicated λCI-FtsZ variant (or λCI) and α-CbtA (or wild-type α) were grown in the presence of 100 μM IPTG and assayed for β-galactosidase. Bars represent the average β-galactosidase activity from three independent measurements; error bars represent standard deviations. Dashed line designates highest basal *lacZ* expression, i.e. the Miller Unit value of the highest empty vector control. (C) Spot dilution assay was used to measure CbtA toxicity in *B*. *subtilis* strains containing the indicated *ftsZ* allele and the indicated *his*_*6*_*-cbtA-gfp* allele (or *his*_*6*_-*gfp* only). A single colony of each strain was grown in LB at 37°C until late-log phase. All cultures were normalized to the same OD600 value, serially diluted, and spotted on LB plates with or without 1mM IPTG. Plates were incubated at 37°C overnight. Dilutions 10^−1^ to 10^−5^ are shown. (D) Growth curve analysis was performed on *B*. *subtilis* strains containing the indicated *ftsZ* allele and the indicated *his*_*6*_*-cbtA-gfp* allele (or *his*_*6*_-*gfp* only). Triplicate cultures of each strain were grown in LB ± 1 mM IPTG at 37°C over several hours. Each point represents the average of triplicate values; error bars represent standard deviation.

As a complementary approach, we developed a *Bacillus subtilis* heterologous system with which to evaluate the importance of residues in the FtsZ H6/H7 loop in enabling CbtA to interact functionally with FtsZ to inhibit cell division. Although the *E*. *coli* and *B*. *subtilis* FtsZ proteins share ~47% amino acid identity, comparison of the H6/H7 loop sequences reveals several non-conservative amino acid differences (**Fig 5A**). Thus, we surmised that if the H6/H7 loop mediates the interaction of CbtA with FtsZ, then the CbtA toxin should not interact with *B*. *subtilis* (*Bsu*) FtsZ. As shown in **Fig 5B**, CbtA was unable to interact with *Bsu* FtsZ by two-hybrid analysis; however, replacement of the *B*. *subtilis* H6/H7 loop with the *E*. *coli* loop (*Bsu ftsZ (loop*^*Eco*^*)*) resulted in a strong interaction between *Bsu* FtsZ and CbtA.

To test whether or not CbtA could inhibit cell division in *B*. *subtilis* cells containing either wild-type FtsZ or an FtsZ chimera bearing the *E*. *coli* H6/H7 loop region, we constructed strains with either the wild-type or chimeric *ftsZ (loop*^*Eco*^*)* (linked to *spec*) at the endogenous locus. These strains additionally harbored *gfp*, wild-type *cbtA* or *cbtA-F65S* (both alleles encode an N-terminal His_6_ tag preceding *cbtA* followed by a C-terminal GFP moiety) at the *ycgO* locus under the control of a strong inducible promoter (*pHYPERSPANK*). Overproduction of wild-type CbtA in the strain bearing the wild-type *ftsZ* allele had no effect on cell growth (**Fig 5C and 5D**) or any detectable effect on cell division (**S3A Fig**), consistent with our inability to detect an interaction between CbtA and *Bsu* FtsZ by two-hybrid analysis. The chimeric *ftsZ (loop*^*Eco*^*)* allele itself caused a slight growth defect manifest as decreased colony size (**Fig 5C)** and decreased growth rate in liquid medium (**Fig 5D)**; in addition, microscopic analysis of cells containing the chimeric *ftsZ (loop*^*Eco*^*)* allele revealed a cell division defect (**S3B and S3C Fig**). However, CbtA overproduction in this strain caused a severe growth defect both on plates (**Fig 5C**) and in liquid (**Fig 5D**) and resulted in increased cell lysis (**S3C Fig**), but overproduction of CbtA-F65S to comparable levels (**S3D Fig**) did not (**Fig 5C and 5D and S3C Fig**). We conclude that residues in the H6/H7 loop region of FtsZ dictate whether or not CbtA can interact functionally with FtsZ.

### CbtA interacts directly with the H6/H7 loop

**Fig 6:**
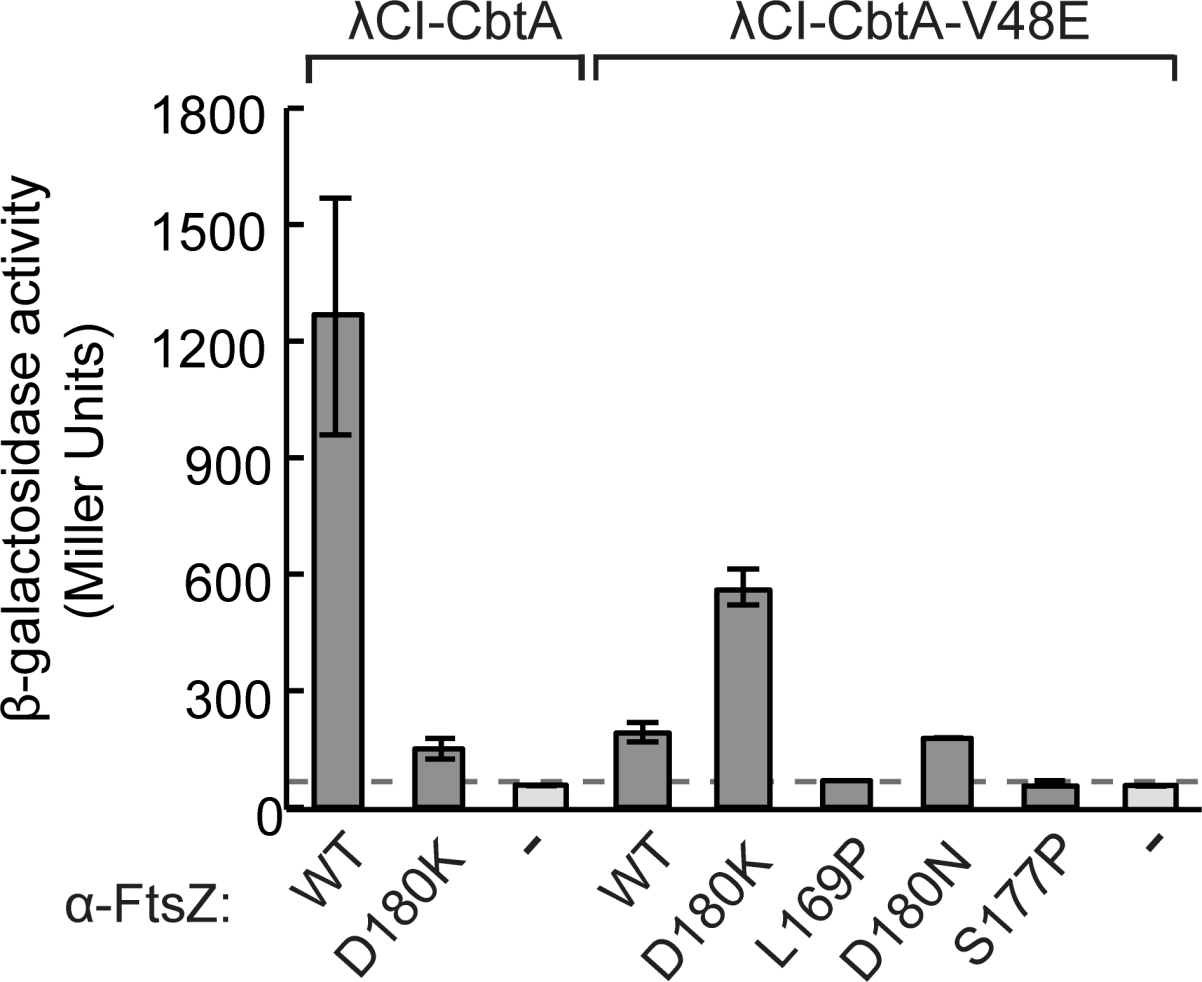
V48E substitution restores CbtA interaction with FtsZ loop mutant in allele-specific manner. Two-hybrid analysis shows that the CbtA-V48E substitution partially restores the CbtA-FtsZ (D180K) interaction, but disrupts the interaction between CbtA and wild-type FtsZ. Reporter strain cells containing compatible plasmids encoding the indicated λCI-CbtA variant and the indicated α-FtsZ variant (or wild-type α) were grown in the presence of 25 μM IPTG and assayed for β-galactosidase. Bars represent the average β-galactosidase activity from three independent measurements; error bars represent standard deviations. Dashed line designates the Miller Unit value of the highest empty vector control.

We next sought to determine whether CbtA makes direct contact with the H6/H7 loop of FtsZ. To do this, we aimed to identify compensatory substitutions in CbtA that restored its interaction with specific FtsZ H6/H7 loop mutants, using our two-hybrid system to screen for such mutant-suppressor pairs. To facilitate this analysis, we first sought to identify a charge reversal substitution in the H6/H7 loop that disrupted the CbtA-FtsZ interaction. Having identified substitution D180N in our original screen for disruptive mutations, we tested the effect of a charge reversal substitution at the same position (D180K). We found that this charge reversal substitution almost completely eliminated the two-hybrid interaction between FtsZ and CbtA (**Fig 6**), making it a suitable starting point for seeking to identify compensatory substitutions in CbtA. Accordingly, we transformed reporter strain cells containing the α-FtsZ-D180K fusion protein with a mutagenized library of plasmids encoding the λCI-CbtA fusion protein (bearing random mutations in the *cbtA* moiety) and screened for clones with elevated expression of the *lacZ* reporter gene. These candidate suppressor mutants were then pooled and counter-screened to identify those that maintained a low level of *lacZ* expression in the presence of the wild-type α-FtsZ fusion protein, and thus to identify substitutions that enabled CbtA to interact with FtsZ-D180K but not wild-type FtsZ. With this two-step screening procedure, we identified CbtA substitution V48E, which partially restored the interaction between CbtA and FtsZ-D180K (resulting in a 9-fold increase in *lacZ* expression) (**Fig 6**). The effect of this substitution was allele-specific, as CbtA-V48E was unable to interact with wild-type FtsZ or other H6/H7 loop mutants identified in our original screen (L169P, S177P, D180N) (**Fig 6**). We conclude that CbtA interacts directly with the H6/H7 loop of FtsZ.

### Substitutions on the flat side of the MreB monomer alter interaction with CbtA

**Fig 7:**
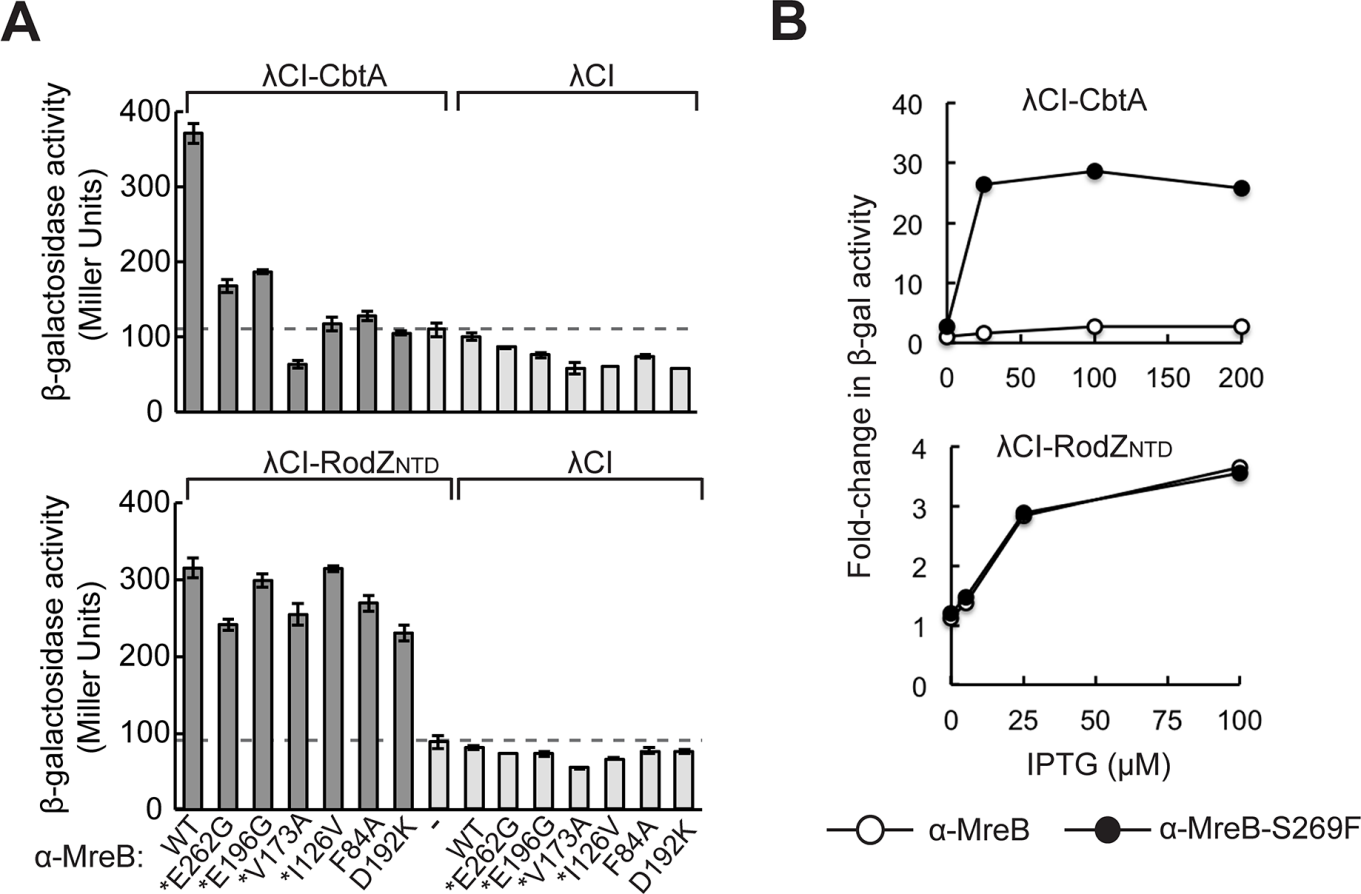
Substitutions at the MreB double filament interface alter CbtA interaction. (A) Graphs show the results of β-galactosidase assays performed with two-hybrid reporter strain cells containing two compatible plasmids: one that encoded the indicated α-MreB variant or wild-type α (both panels) and another that encoded the indicated λCI fusion protein (λCI-CbtA, top; λCI- RodZ_NTD_ (residues 2–84), bottom) or λCI. The cells were grown in the presence of 100 μM IPTG (top) or 25 μM IPTG (bottom). Bars represent the average Miller Unit values of biological triplicates; error bars represent standard deviation. Dashed lines designate highest basal *lacZ* expression, i.e. the Miller Unit value of the highest empty vector control. * denotes variants identified in our original two-hybrid screen. (B) To compare the two-hybrid interactions of α-MreB and α-MreB-S269F with λCI-CbtA (top) and λCI-RodZ_NTD_ (bottom), fold-change values are shown for cells grown in the presence of increasing concentrations of IPTG (top: 0, 25, 100, and 200 μM IPTG; bottom: 0, 5, 25, 100 μM IPTG). These fold-change values were calculated by dividing the average Miller Unit value of the strain producing both fusion proteins of interest (e.g. α-MreB and λCI-CbtA) by the average Miller Unit value of the relevant empty vector control with the highest β-galactosidase activity (e.g. α + λCI-CbtA). Average Miller Unit values were calculated from biological triplicates from a single representative experiment. Similar results were obtained from multiple independent experiments.

**Fig 8:**
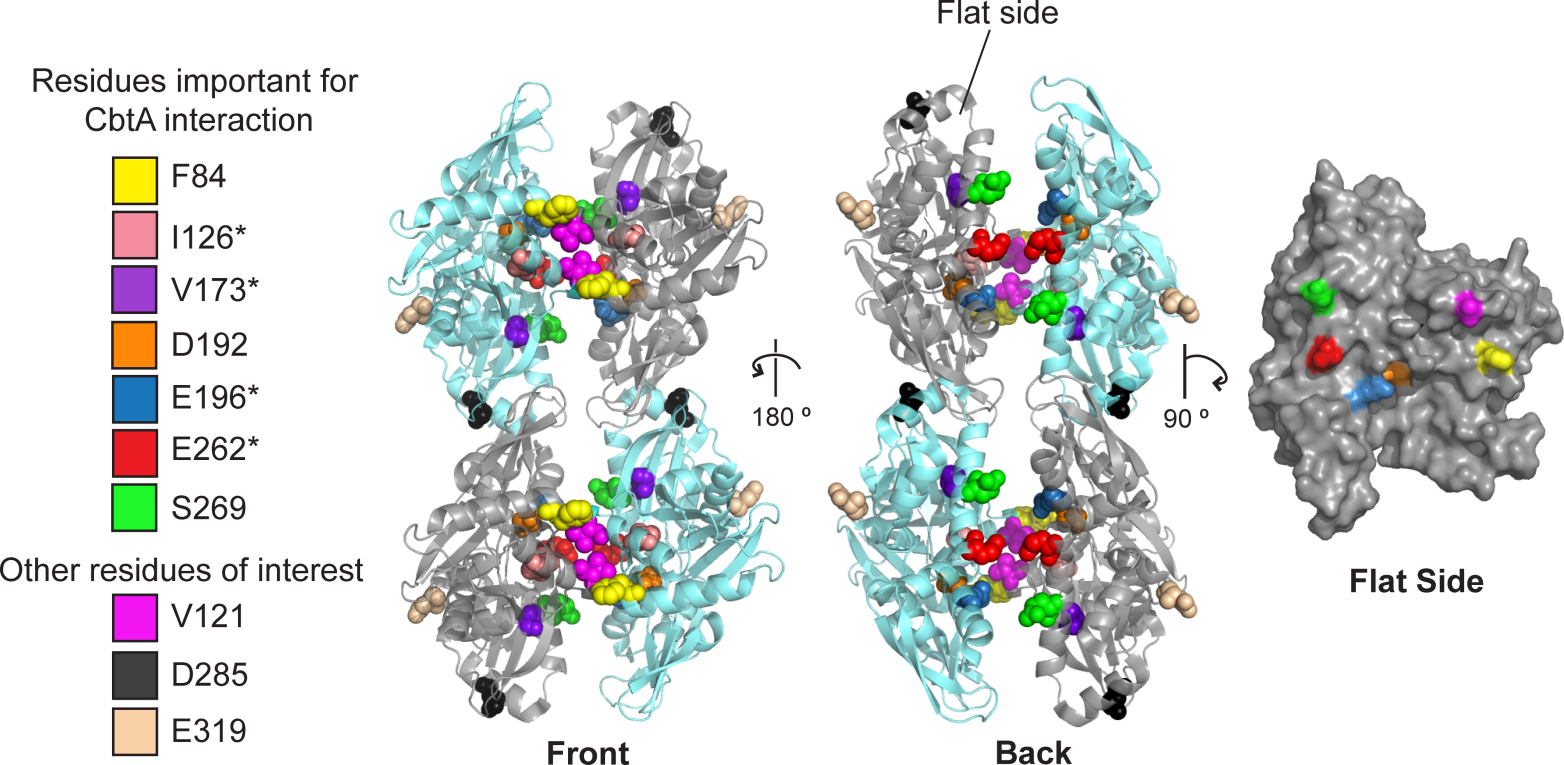
Substitutions that alter CbtA binding cluster at the inter-protofilament interface of MreB. Residues found to influence the CbtA-MreB two-hybrid interaction are mapped onto the *C*. *crescentus* MreB double filament interface (PDB 4cze [15]). Front and back views of the ribbon structure (left and middle, respectively) and the surface rendered model of a single subunit rotated 90° such that the flat surface is facing forward are shown. Residues are color coded as shown in the key on the left; *E*. *coli* residue designations are shown. Although the V121E substitution did not affect the ability of MreB to interact with CbtA via two-hybrid analysis, the corresponding *C*. *crescentus* MreB residue (V118), is shown in magenta to highlight the position of the main dimerization helix (helix 3) shown in [15] to be critical for double filament formation. Residue D285, which was found to be important for the interaction between MreB and FtsZ in *E*. *coli*, is shown in black [46]. Residue E319 is shown in wheat to illustrate the MreB surface bound by RodZ [69]. * denotes variants identified in our original two-hybrid screen.

Next, in an attempt to identify the MreB surface targeted by CbtA, we sought to isolate MreB variants reduced for their interaction with CbtA in our bacterial two-hybrid system. Specifically, we introduced random mutations into the gene fragment encoding the MreB moiety of the α-MreB fusion protein, introduced the mutagenized library into reporter strain cells containing the λCI-CbtA fusion protein and screened for colonies with reduced *lacZ* expression. To identify MreB mutants that were specifically deficient for CbtA interaction, each candidate was counter-screened for interaction with the cytoplasmic N-terminal domain of RodZ (RodZ_NTD_). RodZ is a component of the cell elongation complex, and its interaction with MreB has been established by bacterial two-hybrid and structural studies [68,69]. Using our two-hybrid system, we detected a 3 to 4-fold increase in *lacZ* expression in reporter strain cells containing both the α-MreB fusion protein and a λCI-RodZ_NTD_ (residues 2–84) fusion protein (**Fig 7A and 7B**). We confirmed the biological relevance of this interaction with the introduction of a charge reversal substitution (E319K in *E*. *coli* MreB, corresponding to E318K in *C*. *crescentus* MreB, shown in wheat in **Fig 8**) at the previously defined MreB-RodZ interface [69] (**S4A Fig**).

Using this two-step screening procedure, we identified four amino acid substitutions in MreB (I126V, V173A, E196G, and E262G) that reduced *lacZ* expression substantially in cells containing λCI-CbtA, but by less than 30% in cells containing λCI-RodZ_NTD_ (**Fig 7A**). We mapped these residues onto the *C*. *crescentus* (*Cc*) MreB double filament structure recently determined by the Lowe group [15] and found that they clustered at or near the interface formed by the paired protofilaments (**Fig 8**). This interface is formed by an interaction between the flat sides of the protofilaments, which pair in an antiparallel fashion. Stabilizing this antiparallel arrangement is an interaction between juxtaposed alpha helices (α-helix 3 in subdomain IA from one MreB subunit stacks onto α-helix 3 from the opposed subunit, with residue V121 (corresponding to *Cc* residue V118) playing a particularly important role; **Fig 8**). Two of the residues identified in our screen (E196 and E262, corresponding to *Cc* residues E193 and E261, respectively) are surface exposed on the flat side of the *Cc* MreB protofilament at the inter-protofilament interface. Although the other two residues (I126 and V173, corresponding to *Cc* I123 and *Cc* V170, respectively) are buried, they are located near the inter-protofilament interface and residue I126, in particular, lies within the critical dimerization helix (α3) (**Fig 8)** [15].

The Lowe group demonstrated that the interaction of MreB protofilaments via their flat sides is necessary for proper MreB function in *E*. *coli* [15]; thus, if CbtA is indeed interacting with the flat side of MreB, it may be blocking MreB function by preventing the formation of the essential double filament. To further evaluate the proposition that CbtA interacts with the flat side of MreB, we performed a targeted mutagenesis of other residues located on its flat side. Charge reversal substitutions were introduced at several positions (e.g. D192K) and non-conservative changes were made at additional positions (e.g. F84A). The ability of each MreB mutant (bearing a single amino acid substitution) to interact with both CbtA and RodZ_NTD_ was assessed in our two-hybrid system. **S1 Table** summarizes the two-hybrid interaction profiles of the complete set of MreB variants that was tested.

Among those MreB variants tested (as α-MreB fusion proteins), we identified three additional inter-protofilament interface mutants with altered CbtA-binding. The mutants α-MreB-F84A and α-MreB-D192K were unable to interact with λCI-CbtA, but maintained strong interaction with λCI-RodZ_NTD_ (**Fig 7A**). Conversely, the α-MreB-S269F variant was greatly increased in its ability to interact with λCI-CbtA, yielding a 25-30-fold increase in *lacZ* expression as compared to the highest empty vector control (**Fig 7B**, **S4B Fig**). This fold-change value is ~10 times higher than the 3-fold increase in *lacZ* expression consistently measured with wild-type α-MreB and λCI-CbtA. Importantly, the effect of the S269F substitution was specific to CbtA; α-MreB-S269F yielded a λCI-RodZ_NTD_ interaction profile identical to that of wild-type α-MreB across multiple induction levels (**Fig 7B**). Because MreB and CbtA are both known to interact with FtsZ [46], we considered the possibility that the S269F substitution might actually promote interaction between α-MreB and FtsZ; enhanced bridging of α-MreB-S269F and λCI-CbtA by endogenous FtsZ molecules could potentially lead to an apparent increase in the CbtA-MreB interaction. However, this explanation seems unlikely as α-MreB-S269F interacted similarly strongly with the λCI-CbtA-F65S variant, which is unable to interact with FtsZ **(S4B Fig**).

### Substitutions at the MreB double filament interface affect ability of CbtA to inhibit cell elongation

Single amino acid substitutions at various positions along the flat side of MreB (including several affecting residues that lie directly at the double protofilament interface) altered its interaction with CbtA in the context of our two-hybrid system. These data suggest that the MreB inter-protfilament interface may be the binding surface utilized by CbtA to inhibit cell elongation. To determine whether or not the MreB interface residues identified in our two-hybrid analyses are critical for the toxic block in cell elongation mediated by CbtA, we aimed to overproduce CbtA-F65S (whose toxicity derives exclusively from its ability to interact with MreB) in strains producing the various mutants as the sole source of endogenous MreB. Overproduction of CbtA-F65S results in a lethal (under rapid growth conditions) loss of rod shape, causing cells to become spherical and lyse. Accordingly, we predicted that in strains bearing MreB substitutions that disrupt the CbtA-MreB interaction, overproduction of CbtA-F65S would be less toxic and would not induce a spherical morphology. Additionally, we hypothesized that CbtA-F65S toxicity might be increased in a strain producing the “up” variant MreB-S269F, resulting in more severe growth defects and morphological perturbations. Importantly, this strategy required the use of MreB variants capable of supporting rod-shaped growth.

**Fig 9:**
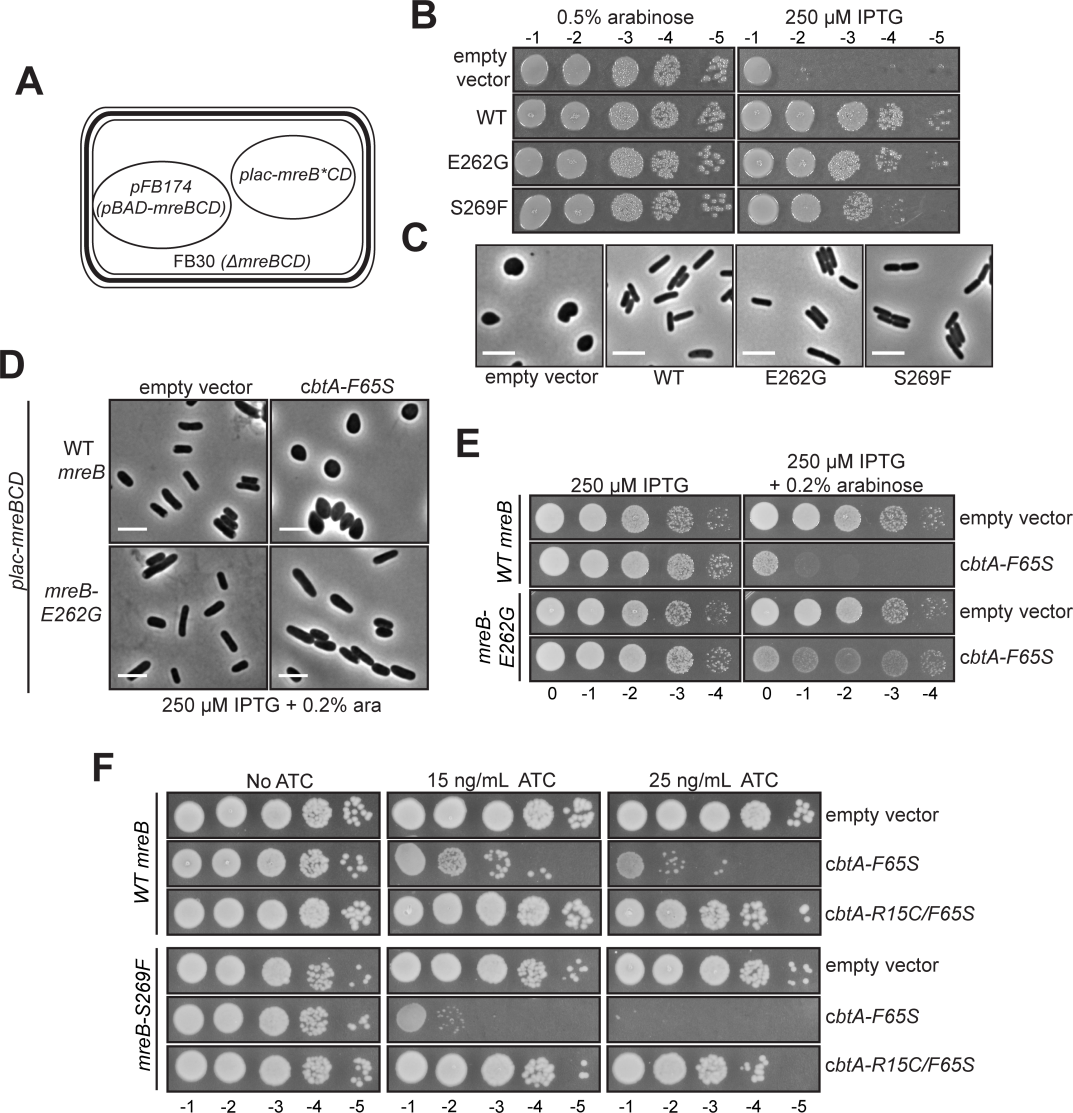
Residues on the flat surface of MreB are critical for CbtA-F65S-mediated cell elongation inhibition. (A) Schematic of MreB complementation assay (panels B and C). Depletion strain FB30/pFB174 (*mreBCD*::*kan*^*R*^*/pBAD-mreBCD)* [9] was transformed with either an empty vector control (pMLB1113) or a plasmid derived from pFB149 (*plac-mreBCD*) [9] expressing wild-type *mreB* (pFB149), *mreB-E262G* (pDH279), or *mreB-S269F* (pDH332). (B) Spot dilution assay to assess the ability of each *mreB* allele to support growth on solid medium. Cultures of each strain grown overnight at 37°C in M9 maltose (0.2% maltose, 0.2% casamino acids, 1 mM MgSO_4_) supplemented with 0.5% arabinose were back-diluted 1:100 in fresh M9 maltose + 0.5% arabinose and grown at 37°C for several hours until they reached late log phase. Cultures were pelleted and washed to remove arabinose, normalized to the same OD600 value, serially diluted, and spotted on LB plates supplemented with either 0.5% arabinose or 250 μM IPTG. Plates were incubated overnight at 37°C. Dilutions 10^−1^ to 10^−5^ are shown. (C) Cell morphology phenotypes of complemented strains. Aliquots from the same overnight cultures described in (B) were washed once and resuspended in fresh M9 maltose (without arabinose). These washed aliquots were used as inocula for M9 maltose cultures supplemented with 250 μM IPTG. All cultures were grown at 37°C for several hours (maintained in log-phase by back dilution) and cell morphology was monitored periodically by microscopy. Images taken after 5 h of growth in the presence of 250 μM IPTG are shown. (D) Effect of MreB substitution E262G on cell morphology phenotypes in the presence or absence of overproduced CbtA-F65S. Strains DH118/pFB149 (BW27785 *mreBCD*::*kan*^*R*^/ *plac-mreBCD*) and DH118/pDH278 (BW27785 *mreBCD*::*kan*^*R*^/ *plac-mreB-E262G mreCD*) were transformed with either the empty vector pBAD33 control plasmid or pBAD33-*cbtA-F65S* (pDH212). Transformants were selected on M9 maltose (0.2% maltose 0.2% casamino acids 1 mM MgSO_4_) plates supplemented with appropriate antibiotics and 250 μM IPTG at 30°C. Overnight cultures were grown in M9 maltose + 250 μM IPTG at 30°C. These overnight cultures were back diluted to a starting OD600 of 0.03 in LB (Cm + Carb) supplemented with 250 μM IPTG, and grown for 1 h at 30°C (reaching an OD600 ~0.08). Cultures were induced by addition of 0.2% arabinose and grown for an additional 2 h at 30°C, at which point microscopic analysis was performed. (E) Effect of MreB substitution E262G on cell viability in the presence or absence of overproduced CbtA-F65S. Aliquots from the same overnight cultures described in (D) were back diluted 1:100 in M9 maltose + 250 μM IPTG and grown at 30°C for ~5 h (until cultures had reached an OD600 of ~0.7). Cultures were normalized to the same OD600 value, serially diluted, and spotted on LB plates supplemented with 250 μM IPTG ± 0.2% arabinose. Plates were incubated for 48 h at RT. Dilutions 10^0^ to 10^−4^ are shown. (F) Effect of MreB substitution S269F on cell viability in the presence or absence of an overproduced CbtA variant. Strains DH118/pFB149 and DH118/pDH332 (BW27785 *mreBCD*::*kan*^*R*^/ *plac-mreB-S269F mreCD*) were transformed with empty vector pSG369, pDH335 (*ptet-cbtA-F65S*), or pDH337 (*ptet-cbtA-R15C/F65S*). Spot dilution cultures were prepared as described above for panel (E) and spotted on LB (Spec + Strep) plates supplemented with 250 μM IPTG ± 15 ng/mL or 25 ng/mL ATC. Plates were incubated at 37°C for 48 h. Dilutions 10^−1^ to 10^−5^ are shown. In all microscopy images, scale bars represent 5 μm.

We tested the abilities of several of our isolated mutant alleles to complement the growth and morphological defects of an *mreBCD* depletion strain (FB30/pFB174) when expressed in an IPTG-dependent manner from a multi-copy plasmid along with operon partners *mreC* and *mreD* (**Fig 9A)** [9]. Only cells expressing *mreB-E262G* or *mreB-S269F* were able to support growth to a similar extent as the wild-type allele (**Fig 9B)**; strains expressing these mutant alleles also maintained a rod shape comparable to that of the wild-type *mreB-*expressing strain (**Fig 9C**). We thus proceeded with these two alleles, testing the effect of overproducing CbtA-F65S in cells containing wild-type *mreB*, *mreB-E262G* or *mreB-S269F* as the sole source of MreB (see Materials and Methods). All three strains exhibited similar growth rates in liquid medium (**S4C Fig**), and Western blot analysis of these strains using an MreB antibody indicated that both mutant proteins were produced at levels comparable to that of the wild-type MreB protein (**S4D Fig**).

To test the effect of disruptive substitution E262G, we transformed our wild-type and *mreB-E262G* strains with either a plasmid producing CbtA-F65S (untagged) under the control of an arabinose-inducible promoter or an empty vector control and monitored cell growth and cell morphology in the presence of arabinose. Cells of both strains bearing the empty vector maintained rod shape in the presence of arabinose, and, as expected, cells containing wild-type MreB and the CbtA-F65S plasmid became spherical within two hours of arabinose addition (**Fig 9D**). In marked contrast, cells containing MreB-E262G did not become round, maintaining rod-like shape after two hours of arabinose addition (**Fig 9D**); CbtA-F65S-dependent growth inhibition was also reduced in the *mreB-E262G* strain (**Fig 9E**). Thus, MreB substitution E262G, which lies at the double protofilament interface and disrupted the two-hybrid interaction between MreB and CbtA, also interfered with the ability of CbtA-F65S in inhibit cell elongation.

To test the effect of substitution S269F, which strengthened the two-hybrid interaction between MreB and CbtA, we transformed our wild-type and *mreB-S269F* strains with either a plasmid producing CbtA-F65S (untagged) under the control of a tetracycline-inducible promoter or an empty vector control. We used a tetracycline-inducible system for these experiments to achieve a finer range of CbtA-F65S concentrations that might enable us to observe a wider range of growth and morphology phenotypes. Nonetheless, we did not observe any obvious morphological differences between these two strains at either 30°C or 37°C at multiple anhydrous-tetracycline (ATC) concentrations; both strains similarly transitioned from rod-shaped to spherical cells over the course of one hour (**S4E Fig**). However, we did see a more pronounced CbtA-F65S-dependent growth defect in the *mreB-S269F* strain (**Fig 9F**). In particular, expression of *cbtA-F65S* at a low ATC concentration (15 ng/mL) in the wild-type *mreB* strain caused a relatively modest growth defect on LB agar at 37°C, whereas expression of *cbtA-F65S* at the same ATC concentration in the *mreB-S269F* strain resulted in a 2-3-log decrease in plating efficiency (**Fig 9F**). Expression of the *cbtA-R15C/F65S* double mutant (recall that substitution R15C specifically disrupts the interaction of CbtA with MreB) had no effect on the growth of either strain, confirming that the increased toxicity of CbtA-F65S in the *mreB-S269F* background was dependent on the CbtA-MreB interaction (**Fig 9F**). Thus, MreB substitution S269F, which lies at the double protofilament interface and substantially strengthened the CbtA-MreB two-hybrid interaction, also sensitized MreB to the toxic cell elongation block mediated by CbtA-F65S. Taken together, our analyses of the MreB-E262G and MreB-S269F variants strongly suggest that the flat surface of MreB is critical for CbtA-dependent cell elongation inhibition and likely forms the inhibitory surface directly targeted by CbtA.

### CbtA homologs YkfI and YpjF target FtsZ and MreB in a conserved manner

**Fig 10:**
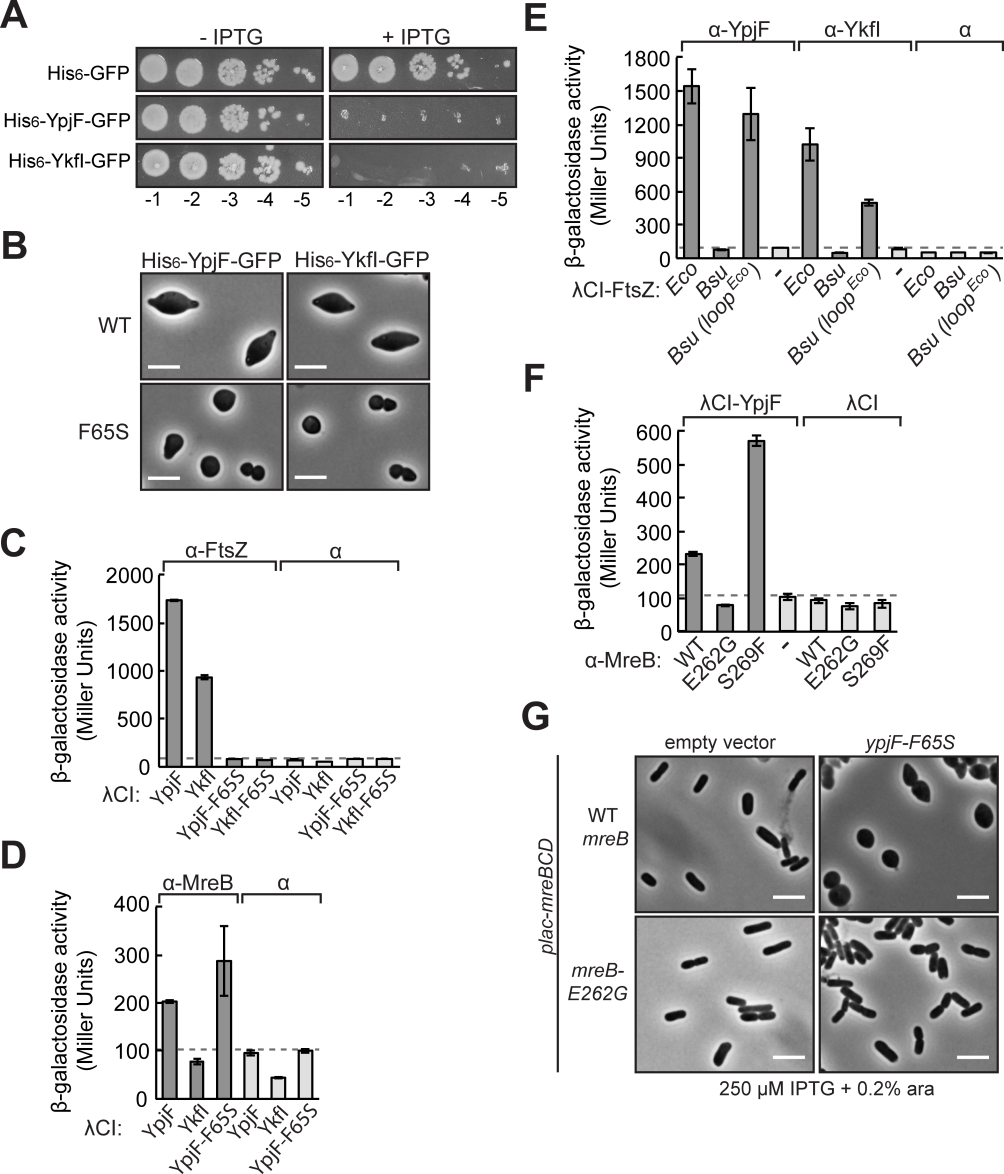
CbtA homologs YpjF and YkfI inhibit cell elongation and cell division in a conserved manner. (A) Spot dilution analysis shows that overproduction of His_6_-YpjF-GFP and His_6_-YkfI-GFP from pCA24N-derived plasmids pMT138 and p3-37, respectively, is toxic. Overnight cultures of BW27785/pMT136 (directing the synthesis of His_6_-GFP), BW27785/pMT138, and BW27785/p3-37 were back diluted to a starting OD600 of 0.05 in fresh LB (Cm) and grown until late-log phase at 37°C. Cultures were normalized, serially diluted, and spotted on LB (Cm) with or without 100 μM IPTG. Plates were incubated at 37°C overnight. Dilutions 10^−1^ to 10^−5^ are shown. (B) Cell morphology phenotypes. Strains BW27785/pMT138 (*his_6_-ypjF-gfp)*, BW27785/pMT188 (*his_6_-ypjF-F65S-gfp*), BW27785/p3-37 (*his_6_-ykfI-gfp)*, or BW27785/pMT144 (*his_6_-ykfI-F65S-gfp*) were imaged after 1.5 h induction at 37°C with 100 μM IPTG. (C, D) Bacterial two-hybrid assay detects interactions of YpjF and YkfI with FtsZ or MreB. Results of β-galactosidase assays performed with reporter strain cells containing compatible plasmids encoding the indicated λCI fusion protein and the indicated α fusion protein (α-FtsZ in (C) and α-MreB in (D)) or wild-type α. Cells were grown in the presence of 25 μM IPTG (C) and 100 μM IPTG (D). (E) Two-hybrid analysis shows that the *Bsu* FtsZ chimera containing the *Eco* H6/H7 loop (fused to λCI) can interact with both YkfI and YpjF (fused to α). Cells were grown in the presence of 100 μM IPTG. (F) Effect of MreB substitutions E262G and S269F on the two-hybrid interaction between MreB and YpjF. Cells were grown in the presence of 100 μM IPTG. In (C-F), bars represent averages of triplicate values, error bars represent standard deviation, and dashed lines designate highest basal *lacZ* expression. (G) Strains DH118/pFB149 and DH118/pDH278 (see legend to **Fig 9D**) were transformed with either the empty vector pBAD33 control plasmid or pBAD33-*ypjF-F65S* (pDH289). Transformants were selected on M9 maltose (0.2% maltose, 0.2% casamino acids, 1 mM MgSO_4_) plates supplemented with appropriate antibiotics and 250 μM IPTG at 30°C. Overnight cultures were grown in M9 maltose + 250 μM IPTG at 30°C. The next morning, cultures were back diluted to a starting OD600 of 0.03 in LB (Cm + Carb) supplemented with 250 μM IPTG, and grown for 1 h at 30°C (reaching an OD600 ~0.08). Cultures were induced by addition of 0.2% arabinose and grown for an additional 2 h at 30°C, at which point microscopic analysis was performed. Scale bars represent 5 μm in (B) and (G).

CbtA has two homologs in *E*. *coli*: the YkfI toxin of the YkfI/YafW toxin-antitoxin system, and the YpjF toxin of the YpjF/YfjZ toxin-antitoxin system [38]. The three toxins are encoded on different cryptic prophage elements within the *E*. *coli* genome, and have high amino acid sequence identity (58% identity between CbtA and YkfI, 62% identity between CbtA and YpjF, 78% identity between YpjF and YkfI) [38](**S5A Fig**). Overexpression of either *ykfI* or *ypjF* was previously shown to be toxic [38,70] and results in the formation of lemon-shaped cells [70]. Consistent with these previous results, we found that overexpression of *his_6_-ypjF-gfp* or *his_6_-ykfI-gfp* under the control of the hybrid *pT5-lac* promoter resulted in a decrease in viability (**Fig 10A**) and led to the formation of lemon-shaped cells (**Fig 10B**). We were also able to detect strong interactions between both toxins and FtsZ (**Fig 10C**), and between YpjF and MreB in our bacterial two-hybrid system (**Fig 10D**). Although YkfI is 78% identical to YpjF and blocks cell elongation when overproduced, we were unable to detect an interaction between YkfI and MreB in our bacterial two-hybrid system.

In order to determine whether YkfI and YpjF interact independently with FtsZ and MreB and utilize the same inhibitory surfaces as CbtA, we repeated many of the two-hybrid analyses described above. Residue F65 is conserved in all three toxins (**S5A Fig**), and as we saw with CbtA-F65S, overproduction of YpjF-F65S and YkfI-F65S yielded sphere-like rather than lemon-shaped cells (**Fig 10B**). Furthermore, we found that substitution F65S disrupted the two-hybrid interactions between YpjF and FtsZ and between YkfI and FtsZ (**Fig 10C**), but did not compromise the two-hybrid interaction between YpjF and MreB (**Fig 10D**). These analyses suggest that the FtsZ interaction determinants for all three toxins are conserved. Importantly, the morphology data also suggest that toxin interaction with FtsZ contributes to the striking lemon-shape phenotype. Interestingly, R15 is not a conserved residue; YpjF and YkfI both have a cysteine at this position (**S5A Fig**). Because substitution R15C decreased the interaction between CbtA and MreB, we wondered if the reverse substitution (C15R) in YpjF would increase its interaction with MreB, and in the case of YkfI, might allow for a detectable interaction. We found that substitution C15R had no effect in either case (**S5B Fig**). Thus, the genetic determinants within YpjF and YkfI that specify their abilities to inhibit cell elongation remain unknown.

To assess whether residues in the H6/H7 loop are necessary for the YpjF-FtsZ and YkfI-FtsZ interactions, we assayed the abilities of YpjF and YkfI to interact with wild-type *Bsu* FtsZ and our *Bsu* FtsZ chimera. We found that neither YkfI nor YpjF interacted with wild-type *Bsu* FtsZ, but both toxins interacted strongly with the *Bsu* FtsZ chimera containing the H6/H7 loop of *E*. *coli* (*Bsu ftsZ (loop*^*Eco*^*)*) (**Fig 10E**). These findings provide strong support for the idea that all three homologous toxins interact directly with the H6/H7 loop of *E*. *coli* FtsZ. Similarly, as was seen with CbtA, single amino acid substitutions affecting residues on the flat side of MreB altered its interaction with YpjF. The E262G substitution disrupted the YpjF-MreB interaction, whereas the S269F substitution more than doubled the detected interaction (**Fig 10F**). Furthermore, we found that a strain harboring the *mreB-E262G* allele was less susceptible than the corresponding wild-type *mreB* strain to cell shape changes induced by YpjF-F65S (**Fig 10G**), suggesting that CbtA and YpjF both require residues lying at the double protofilament interface to inhibit the function of MreB. Taken together, these results strongly suggest that like CbtA, YpjF and YkfI act as dual inhibitors that block cell division and cell elongation in a genetically separable manner; furthermore, all three toxins appear to inhibit these processes by targeting the same surfaces of FtsZ and MreB.

## Discussion

We have shown that the chromosomally encoded toxin CbtA inhibits cell division and cell elongation via independent and genetically separable interactions with FtsZ and MreB. In particular, we identified amino acid substitutions in CbtA that specifically disrupt its interaction with FtsZ on the one hand (F65S), and MreB on the other (R15C), and we showed that both interactions contribute to CbtA toxicity. This genetic analysis enabled us to rule out the possibility that CbtA exerts its effect on either cell division or cell elongation indirectly, by interfering with a functionally relevant interaction between FtsZ and MreB [46]. Furthermore, we identified amino acid substitutions in FtsZ and MreB that disrupt their respective interactions with CbtA, and we constructed strains bearing one or the other type of substitution. This analysis defined the surfaces of FtsZ and MreB targeted by CbtA to inhibit cell division and cell elongation, respectively. Based on the location of these surfaces, we suggest that CbtA may block FtsZ polymerization and MreB double filament formation.

### CbtA-FtsZ interaction defines H6/H7 loop as new inhibitory target

Our analysis of the interaction between CbtA and FtsZ uncovered the H6/H7 loop as a new target for inhibitors of FtsZ function. In particular, using our two-hybrid assay to screen for FtsZ mutations that specifically disrupted its interaction with CbtA, we found that the identified mutations mapped to the H6/H7 loop. We then showed that the identities of loop residues dictated whether or not CbtA could inhibit cell division both in *E*. *coli* and in *B*. *subtilis*. Finally, we identified an amino acid substitution in CbtA (V48E) that functioned as an allele-specific suppressor of a disruptive charge reversal substitution in the FtsZ H6/H7 loop (D180K), providing strong evidence for a direct physical interaction between CbtA and the H6/H7 loop. FtsZ subunits assemble as protofilaments by stacking vertically in a head-to-tail fashion, and the H6/H7 loop lies at the longitudinal interface formed by pairs of stacked subunits (**see Fig 4B**) [64,66,67]. We thus suggest that CbtA likely exerts its inhibitory effect on cell division by interfering with FtsZ protofilament formation. Additionally, residues in the H6/H7 loop have been implicated in FtsZ lateral interactions and bundling [71,72], raising the possibility that CbtA could also inhibit such higher order interactions of FtsZ. We note that the cognate antitoxin of CbtA, CbeA (previously YeeU), has been shown to interact with both FtsZ and MreB and stabilize protofilament bundling *in vitro*, suggesting that it neutralizes CbtA toxicity *in vivo* by stabilizing a higher order assembly of each of its targets, rather than by interacting directly with the toxin [33]. Interestingly, CbeA was also found to neutralize the toxicity of several other protein inhibitors of FtsZ function with distinct modes of action, suggesting that CbeA’s ability to stabilize a higher order assembly of FtsZ has a general protective effect [33].

Our findings do not support the previous proposal that CbtA interacts with the C-terminal region of FtsZ. Specifically, we found that removal of this C-terminal region (FtsZΔ66; **Fig 4A**) had no effect on the CbtA-FtsZ interaction, in contrast with previously reported results [32]. The FtsZ monomer consists of a globular core comprising two independently folded domains separated by α-helix H7; appended to this globular core is an unstructured linker region terminating in the highly conserved 15-residue C-terminal tail (CTT). Like their tubulin counterparts, the FtsZ N-terminal domain binds GTP, whereas the C-terminal domain contains the so-called synergy (T7) loop, which stimulates GTP hydrolysis in the context of an assembled protofilament by contacting the GTP-binding pocket of the next subunit. Specifically, the vertical stacking of FtsZ subunits enables the T7 loop of one subunit to insert into the GTP-binding pocket of the subunit just beneath [64] (see **Fig 4B**). Most previously characterized regulators of FtsZ assembly, including inhibitory factors such as SlmA [53,55] and the C-terminal domain of MinC [62], bind to the CTT, establishing it as an important hub of regulation [54,56–61]. Other protein regulators, such as SulA, the *B*. *subtilis* sporulation factor MciZ, and the N-terminal domain of MinC bind within the C-terminal domain (for example, in the vicinity of the T7 loop) and inhibit FtsZ assembly through diverse mechanisms [28,73–75]. CbtA and its homologs YpjF and YkfI provide the first example of protein inhibitors that target the FtsZ N-terminal domain, binding the H6/H7 loop.

The identification of an FtsZ inhibitor that binds to the H6/H7 loop could facilitate studies aimed at probing the polarity of protofilament assembly and disassembly. Both the GTP-binding pocket and the H6/H7 loop are located at the FtsZ plus end (defined by analogy with tubulin), whereas the T7 loop is located at the minus end [64,76]. Evidence from one study, in which plus-end and minus-end mutants were tested for their abilities to function as FtsZ cappers, suggested that FtsZ filaments assemble and disassemble with a polarity opposite that of microtubules, with FtsZ subunits being added primarily to the minus end and dissociating primarily from the plus end [76]. However, evidence from a more recent study, in which the sporulation-specific Z ring inhibitor MciZ was defined as a minus-end capper, was more consistent with designation of the plus end as the primary addition site [74]. CbtA, a plus-end binder, could provide a useful new tool for addressing this problem.

### CbtA targets MreB double filament interface to inhibit cell elongation

Our genetic analysis of the CbtA-MreB interaction identified amino acid substitutions that mapped to the flat side of the MreB monomer. Specifically, we identified six substitutions affecting flat-side residues (F84A, I126V, V173A, D192K, E196G, E262G) that disrupted and one (S269F) that strongly increased the two-hybrid interaction between CbtA and MreB. Among the affected residues, F84, E196, and E262 are surface-exposed, suggesting that they may contact CbtA directly; in addition, S269 is surface-exposed, suggesting that the mutant phenylalanine residue may form a new interaction at the CbtA-MreB interface. In the case of residue D192, which lies in a small pocket beneath residue E196, we speculate that the introduction of a positively charged lysine residue in its place may alter the position of surface-exposed E196, indirectly perturbing its interaction with CbtA. We found that cells containing either MreB-E262G or MreB-S269F (in the absence of wild-type MreB) grew as rods, enabling us to assess the effects of these substitutions on CbtA-dependent morphological changes, as well as CbtA-dependent growth inhibition. Our findings indicated that MreB substitution E262G mitigated the effects of CbtA on growth and cell shape, whereas MreB substitution S269F potentiated the toxic effect of CbtA (**Fig 9**). Taken together with the two-hybrid data, these findings support the idea that CbtA inhibits cell elongation by binding directly to the flat surface of MreB.

The discovery that pairs of MreB protofilaments associate in an antiparallel fashion along their flat sides to form a double filament that is required for MreB function in *E*. *coli* [15] leads us to propose that CbtA inhibits cell elongation by interfering with double filament formation. This proposed mechanism appears to be shared by other MreB inhibitors, as well. The small molecule inhibitor A22 (and its derivative MP265) was found to prevent double filament formation, evidently by displacing the main dimerization helix (α-helix 3 formed by Q120 to A133) that participates in essential inter-protofilament contacts (though the inhibitors also block nucleotide hydrolysis in the active site) [15]. Moreover, a recent study of *B*. *subtilis* sporulation factors YodL and YisK suggests that they can influence cell shape by targeting MreB and the MreB-like protein Mbl, respectively; specifically, Mbl substitution E250K (affecting the residue corresponding to E262 in *E*. *coli* MreB) was found to suppress the cell-shape defects caused by YisK [37]. Assuming that Mbl adopts a similar double-filament architecture as *E*. *coli* MreB, YisK may also disrupt double filament formation.

### CbtA as a dual-function cytoskeletal inhibitor

CbtA is the first example of a cytoskeletal inhibitor capable of independently targeting the cell division and cell elongation apparatuses. Given its small size (124 amino acids), CbtA’s ability to function as a dual inhibitor is particularly striking. It will be interesting to learn whether a single toxin molecule can interact simultaneously with FtsZ and MreB or whether inhibition of cell division and cell elongation depends on the combined action of subsets of molecules that interact with one or the other target. Whereas our work sheds light on the molecular basis for the effects of CbtA and its homologs YpjF and YkfI on cell shape and cell growth, it remains to be learned what roles these toxins might play in cellular physiology. A recent study reported that an *E*. *coli* strain deleted for all three toxin genes exhibited increased susceptibility to oxidative stress [70], raising the possibility that these toxin-antitoxin systems, like other chromosomally encoded toxin-antitoxin systems, contribute to the bacterial stress response. As these toxins are encoded on cryptic prophages, it is also interesting to consider what roles they might have played in the context of phage biology. A number of phage-encoded factors are known to block bacterial cell division, some of which (for example, the Kil peptide of bacteriophage λ) have been shown to target FtsZ [29]. It is of particular interest to note that the lytic phage T7 has recently been shown to encode separate inhibitors of FtsZ (Gp0.4) and MreB (Gp0.6) [30,36], one of which (Gp0.4) was shown to provide a growth advantage to the phage in dividing cells [30]. Kiro et al. [30] suggest that inhibiting cell division early after infection ensures that all the cell’s resources are available for phage replication by preventing daughter-cell escape. Whether or not the changes in cell size, cell shape and/or cell wall integrity that result from the combined inhibition of FtsZ and MreB have the potential to enhance phage production remains to be investigated.

### Implications for antibiotic development

FtsZ has been validated as a clinically relevant antibiotic target [31]. Our identification of its H6/H7 loop as a new epitope that can be exploited by FtsZ inhibitors may therefore have implications for the development of new antibiotics that target this essential protein. More speculatively, future structural studies of CbtA in complex with its partners could potentially inform the design of antibiotics that target both FtsZ and MreB (the latter also representing a potentially effective target for antibacterial agents). Such dual-function agents would be attractive due to the greater barrier towards the development of resistance.

## Materials and methods

### Overview of strains, growth conditions, and plasmids

A complete list of the bacterial strains used in this chapter is provided in **S2 Table**. Additionally, lists of the plasmids and oligonucleotides used in this chapter can be found in **S3 and S4 Tables**, respectively. NEB5-α F’I^q^ (New England Biolabs) was used as the cloning strain for all plasmid constructions outlined below. Two-hybrid studies were performed in FW102 O_L_2-62 [77] or BN30 [78]. Morphology observations were made primarily in strain BW27785 [79,80]. This strain also served as template for all colony PCRs. ZapA-GFP microscopy was performed using strain NP1 [50]. GFP-FtsZ microscopy was performed using strain TB28 HKHC488; this strain contains wild-type *ftsZ* at the endogenous locus and an IPTG-inducible allele of *sfgfp-ftsZ* (encoding FtsZ fused to superfolder GFP) integrated at the attHK site. *E*. *coli* strains were grown in LB (1% NaCl) broth at 37°C or 30°C, and on LB plates supplemented with appropriate antibiotics at the following concentrations (unless otherwise noted): carbenicillin (Carb), 100 μg/mL; chloramphenicol (Cm), 25 μg/mL; kanamycin (Kan), 50 μg/mL; spectinomycin (Spec), 50 μg/mL; streptomycin (Strep), 25 μg/mL; tetracycline (Tet), 5 μg/mL. Where noted, strains were grown in M9 minimal liquid medium or M9 agar (1 mM MgSO_4_) supplemented with either 0.4% maltose and 0.01% casamino acids, or 0.2% maltose and 0.2% casamino acids.

*B*. *subtilis* strains were grown at 37°C in LB (0.5% or 1% NaCl) broth without antibiotic or on LB plates supplemented with spectinomycin (100 μg/mL) or MLS (mixture of 1 μg/mL erythromycin and 25 μg/mL lincomycin). *B*. *subtilis* PY79 genomic DNA was used as template for all *B*. *subtilis ftsZ* constructs.

### Plasmid constructions

p3-37 is a derivative of the ASKA overexpression vector, pCA24N [47], encoding His_6_-YkfI-GFP under the control of the *pT5-lac* promoter. In this construct, *his*_*6*_*-ykfI-gfp* contains two SfiI sites flanking the *ykfI* sequence. Empty vector plasmid pMT136 encoding His_6_-GFP was made by cloning in a linker sequence composed of annealed oligonucleotides, oSG623 and oSG624, into SfiI-digested p3-37. This linker sequence contains ClaI and XbaI sites and encodes for the additional residues “IDAAASR” in between the SfiI sites in the His_6_-GFP sequence. To construct plasmids pMT138 (encoding His_6_-YpjF-GFP) and pMT139 (encoding His_6_-CbtA-GFP), colony PCR products generated using primer pair oSG639/oSG640 or oSG641/oSG642, respectively, were digested with AclI and XbaI and ligated into pMT136 digested with ClaI and XbaI. Plasmids pMT144 (encoding His_6_-YkfI-F65S-GFP) and pMT146 (encoding His_6_-CbtA-F65S-GFP) were generated by ligation of AclI/XbaI-digested overlap PCR products amplified with internal mutagenic primers (oSG663/oSG664 and oSG667/oSG668) and outside primers (oSG659/oSG660 and oSG641/oSG642) into ClaI/XbaI-digested pMT136 backbone. To construct plasmid pDH253, the *cbtA-R15C* allele was amplified from the two-hybrid construct pDH246 using primers oSG641 and oSG642, digested with AclI and XbaI, and ligated into pMT136 ClaI/XbaI backbone. Plasmid pDH262 (encoding His_6_-CbtA-R15C/F65S-GFP) was constructed in the same manner as pMT146 except using pDH253 as PCR template.

All bacterial two-hybrid α fusion constructs were cloned by restriction digest into the parent plasmid pBRα-β flap; all two-hybrid λCI fusion constructs were cloned by restriction digest into the parent plasmid pACλCI-β flap. Briefly, the parent plasmids were digested with NotI and BamHI to generate backbone. These backbones were ligated to relevant inserts generated by NotI/BamHI digestion of PCR products amplified using a NotI-containing forward primer and BamHI-containing reverse primer. Forward primers all contain an extra “A” base after the NotI site to maintain the reading frame. Reverse primers all encode a stop codon preceding the BamHI site. Mutant alleles (both point mutants and chimeric alleles) of *ftsZ*, *mreB*, or toxin genes were generated using internal mutagenic primers (see **S4 Table** for specific sequences). Oligonucleotides pBRα_F, pBRα_R, pACλCI_F, and pACλCI_R were used to sequence all two-hybrid constructs.

To generate plasmids pDH325, pDH326, pDH327, and pDH328 encoding untagged CbtA variants, the relevant *cbtA* allele was amplified from the appropriate pCA24N-derived construct described above using primers oDH446 and oDH447, digested with EcoRI/HindIII, and ligated into pSG360 (EcoRI/HindIII) backbone.

To construct *cbtA-F65S* and *ypjF-F65S* arabinose-inducible overexpression vectors (pDH212 and pDH289), alleles were amplified from pMT146 and pMT188 using primer pair oDH285/oDH286 or oDH380/381, respectively. PCR products were digested with NdeI/XbaI and ligated into the pBAD33 (NdeI/XbaI) backbone. To construct *cbtA-F65S* and *cbtA-R15C/F65S* tet-inducible overexpression vectors (pDH335 and pDH337), the EcoRI/HindIII inserts from plasmids pDH326 and pDH328 were ligated into pSG369 (EcoRI/HindIII) backbone.

pFB149 (*plac-mreBCD lacI*^*Q*^) contains an XbaI site upstream of *mreB* and a naturally occurring BamHI site within *mreD* that is unique on plasmid pFB149. To construct pFB149-derivatives for MreB mutant expression studies, *mreB* mutant alleles were generated by overlap PCR using pFB149 as template, outside primers oDH372/oDH369, and allele-specific internal mutagenic primers. PCR products were digested with XbaI/BamHI and ligated into the pFB149 (XbaI/BamHI) backbone. All pFB149- derivatives were verified by sequencing using primers oDH355, oDH369, oDH373, oDH374, and oDH375.

### *E*. *coli* strain constructions

(The *ftsZ-L169P* allele was cloned into pCX41 (digested with HindIII/ClaI) in place of wild-type *ftsZ* by restriction digest (HindIII/ClaI) and ligation of overlap PCR products generated using wild-type pCX41 as template, internal mutagenic primers (oDH34_F and oDH35_R for L169P) and flanking primers oDH36_F, oDH37_R, which anneal within *ftsA* and *lpxC*, respectively. This generated plasmid pDH35, replication of which is controlled by a temperature-sensitive origin of replication. Plasmid is maintained at 30°C and lost at 42°C. Attempted integration of these mutant alleles into the endogenous chromosomal locus was performed essentially as described in [81]. Briefly, pDH35 was transformed into *E*. *coli* strain BW27785 in parallel with a pCX41 derivative encoding FtsZ-F268C. *ftsZ-F268C* is a known complementing allele and thus serves as a control for chromosomal integration. Transformants were plated on LB agar supplemented with Cm (10 μg/mL) and incubated overnight at 30°C. Several colonies were restreaked onto LB (Cm) and incubated at 42°C overnight in order to identify single crossover integrants. After an additional round of restreaking on LB (Cm) at the nonpermissive temperature, candidates were streaked onto LB (Cm) and incubated at 30°C overnight. Firing of the plasmid origin of replication on the chromosome causes a severe growth defect, and double crossover integrants that had looped out the plasmid were identified as healthy revertants within poorly growing streaks. These candidates were purified by restreaking, and were cured of plasmid by growth on LB (without Cm) at 42°C. The *ftsZ* locus was PCR amplified and sequenced (using sequencing primers generously provided by H. Cho and T. Bernhardt) from Cm-sensitive candidates in order to identify those in which allelic replacement occurred. All subsequent propagation of this strain was done at RT or 30°C to minimize growth defects. Multiple isolates of this strain exhibited identical phenotypes.

To construct strains DH118/pFB149, DH118/pDH278, and DH118/pDH332, plasmids pFB149, pDH278, and pDH332 were transformed into strain BW27785. To introduce the *mreBCD*::*kan*^*R*^ deletion, a P1 lysate was grown on strain FB30/pFB174 and used to infect each recipient strain. Transductants were selected on M9 maltose plates (0.2% maltose, 0.2% casamino acids, 1 mM MgSO_4_) supplemented with 5 mM sodium citrate and 250 μM IPTG (for expression of *mreBCD*). Growth on minimal medium is known to suppress *mreBCD* defects and was used to prevent acquisition of suppressor mutations. Strains were checked for proper *kan*^*R*^ insertion by colony PCR using primers oDH289 and oDH307. We note that in all DH118 strains, about 5% of cells failed to grow as rods, forming large spheres (as observed by microscopy); this is likely the result of plac-*mreBCD* plasmid loss. Growth of DH118 strains was monitored at 37°C over the course of 4 h. Four replicate M9 maltose overnight cultures were back diluted to a starting OD600 of 0.02 in 200 μL LB (Carb) supplemented with 250 μM IPTG in a 96-well microtitre plate. The plate was incubated shaking at 900 rpm in 90% humidity in a Multitron incubation shaker (Infors HT); OD600 readings were taken every 30 min with a microtitre plate reader (Molecular Devices).

### *B*. *subtilis* strain constructions

*B*. *subtilis* strains were generated by directly transforming a PY79 derivative with either a linearized plasmid containing homology to the chromosomal locus where integration was desired or a PCR fragment containing chromosomal homology. In order to generate *B*. *subtilis* strains with *gfp* or various *cbtA* alleles integrated into the chromosome, plasmids pDH84 (*pHYPERSPANK-his*_*6*_*-gfp*), pDH85 (*pHYPERSPANK-his*_*6*_*-cbtA-gfp)*, and pDH102 (*pHYPERSPANK-his*_*6*_*-cbtA-F65S-gfp)* were constructed. These plasmids were generated by restriction digest (HindIII/NheI) and ligation of PCR products amplified from pMT136, pMT139, or pMT146 using primers oDH108 and oDH116 into QER167 (generous gift of D. Rudner) HindIII/NheI digested backbone. These plasmids all contain homology to the *ycgO* locus flanking the insert. Plasmids were linearized by digestion with ScaI. DH84 (*ycgO*:: *pHYPERSPANK-his*_*6*_*-gfp erm*), DH85 (*ycgO*:: *pHYPERSPANK-his*_*6*_*-cbtA-gfp erm*), and DH104 (*ycgO*:: *pHYPERSPANK -his_6_-cbtA-F65S-gfp erm)* were generated by transformation of linearized plasmids pDH84, pDH85, and pDH102, respectively, into PY79 *ycgO*::*spec*. Transformants were selected on LB supplemented with MLS.

The wild-type *ftsZ* allele linked to a spec resistance cassette was assembled by Gibson assembly [82] of three PCR products with >20bp of overlapping homology: 1) part of the *ftsA* locus and the entire *ftsZ* locus amplified from PY79 genomic DNA using oligos oDH130 and oDH131, 2) amplification of *spec* from pDR111 using oDH132 and oDH133, and 3) 2 kb chromosomal sequence downstream of the *ftsZ* locus amplified from PY79 genomic DNA using oligos oDH134 and oDH135. This assembled PCR product was transformed directly into PY79 to generate strain DH98. Transformants were selected for on LB (Spec).

The chimeric *ftsZ* allele (containing *E*. *coli ftsZ* residues 169–182) linked to a spec resistance cassette, was assembled by Gibson assembly of three PCR products (all with at least 20 bp of overlapping homology: 1) 2 kb upstream of *ftsZ* amplified from PY79 genomic DNA using oligos ODH141 and ODH142, 2) the *ftsZ* chimeric allele amplified from pDH69 (*Bsu ftsZ (loop*^*Eco*^*)*) using oligos oDH143 and oDH131 3) 2 kb downstream of *ftsZ*, including *spec*, amplified from DH98 genomic DNA using oDH132 and oDH144. This assembled PCR product was transformed directly into PY79 to generate strain DH99. Transformants were selected for on LB (Spec). The *ftsZ* loci from DH98 and DH99 were PCR amplified (using oDH167 and oDH168) and sequenced using oDH127, oDH172, and oDH173. oDH124 and oDH125, which anneal inside the *ftsZ* ORF were also used for PCR and sequencing.

Strains DH100, DH101, and DH105 were generated by direct transformation of DH98 genomic DNA into strains DH84, DH85, and DH104, respectively. Strains DH102, DH103, and DH106 were generated by direct transformation of DH99 genomic DNA into DH84, DH85, and DH104, respectively. Transformants were selected on LB (Spec) and patched on LB (MLS) to ensure the *ycgO* locus was unchanged. The *ftsZ* loci were re-sequenced after transformation.

### Bacterial two-hybrid genetic screens

*cbtA* and *mreB* gene fragments (located on pMT154 and pMT151, respectively) were mutagenized by error-prone PCR using *Taq* polymerase (Promega) and the outside primers pACλCI_F and pACλCI_R. The *ftsZ* gene fragment (located on pMT153) was amplified using *Taq* polymerase and the outside primers pBRα_F and pBRα_R. The mutagenized *cbtA* alleles were cloned into the pAC-λCI fusion vector; mreB and ftsZ alleles were cloned into the pBRα fusion vector.

To identify λCI-CbtA variants with a decreased ability to interact with α-MreB, the *λCI-cbtA* mutant library was transformed into a modified two-hybrid reporter strain (BN30) bearing pBRα-MreB (pMT151). Strain BN30 contains an F’ episome bearing a two-hybrid reporter with the λCI operator positioned at -42, 20 bp closer to the transcription start site than in the standard two hybrid strain FW102 O_L_2-62. This positioning allows for an additional stabilizing contact between λCI and region 4 of σ70 bound to the -35 promoter element and results in an elevated level of *lacZ* expression [78], which afforded us a better color range for blue-white screening than our standard reporter. Transformants were plated on LB (KanCarbCm) indicator medium containing IPTG (25 μM) and X-gal (40 μg/mL). Plates were incubated overnight at 30°C and refrigerated (4°C) for an additional 8–16 h. α-MreB mutants with decreased λCI-CbtA interaction were isolated under identical conditions. For each screen, several thousand colonies were screened to identify those exhibiting lower *lacZ* expression (white or light blue color) as compared to the dark blue colonies producing wild-type α-MreB and λCI-CbtA fusions. For each screen, candidate mutants were counter-screened to identify those that maintained the ability to interact with a second partner protein (FtsZ, in the case of CbtA, and the NTD of RodZ, in the case of MreB). Specifically, colonies containing prospective λCI-CbtA mutants were pooled into a single overnight culture, grown at 30°C; a pooled plasmid prep generated from this overnight culture was transformed into FW102 O_L_2-62/pBRα-FtsZ. Transformants were plated on LB (KanCarbCm) indicator medium supplemented with 5 μM IPTG, 40 μg/mL X-gal, and 250 μM TPEG (a competitive inhibitor of β- galactosidase; Gold Biotechnologies); dark blue candidates were selected and the pACλCI-CbtA plasmids were isolated and fusion gene sequenced. For colonies containing prospective α-MreB mutants, individual cultures of candidate clones were grown overnight at 30°C. Plasmids were prepped from these cultures, most likely generating a mixed prep of α and λCI plasmids in each case. Individual mixed preps were used to transform FW102 O_L_2- 62 cells containing either pACλCI-CbtA or pACλCI-RodZ_NTD_. Transformants were selected on LB (CmCarbKan). β-galactosidase assays (see below) were performed to measure interaction between each α-MreB mutant and λCI-CbtA (at 100 μM IPTG) or λCI-RodZ_NTD_ (at 25 μM IPTG). pBRα-MreB plasmids were isolated from candidates that were down for λCI-CbtA interaction but maintained >60% of the wild-type λCI-RodZ_NTD_ interaction; the fusion genes were sequenced and the mutant plasmids re-tested by β-galactosidase assay.

To identify α-FtsZ mutants with a decreased ability to interact with λCI-CbtA, our *α-ftsZ* mutant library was transformed into FW102 O_L_2-62/pACλCI-CbtA; several thousand colonies were screened on medium containing 5 μM IPTG and X-gal (40 μg/mL) at 37°C. Colonies that were pale blue or white were selected and the plasmids were isolated and transformed into FW102 O_L_2-62 cells containing either pACλCI-CbtA or pACλCI-FtsZ. Transformants were selected on LB (CmCarbKan). β-galactosidase assays were performed to measure interaction between each α-FtsZ mutant and λCI-CbtA (at 100 μM IPTG) or λCI-FtsZ (at 100 μM IPTG). Those candidates that exhibited at least a 60% decrease in interaction with CbtA but maintained greater than 75% FtsZ-FtsZ self-interaction were sequenced and further assayed for their interaction with λCI-ZipA_CTD_ by β-galactosidase assay.

To identify CbtA variants with a restored ability to interact with FtsZ-D180K, the *λCI-cbtA* mutant library was transformed into FW102 O_L_2-62 cells pre-transformed with pBRα-FtsZ-D180K, and transformants were plated on LB (KanCmCarb) indicator medium supplemented with IPTG (5 μM), X-gal (40 μg/mL), and TPEG (250 μM) (Gold Biotechnology). Plates were incubated at 30°C overnight. Several thousand colonies were screened in order to identify those that exhibited increased *lacZ* expression as compared to the pale blue control colonies producing wild-type λCI-CbtA and α-FtsZ-D180K. Dark blue candidate colonies were pooled into a single overnight culture, grown at 30°C. In order to identify those candidates that specifically interact with α-FtsZ-D180K, a pooled plasmid prep generated from this overnight culture was transformed into FW102 O_L_2-62 cells containing pBRα-FtsZ. Transformants were plated on the same indicator medium as before; pale blue candidates were selected and the pACλCI-CbtA plasmids were isolated and the fusion genes sequenced. The interaction between λCI-CbtA-V48E and all relevant α-FtsZ fusions was assayed by β-galactosidase assay.

### β-galactosidase assays

All β-galactosidase assays were performed in our standard two-hybrid reporter strain, FW102 O_L_2-62. FW102 O_L_2-62 cells were co-transformed with plasmids encoding the relevant α and λCI fusions. Cultures inoculated with transformants were grown in 1 mL LB (KanCmCarb) in deep-well 96-well plates at 37°C, 900 rpm, 90% humidity in a Multitron incubation shaker (Infors HT) overnight. Overnight cultures were back diluted 1:100 or 1:40 in LB (KanCmCarb) supplemented with the appropriate concentration of IPTG in sterile microtitre plates (total volume of 200 μL); subcultures were grown, shaking at 37°C until they reached mid-log phase (OD600 0.4–0.8). A 100 μL aliquot of subculture was lysed by addition of 10 μL PopCulture reagent (Novagen) supplemented with rlysozyme (400 mU/μL). LacZ levels were determined by β-galactosidase assay performed in microtitre plates with a microtitre plate reader (Molecular Devices), as described in [83]. All assays were done in triplicate and were repeated independently at least twice. All Miller Unit values shown are from a single representative experiment and represent averages of triplicate measurements. Fold-change values were calculated by normalizing to the highest relevant empty vector control.

### Assessment of toxicity

For *E*. *coli* spot dilution assays, strain BW27785 (or the appropriate derivative) was transformed with the appropriate plasmid(s). Transformants were selected either on LB supplemented with appropriate antibiotic at 30°C or 37°C, or, for assays done with DH118 strains, on M9 maltose (0.2% maltose, 0.2% casamino acids, 1 mM MgSO_4_) plates supplemented with 250 μM IPTG at 30°C. Overnight cultures (grown in either LB or M9 maltose + IPTG) were back diluted in fresh medium + antibiotics to a starting OD600 of 0.03–0.05 and grown at the indicated temperature until cultures reached an OD600 of 0.5–1. Cultures were normalized by OD600 value and 1:10 serial dilutions were made in fresh LB or sterile phosphate buffered saline (PBS) in a microtitre plate. 5 μL of each culture were spotted on LB plates containing the appropriate antibiotics with or without the indicated level of inducer (IPTG, arabinose, or ATC). See figure legends for details on each experiment.

For *B*. *subtilis* spot dilution analysis (**Fig 5C**), relevant strains were streaked from glycerol stocks onto LB (Spec) agar and incubated at 37°C overnight followed by additional overnight incubation at RT. LB cultures were inoculated with single colonies, which were grown at 37°C until they reached an OD600 ~1. Cultures were normalized by OD600 value, and 1:10 serial dilutions were made in fresh LB in a microtitre plate. 5 μL of each dilution were spotted on LB agar supplemented with 100 μg/mL spectinomycin and LB agar supplemented with 100 μg/mL spectinomycin and 1 mM IPTG. Plates were incubated overnight at 37°C. Spot dilution analysis was done on both LB Miller (1% NaCl) and LB Lennox (0.5%) agar with identical results.

For *B*. *subtilis* growth curves, 5 mL LB cultures (no antibiotics) were inoculated with single colonies of relevant strains. Several dilutions of these cultures were made and grown with shaking at RT overnight. The next day, cultures that were in early to mid-log phase were back diluted to a starting OD600 of 0.01 in 5 mL fresh LB medium supplemented with 1 mM IPTG. Growth was monitored every 30–60 min by transferring 200 μL to a sterile microtitre plate and taking OD600 measurements on a plate reader (Molecular Devices). All strains were grown in triplicate, and growth curve experiments were repeated independently several times. Growth curve cultures were imaged at the indicated times using the same microscopy protocol as described below.

### Microscopic observation of cell morphology

Cultures used for microscopy were handled as described in the corresponding figure legends. Briefly, BW27785, DH73, NP1, or TB28 attHKHC488 cells were transformed with the relevant plasmids and transformants were selected for on LB containing the appropriate antibiotic(s). FB30 or DH118 transformants were selected for on M9 maltose supplemented with the appropriate antibiotic(s) and inducer. For most experiments, all growth incubation steps were done at 30°C. LB or M9 overnight cultures were back diluted to a starting OD600 of 0.02–0.05, grown without induction for 1 h (cultures had reached an OD600 of ~0.1–0.2), and then induced for toxin expression with the addition of IPTG (50, 100, or 200 μM), arabinose (0.2%), or ATC (10, 15, or 25 ng/mL). Cultures were typically in an OD600 range of 0.4–1 at the time of imaging.

For all snapshot images, cells were mounted on 2% agarose pads containing PBS, and microscopic observation was performed using an Olympus BX61 microscope (objective UplanF1 100x). Images were captured with a monochrome CoolSnapHQ digital camera (Photometrics) using Metamorph software version 6.1 (Universal Imaging). Cropping and minimal adjustment was performed with ImageJ [65] or Adobe Photoshop. Cell roundness quantification was done manually in ImageJ with the ObjectJ plugin. Briefly, the length of the cell was measured along the long axis, and the width was measured as the axis roughly perpendicular to the long axis. Angle measurements were spot-checked to ensure the axes intersected at an angle close to 90°. Cell roundness data were compiled from three independent experiments; 200–300 cells of each strain from each independent experiment were measured.

For time-lapse imaging of CbtA-induced morphology changes, pMT136, pMT139, and pMT146 were individually transformed into BW27785 cells. Overnight cultures were back diluted to a starting OD600 of 0.05 and grown at 30°C for 1 h without induction. Cells were concentrated 5x, and 2 μL were spotted on the bottom of a glass-bottomed dish (Willco dish HBSt-5040; Willco Wells). A 2% agarose pad containing LB growth medium supplemented with 100 μM IPTG was placed on top of the culture aliquot. Cells were imaged on a Nikon Ti inverted microscope using Nikon Elements software, a Photometrics CoolSNAP HQ2 Interline CCD camera, and a Well Plate Holder stage (TI-SH-W; Nikon) equipped with a humid, temperature-controlled incubator (TC-MIS; Bioscience Tools). The objective was heated to ~30°C using a Bioptechs objective heater system. Images were acquired every 3 min for 3 h. Image analysis was performed in FIJI and ImageJ.

### Western blot analysis

To compare levels of His_6_-CbtA-GFP, MreB, or FtsZ variants in *E*. *coli* cells, 2 mL of each culture grown as described in the relevant figure legends were pelleted and resuspended in various amounts of BugBuster lysis buffer (EMD) to normalize by OD600 value (OD600 of 1 = 100 μL lysis buffer). rlysozyme (3 kU; EMD) and Omnicleave (20U; Epicentre) were added to equal volumes of cell suspensions for a typical final concentration of 60U/μL and 0.4U/μL, respectively. Cells were lysed at room temperature for 30 min. Total protein concentration was measured by Bradford assay, and lysate volumes were adjusted using lysis buffer such that all samples contained equivalent amounts of protein. To compare levels of His_6_-CbtA-GFP and His_6_-CbtA-F65S- GFP in *B*. *subtilis* cells, Western blot analysis was performed on whole-cell lysates generated from growth curve cultures. Briefly, 2 mL of each *B*. *subtilis* culture were pelleted and resuspended in various amounts of lysis buffer (20 mM Tris-HCl pH 7.5, 50 mM EDTA, 100 mM NaCl) to normalize by OD600 value (OD600 of 1 = 100 μL lysis buffer). rlysozyme (30kU) and Omnicleave (20U) were added to cell suspensions, which were lysed for 30 min at 37°C.

All lysates were diluted 1:2 in 2x Laemmli buffer + BME (final concentration 1%) and boiled for 10 min; further dilutions were made in 1x Laemmli buffer + BME. Duplicate 10–20% Tris-glycine gels (Thermo Fisher) were run in MOPS-SDS buffer and transferred to nitrocellulose membranes using a wet transfer system (Life Technologies). Membranes were incubated with primary antibodies α-GFP 1:5,000 (Roche), α-RpoA 1:10,000 (Neoclone), α-FtsZ 1:10,000 (T. Bernhardt), α-MreB 1:5,000 (T. Bernhardt), α-SigA 1:5,000 (D. Rudner), or α-Spo0J 1:5,000 (D. Rudner) and HRP-conjugated α-mouse or α-rabbit secondary antibodies (Cell Signaling). Chemiluminescent signal was detected using ECL Plus reagent (GE Healthcare) on a ChemiDock XRS+ system (Bio-Rad).

## Supporting information

S1 Text Supplemental methods and results. (PDF)

S1 Fig Characterization of toxin mutants. (A) Cell morphology phenotypes. Cells of strain BW27785 producing His_6_-CbtA-GFP or His_6_-CbtA-F65S-GFP from the IPTG-inducible promoter *pT5-lac* on multi-copy plasmid pMT139 or pMT146, respectively, were imaged every 3 min for 3 h on 2% agarose pads containing LB and 100 μM IPTG at 30°C. Scale bars represent 5 μm. (B) Spot dilution assay showing toxicity of CbtA-F65S, YpjF-F65S, and YkfI-F65S variants. Late log cultures of BW27785/pMT136 (*his*_*6*_*-gfp*), BW27785/pMT144 (*his*_*6*_*-ykfI-F65S-gfp*), BW27785/pMT146 (*his*_*6*_*-cbtA-F65S-gfp*), and BW27785/pMT188 (*his*_*6*_*-ypjF-F65S-gfp*) grown without induction, were spotted on LB (Cm) plates with or without 100 μM IPTG; plates were incubated at 37°C overnight. Dilutions 10^0^ to 10^−4^ are shown. (C) Western blot analysis using a GFP antibody (Roche) to detect His_6_-CbtA-GFP levels indicates that the CbtA single and double mutants are produced at similar levels as the wild-type fusion protein. Cells were harvested and lysed after 2 h induction with 50 μM IPTG at 30°C. Several dilutions of cell lysates (1:2, 1:4, and 1:10) are shown. RpoA from the same samples was detected on a separate blot using an antibody that specifically binds the C-terminal domain (Neoclone); this serves as a loading control. (D) Effects of CbtA variants on ZapA-GFP localization. Strain NP1 (TB28 *zapA-gfp*) [50] was transformed with pSG360 (empty vector), pDH325 (*placUV5*-*cbtA)*, pDH327 (*placUV5*-*cbtA-R15C)*, or pDH328 (*placUV5*-*cbtA-R15C/F65S)*. Overnight cultures were back diluted to a starting OD600 of 0.03 in fresh LB (SpecStrep), grown for 1 h at 30°C, then induced for toxin expression with 200 μM IPTG for 2 h at 30°C. Cultures were in mid-log phase at the time of imaging. E) Effects of CbtA variants on GFP-FtsZ localization. Strain TB28 attHKHC488 (*plac-sfgfp-ftsZ*), which directs the production of ectopic GFP-FtsZ in an IPTG-inducible manner, was transformed with the same vectors as in (D). Cultures were grown and imaged as in (D). For all microscopy panels, scale bar represents 5 μm.
(TIF)

S2 Fig Slower growth rescues *ftsZ-L169P* growth defect. (A) MreB and FtsZ do not interact detectably in the context of a transcription-based bacterial two-hybrid assay. Results of a β-galactosidase assay performed with reporter strain cells containing compatible plasmids encoding the indicated λCI fusion protein and the indicated α fusion protein are shown. Cells were grown in the presence of 100 μM IPTG. Positive control self-interactions between α-MreB and λCI-MreB and between α-FtsZ and λCI-FtsZ are also shown. Bars represent the average of triplicate values; error bars represent standard deviation. Dashed line designates highest basal *lacZ* expression, i.e. the Miller Unit value of the highest empty vector control. (B) The ability of *ftsZ* H6/H7 loop mutant alleles to complement growth of the CH45/pDB346 depletion strain was measured by spot dilution assay. Briefly, overnight cultures of CH45/pDB346 strains transformed with pBRα (empty vector), pDR3 (wt *ftsZ*), pDH27 (*ftsZ-D180N*), pDH28 (*ftsZ-S177P*), pDH29 (*ftsZ-F182S*), or pDH30 (*ftsZ-L169P)* were back diluted to an OD600 of 0.05 in LB and grown at 37°C until they reached an OD600 of 1–1.5. Cultures were normalized to OD600, serially diluted in fresh LB, and spotted onto LB plates supplemented with the appropriate antibiotics, with or without 100 μM IPTG. Plates were incubated at the indicated temperature overnight. Dilutions 10^−1^ to 10^−5^ are shown. (C) Cell morphology phenotype of *ftsZ-L169P* strain in various growth conditions. An overnight culture of DH73 (BW27785 *ftsZ-L169P*) grown in M9 maltose (1 mM MgSO_4_, 0.4% maltose, 0.01% casamino acids) was back diluted in either LB or M9 maltose and grown at the indicated temperature until the culture reached mid-log phase. Phase contrast images are shown; scale bars represent 5 μm. (D) Cell length measurements for strains wt *ftsZ* and *ftsZ-L169P* grown in M9 maltose at 30°C. Overnight M9 maltose (0.4% maltose, 0.01% casamino acids, 1 mM MgSO_4_) cultures of either BW27785 or DH73 were back diluted into the same medium and grown at 30°C until cultures reached an OD600 of ~0.3. Phase contrast images are shown; scale bars represent 5 μm. Cell length measurements were made manually in ImageJ using the ObjectJ plugin (*n* = 160 for BW27785; *n* = 156 for DH73). Values are reported as average ± standard deviation. (E) Western blot analysis using an FtsZ-specific antibody shows that wild-type FtsZ and FtsZ-L169P accumulate to comparable levels. Cell lysates were prepared from mid-log cultures of strains BW27785 and DH73 grown as described in (C). Several dilutions of cell lysates (1:2, 1:4, and 1:10) are shown. The full-length FtsZ 40 kDa band is indicated. RpoA from the same samples was detected on a separate blot and serves as a loading control.
(TIF)

S3 Fig CbtA production in *Bsu ftsZ (loop*^*Eco*^*)* strain leads to increased lysis. (A) CbtA production has no effect on cell morphology of *B*. *subtilis* strains containing the wt *ftsZ* allele. *Bsu* strains with or without *spec* linked to the endogenous *ftsZ* locus and producing His_6_-GFP or His_6_-CbtA-GFP from *pHYPERSPANK* at the *ycgO* locus were imaged. From left to right, phase contrast and GFP fluorescence images of strains DH84, DH85, DH100, and DH101 are shown. Briefly, overnight cultures grown in LB at 22°C were back diluted to a starting OD600 of 0.01 in LB supplemented with 1 mM IPTG. Cultures were grown at 37°C for 1.5 hrs until cultures reached an OD600 ~0.2. The cell length of ~200 cells was measured for each strain (*n* = 200, *n* = 200, *n* = 203, *n* = 206, from left to right). Measurements from a single representative experiment are shown. (B) *Bsu* strains bearing the *ftsZ (loop*^*Eco*^*)* chimeric allele display cell division defects. Strain DH102 with the *ftsZ (loop*^*Eco*^*)* allele linked to *spec* and producing His_6-_GFP from *pHYPERSPANK* at the *ycgO* locus was imaged as described in (A). GFP fluorescence images are shown. The mini-cells and abnormal septa indicated by the yellow arrows are shown in the zoomed-in panels. (C) CbtA causes cell lysis in *ftsZ (loop*^*Eco*^*)* strain. Shown are phase contrast (left) and GFP fluorescence images (right) of strains DH102, DH103, and DH106 (from top to bottom) containing the chimeric *ftsZ (loop*^*Eco*^*)* allele linked to *spec* and producing His_6_-GFP, His_6_-CbtA- GFP, or His_6_-CbtA-F65S-GFP from *pHYPERSPANK* at the *ycgO* locus, respectively. Cultures were grown as described in (A) and imaged after 200 min at 37°C when DH102 and DH106 were at an OD600 ~1.2, and DH103 had reached an OD600 of only ~0.5. In all microscopy images (A-C), scale bars represent 5 μm. (D) His_6_-CbtA-GFP and His_6_-CbtA-F65S-GFP levels from a growth curve experiment similar to that described in **Fig 5D** were assayed by Western blot, in triplicate, using an anti-His_6_ antibody (Genscript). Lysates were generated from triplicate cultures of strains DH101, DH103, and DH106 (left to right) after 4 h growth. SigA and Spo0J levels were also measured in the same samples and serve as loading controls.
(TIF)

S4 Fig Characterization of MreB mutants. Two-hybrid interactions of wild-type α-MreB and α-MreB-E319K (RodZ-interface mutant) with λCI-CbtA and λCI-RodZ_NTD_ (residues 2–84) are shown in (A), and the interactions of α-MreB-S269F with wild-type and mutant λCI-CbtA are shown in (B). Reporter strain cells containing compatible plasmids encoding the indicated λCI-CbtA variant, λCI-RodZ_NTD_, or λCI and the indicated α-MreB variant or wild-type α were grown in the presence of 100 μM IPTG and assayed for β-galactosidase. Bars represent the average of triplicate values; error bars represent standard deviation. Dashed line designates highest basal *lacZ* expression, i.e. the Miller Unit value of the highest empty vector control. (C) *mreB*, *mreB-E262G*, and *mreB-S269F* strains exhibit comparable growth rates. Growth curve analysis was performed on strains DH118/pFB149 (*mreB*), DH118/pDH278 (*mreB-E262G*), and DH118/pDH332 (*mreB-S269F*). Four replicate cultures of each strain were grown in LB supplemented with 250 μM IPTG at 37°C over several hours. Each point represents the average of four replicate values; error bars represent standard deviation. Note that the symbols for the wild-type *mreB* strain are not visible because they are hidden by the symbols for the *mreB-S269F* strain. (D) Western blot analysis of MreB variants. Cultures of DH118/pFB149 and DH118/pDH278 (*mreB-E262G*) (top blot) were grown in LB + 250 μM IPTG at 30°C, and cultures of DH118/pFB149 and DH118/pDH332 (bottom blot) were grown in LB + 250 μM IPTG at 37°C. Cells were harvested and lysed after reaching mid-log phase. MreB, MreB-E262G, and MreB-S269F levels were assayed by Western blot analysis using anti-serum specific to MreB. Several dilutions of cell lysates are shown (1:2, 1:4, 1:8 for the top blot; 1:2, 1:8, 1:16 for the bottom blot). RpoA from the same samples was detected on separate blots and serves as a loading control. (E) Effect of MreB substitution S269F on cell morphology phenotypes in the presence or absence of overproduced CbtA-F65S. Phase contrast images of DH118/pFB149 (WT *mreB*) and DH118/pDH332 (*mreB-S269F*) transformed with either the pSG369 empty vector or pDH335 (*ptet-cbtA-F65S*) are shown. Overnight cultures grown in M9 maltose (1 mM MgSO_4_, 0.2% maltose, 0.2% casamino acids) + 250 μM IPTG at 30°C were back diluted to a starting OD600 of 0.03 in LB (CarbSpecStrep) supplemented with 250 μM IPTG, and grown for 1 h at either 30°C or 37°C. Cultures were induced by addition of either 10,15, or 25 ng/mL ATC and grown for an additional hour at the indicated temperature, at which point microscopic analysis was performed. Scale bars represent 5 μm.
(TIF)

S5 Fig High sequence conservation of the CbtA family of toxins. (A) Shown is a multiple sequence alignment of CbtA, YpjF, and YkfI amino acid sequences from *E*. *coli* K12 (Ecocyc). YpjF and YkfI are 78% identical; CbtA is 58% identical to YkfI and 62% identical to YpjF. The alignment was made using TCoffee, and the figure was prepared in Boxshade. (B) Two-hybrid interactions of λCI-YpjF-C15R and λCI-YkfI-C15R with α-MreB. Reporter strain cells containing compatible plasmids encoding the indicated λCI toxin fusion variant and α-MreB (or wild-type α) were grown in the presence of 100 μM IPTG and assayed for β-galactosidase. Bars represent the average of triplicate values; error bars represent standard deviation. Dashed line designates highest basal *lacZ* expression, i.e. the Miller Unit value of the highest empty vector control.
(TIF)

S1 Table Summary of two-hybrid interactions of α-MreB double protofilament interface mutants. Two-hybrid interactions of α-MreB variants bearing the indicated substitutions with λCI-CbtA and λCI-RodZ_NTD_ (residues 2–84) are shown. Reporter strain cells containing compatible plasmids encoding the indicated α-MreB variant or wild-type α and either λCI-CbtA or λCI-RodZ_NTD_ were grown in the presence of 100 μM IPTG (for λCI-CbtA interaction) or 25 μM IPTG (for λCI-RodZ_NTD_ interaction) and assayed in triplicate for β-galactosidase. Average Miller Unit values were calculated from biological triplicates from a single representative experiment. Fold-change values were calculated by dividing the average Miller Unit value of the strain producing both fusion proteins of interest (e.g. α-MreB-K77D + λCI-CbtA) by the relevant empty vector control average Miller Unit value (either α + λCI-CbtA or α + λCI-RodZ_NTD_). % wild-type interaction was calculated by dividing the fold-change value of the relevant mutant interaction by the fold-change value of the wild-type interaction and multiplying by 100.
(PDF)

S2 Table Bacterial strains used in this study. (PDF)

S3 Table Plasmids used in this study. (PDF)

S4 Table Oligonucleotides used in this study. (PDF)
