# Supplementary material for: CbtA toxin of Escherichia coli inhibits cell division and cell elongation via direct and independent interactions with FtsZ and MreB
Source: PLoS Genet. 2017 Sep 20;13(9):e1007007. doi: 10.1371/journal.pgen.1007007 (PMC5624674; doi:10.1371/journal.pgen.1007007)
Supplement: S1 Text — (PDF) [file pgen.1007007.s001.pdf]

S1 Text. Supplemental methods and results.

Isolation of the CbtA-F65S variant

Prior to the report of CbtA being a cytoskeletal inhibitor [1], we had sought to isolate reduced-toxicity variants of CbtA and its two homologs to use as tools to probe their mechanism of toxicity. A dual selection/screen strategy was employed in order to identify variants with reduced toxicity. The *cbtA*, *ypjF,* and *ykfI* genes were amplified by error-prone PCR using *Taq* polymerase and oligos oSG625/oSG626, digested with SfiI, and cloned into the SfiI-digested pCA24N vectors pMT139, pMT138, and p3-37. The mutagenized alleles, which were expressed under the control of the *pT5-lac* IPTG-inducible promoter, all contained an N-terminal His_6 tag and were fused to GFP at the C-terminus. These mutant libraries were transformed into *E. coli* BW27785, plated on LB medium supplemented with a high concentration of IPTG (500 µM), and incubated overnight at 37 ºC, thus selecting for toxin variants with lowered toxicity. In order to eliminate de-stabilized variants, these surviving colonies were patched onto the same medium, and the next day, were screened for GFP-fluorescence using a Typhoon fluorescence scanner (GE) as a first test to determine whether or not the GFP-fusion protein was still being produced to roughly comparable levels. The stability of isolated variants was further assessed by Western blot analysis with a GFP-specific monoclonal antibody (Roche). Although many other selected variants were found to have decreased stability as compared to the wild-type proteins, a stable YpjF variant, YpjF-F65S, was isolated. The F65 residue is conserved in all three toxins (**S5A Fig**). When the F65S substitution was introduced into CbtA and YkfI, the resulting variants were still toxic upon overproduction, as was the YpjF-F65S variant, though to a slightly lesser degree (**S1B Fig**).

ftsZ-L169P, *ftsZ-D180N*, and *ftsZ-S177P* complement growth of an *ftsZ* complementation strain

The complementation phenotypes of the H6/H7 loop mutant alleles identified in our two-hybrid screen were tested using an *ftsZ* depletion strain (CH45/pDB346). Briefly, this depletion strain harbors an *ftsZ*^{0} allele on the chromosome, which is complemented by a plasmid-encoded

wild-type allele under the control of the promoter λP_R and the temperature sensitive repressor λCI857 [2]. At the non-permissive temperature (≥ 37 ºC), λCI857 is destabilized and wild-type *ftsZ* is expressed. When cells are shifted to the permissive temperature (30 ºC), λCI857 is properly folded and able to repress *ftsZ* expression from λP_R.

To assess complementation, our identified H6/H7 loop mutant alleles were cloned into the pDR3 plasmid under control of *plac* and transformed into the CH45/pDB346 depletion strain. We were not able to successfully clone *ftsZ-L179R* into the pDR3 plasmid; thus, it was excluded from this analysis. When cells were plated on LB without IPTG at 37 ºC, all strains grew well (**S2B Fig**); when cells were plated on LB with 100 µM IPTG at 30 ºC, the *ftsZ-L169P*, *ftsZ-D180N*, and *ftsZ-S177P* strains grew as well as the strain expressing wild-type *ftsZ. ftsZ-F182S* did not support growth (**S2B Fig**). Only *ftsZ-L169P* could be integrated into the chromosome at the endogenous *ftsZ* locus. The media and temperature-dependent growth phenotypes described in the main text are shown in **S2C Fig**.

Microscopic observation of *B. subtilis* strains

IPTG-inducible production of His_6-CbtA-GFP from the *ycgO* locus in *B. subtilis* (strain DH85) had no appreciable effect on cell length or cell morphology as compared to an isogenic strain producing His_6-GFP (strain DH84) (**S3A Fig**). With the introduction of a genetically linked spectinomycin resistance cassette (*spec*) immediately downstream of the *ftsZ* ORF, cell length increased (**S3A Fig**). This may be due to alteration of normal transcription termination perhaps leading to a decrease in FtsZ levels, though this was not tested here. Importantly, overproduction of His_6-CbtA-GFP in this background did not lead to further cell elongation or any other noticeable morphological change (**S3A Fig**). This observation is consistent with the lack of toxicity observed in **Fig 5C and D**.

Introduction of the chimeric *ftsZ(loop^{Eco})* allele resulted in multiple division abnormalities (**S3B,C Fig**). All strains harboring this allele (DH102, DH103, and DH106) exhibited heterogeneous cell length, with a fraction of cells forming very long filaments. Careful quantification of cell length was very difficult as many cells were curved or even in some cases

formed spirals, and therefore was not performed. Additionally, mini-cells, pre-mini-cell structures, and septa with abnormal appearance were observed frequently (**S3B,C Fig**). Thus, this chimeric allele is not able to fully support normal cell division. The most obvious phenotype observed upon production of His_6-CbtA-GFP in this background, was pronounced lysis (**S3C Fig**). After induction of expression for 200 min, strain DH103 (His_6-CbtA-GFP*)* showed signs of copious lysis, while strains DH102 (His_6-GFP*)* and DH106 (His_6-CbtA-F65S-GFP) did not. This phenotype correlates with the cell growth defect observed in liquid culture (**Fig 5D**). In all strains imaged, His_6-CbtA-GFP displayed diffuse localization.

References

1. Tan Q, Awano N, Inouye M. YeeV is an *Escherichia coli* toxin that inhibits cell division by targeting the cytoskeleton proteins, FtsZ and MreB. Mol Microbiol. 2010;79(1):109–18. pmid: 21166897.

2. Raskin DM, de Boer PA. The MinE ring: an FtsZ-independent cell structure required for selection of the correct division site in *E. coli*. Cell. 1997;91(5):685–94. pmid: 9393861.
