# Supplementary figures and images for: CbtA toxin of Escherichia coli inhibits cell division and cell elongation via direct and independent interactions with FtsZ and MreB
Source: PLoS Genet. 2017 Sep 20;13(9):e1007007. doi: 10.1371/journal.pgen.1007007 (PMC5624674; doi:10.1371/journal.pgen.1007007)

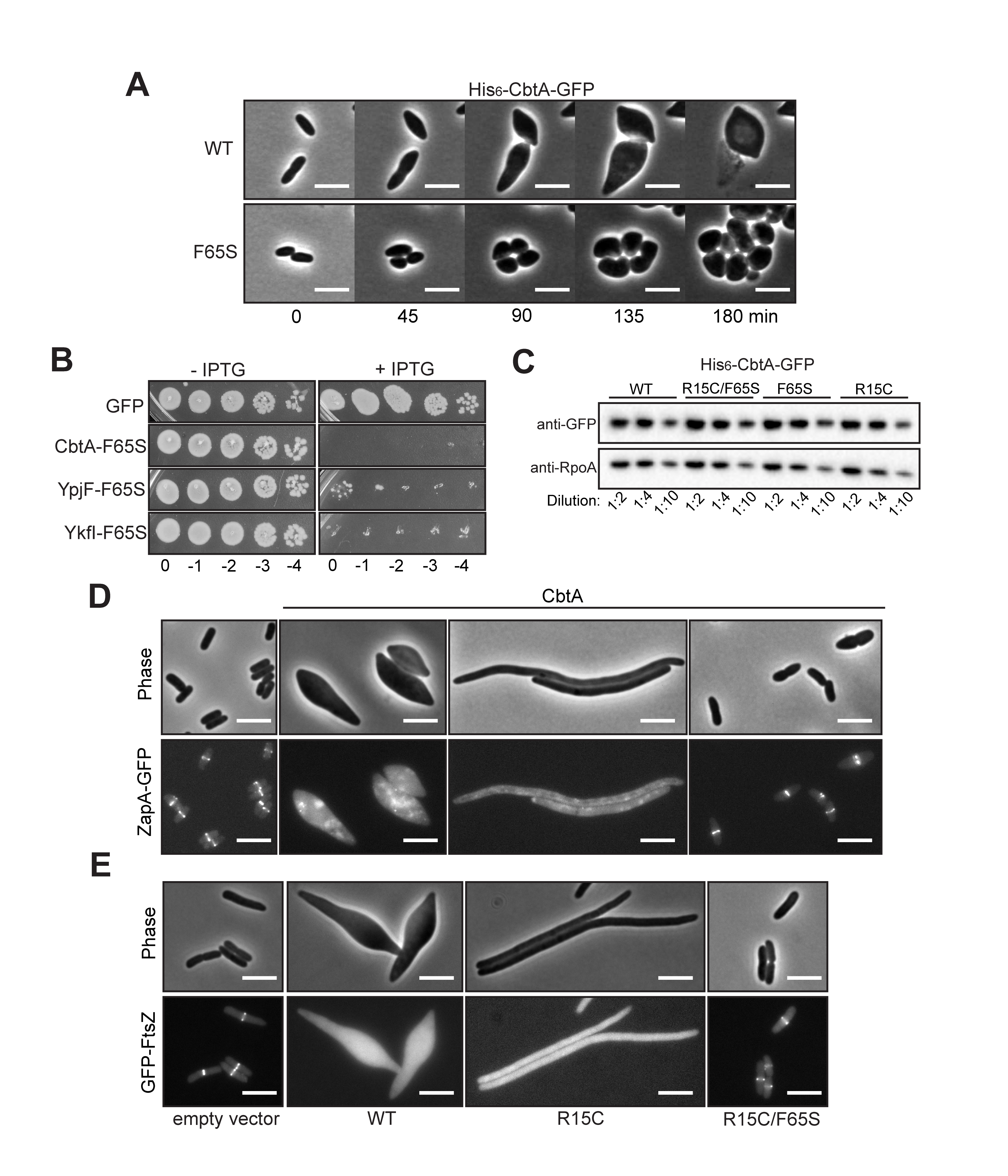

Supplement: S1 Fig — (A) Cell morphology phenotypes. Cells of strain BW27785 producing His6-CbtA-GFP or His6-CbtA-F65S-GFP from the IPTG-inducible promoter pT5-lac on multi-copy plasmid pMT139 or pMT146, respectively, were imaged every 3 min for 3 h on 2% agarose pads containing LB and 100 μM IPTG at 30°C. Scale bars represent 5 μm. (B) Spot dilution assay showing toxicity of CbtA-F65S, YpjF-F65S, and YkfI-F65S variants. Late log cultures of BW27785/pMT136 (his6-gfp), BW27785/pMT144 (his6-ykfI-F65S-gfp), BW27785/pMT146 (his6-cbtA-F65S-gfp), and BW27785/pMT188 (his6-ypjF-F65S-gfp) grown without induction, were spotted on LB (Cm) plates with or without 100 μM IPTG; plates were incubated at 37°C overnight. Dilutions 100 to 10−4 are shown. (C) Western blot analysis using a GFP antibody (Roche) to detect His6-CbtA-GFP levels indicates that the CbtA single and double mutants are produced at similar levels as the wild-type fusion protein. Cells were harvested and lysed after 2 h induction with 50 μM IPTG at 30°C. Several dilutions of cell lysates (1:2, 1:4, and 1:10) are shown. RpoA from the same samples was detected on a separate blot using an antibody that specifically binds the C-terminal domain (Neoclone); this serves as a loading control. (D) Effects of CbtA variants on ZapA-GFP localization. Strain NP1 (TB28 zapA-gfp) [50] was transformed with pSG360 (empty vector), pDH325 (placUV5-cbtA), pDH327 (placUV5-cbtA-R15C), or pDH328 (placUV5-cbtA-R15C/F65S). Overnight cultures were back diluted to a starting OD600 of 0.03 in fresh LB (SpecStrep), grown for 1 h at 30°C, then induced for toxin expression with 200 μM IPTG for 2 h at 30°C. Cultures were in mid-log phase at the time of imaging. E) Effects of CbtA variants on GFP-FtsZ localization. Strain TB28 attHKHC488 (plac-sfgfp-ftsZ), which directs the production of ectopic GFP-FtsZ in an IPTG-inducible manner, was transformed with the same vectors as in (D). Cultures were grown and imaged as in (D). For all microscopy panels, scale bar represen [file pgen.1007007.s002.tif]

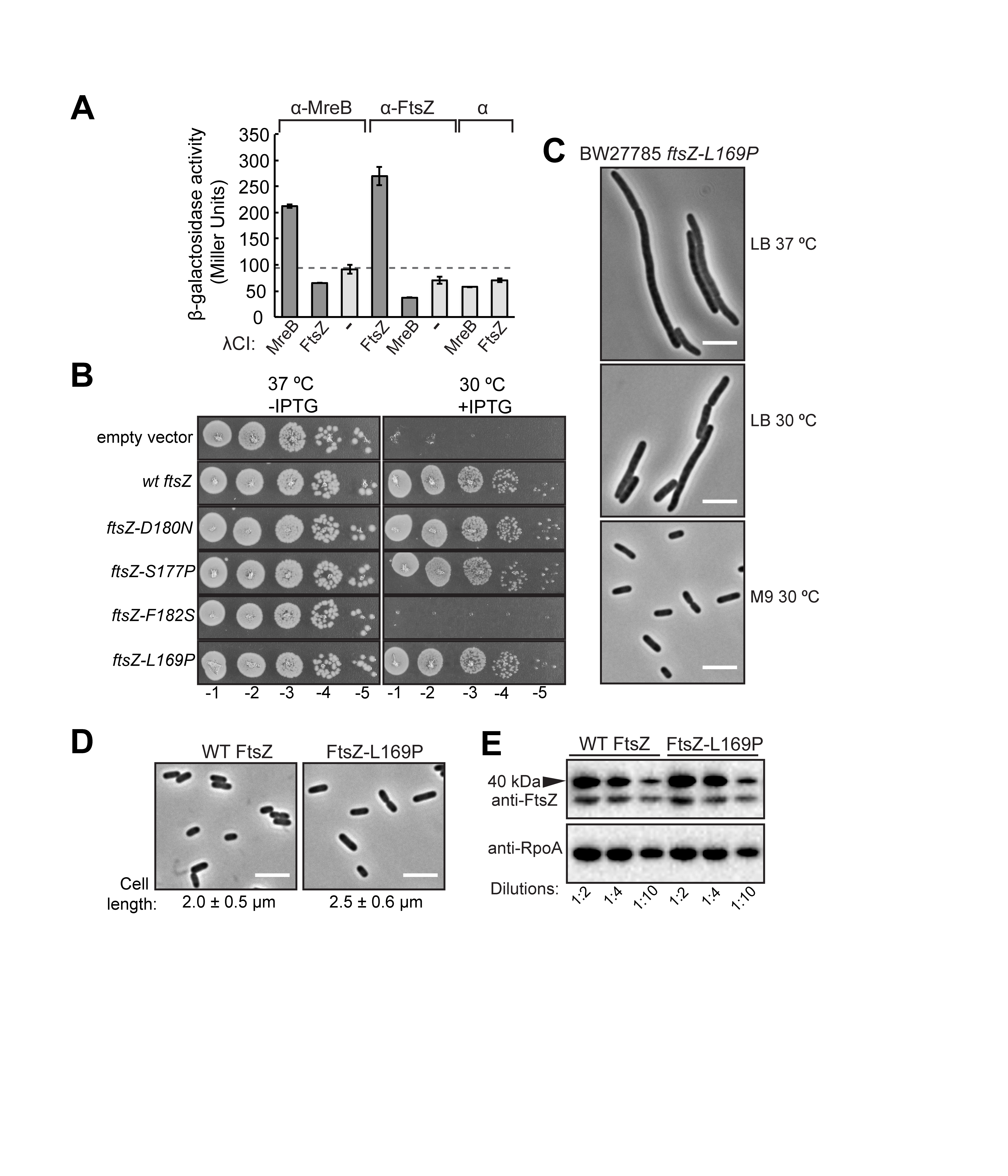

Supplement: S2 Fig — (A) MreB and FtsZ do not interact detectably in the context of a transcription-based bacterial two-hybrid assay. Results of a β-galactosidase assay performed with reporter strain cells containing compatible plasmids encoding the indicated λCI fusion protein and the indicated α fusion protein are shown. Cells were grown in the presence of 100 μM IPTG. Positive control self-interactions between α-MreB and λCI-MreB and between α-FtsZ and λCI-FtsZ are also shown. Bars represent the average of triplicate values; error bars represent standard deviation. Dashed line designates highest basal lacZ expression, i.e. the Miller Unit value of the highest empty vector control. (B) The ability of ftsZ H6/H7 loop mutant alleles to complement growth of the CH45/pDB346 depletion strain was measured by spot dilution assay. Briefly, overnight cultures of CH45/pDB346 strains transformed with pBRα (empty vector), pDR3 (wt ftsZ), pDH27 (ftsZ-D180N), pDH28 (ftsZ-S177P), pDH29 (ftsZ-F182S), or pDH30 (ftsZ-L169P) were back diluted to an OD600 of 0.05 in LB and grown at 37°C until they reached an OD600 of 1–1.5. Cultures were normalized to OD600, serially diluted in fresh LB, and spotted onto LB plates supplemented with the appropriate antibiotics, with or without 100 μM IPTG. Plates were incubated at the indicated temperature overnight. Dilutions 10−1 to 10−5 are shown. (C) Cell morphology phenotype of ftsZ-L169P strain in various growth conditions. An overnight culture of DH73 (BW27785 ftsZ-L169P) grown in M9 maltose (1 mM MgSO4, 0.4% maltose, 0.01% casamino acids) was back diluted in either LB or M9 maltose and grown at the indicated temperature until the culture reached mid-log phase. Phase contrast images are shown; scale bars represent 5 μm. (D) Cell length measurements for strains wt ftsZ and ftsZ-L169P grown in M9 maltose at 30°C. Overnight M9 maltose (0.4% maltose, 0.01% casamino acids, 1 mM MgSO4) cultures of either BW27785 or DH73 were back diluted into the same medium and grown at [file pgen.1007007.s003.tif]

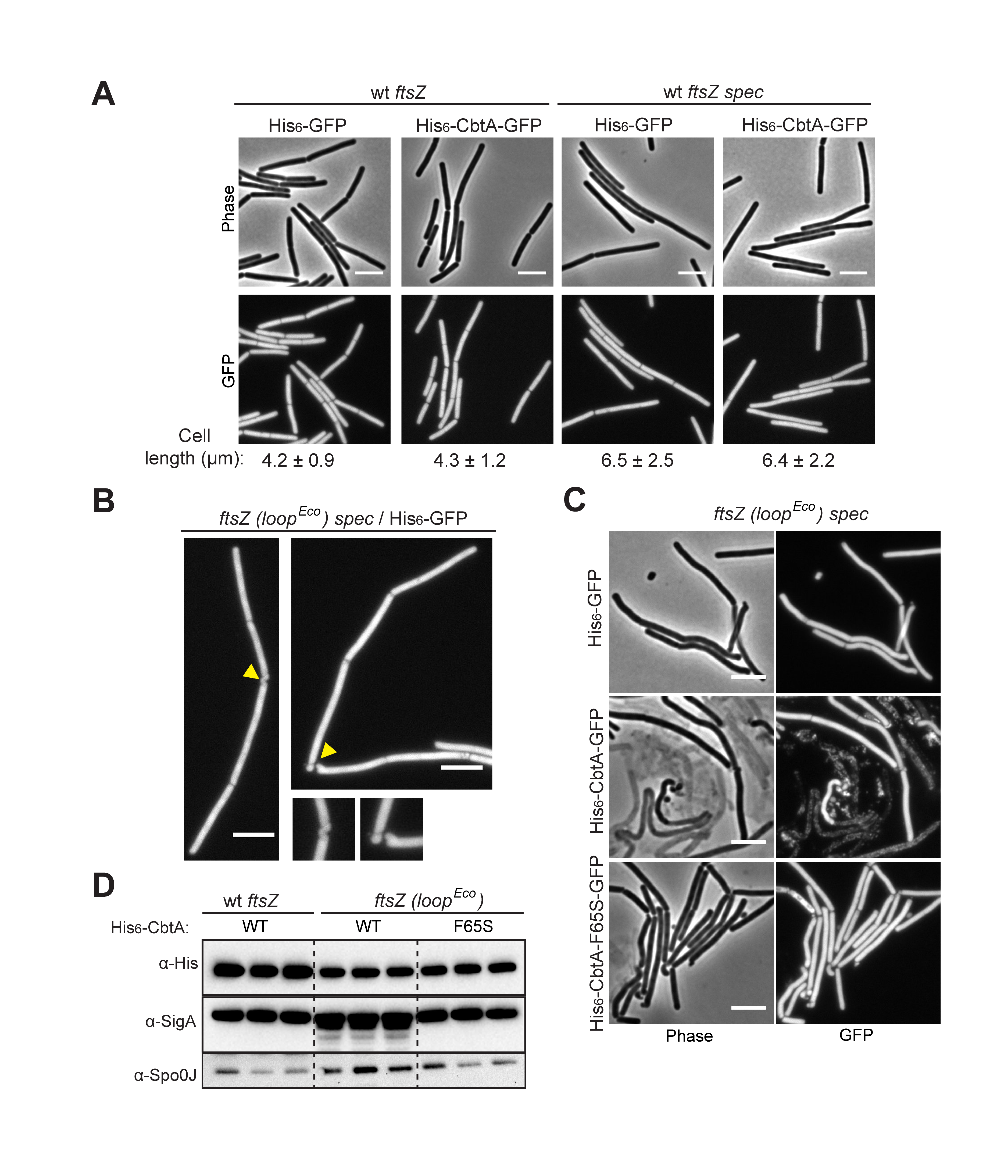

Supplement: S3 Fig — (A) CbtA production has no effect on cell morphology of B. subtilis strains containing the wt ftsZ allele. Bsu strains with or without spec linked to the endogenous ftsZ locus and producing His6-GFP or His6-CbtA-GFP from pHYPERSPANK at the ycgO locus were imaged. From left to right, phase contrast and GFP fluorescence images of strains DH84, DH85, DH100, and DH101 are shown. Briefly, overnight cultures grown in LB at 22°C were back diluted to a starting OD600 of 0.01 in LB supplemented with 1 mM IPTG. Cultures were grown at 37°C for 1.5 hrs until cultures reached an OD600 ~0.2. The cell length of ~200 cells was measured for each strain (n = 200, n = 200, n = 203, n = 206, from left to right). Measurements from a single representative experiment are shown. (B) Bsu strains bearing the ftsZ (loopEco) chimeric allele display cell division defects. Strain DH102 with the ftsZ (loopEco) allele linked to spec and producing His6-GFP from pHYPERSPANK at the ycgO locus was imaged as described in (A). GFP fluorescence images are shown. The mini-cells and abnormal septa indicated by the yellow arrows are shown in the zoomed-in panels. (C) CbtA causes cell lysis in ftsZ (loopEco) strain. Shown are phase contrast (left) and GFP fluorescence images (right) of strains DH102, DH103, and DH106 (from top to bottom) containing the chimeric ftsZ (loopEco) allele linked to spec and producing His6-GFP, His6-CbtA- GFP, or His6-CbtA-F65S-GFP from pHYPERSPANK at the ycgO locus, respectively. Cultures were grown as described in (A) and imaged after 200 min at 37°C when DH102 and DH106 were at an OD600 ~1.2, and DH103 had reached an OD600 of only ~0.5. In all microscopy images (A-C), scale bars represent 5 μm. (D) His6-CbtA-GFP and His6-CbtA-F65S-GFP levels from a growth curve experiment similar to that described in Fig 5D were assayed by Western blot, in triplicate, using an anti-His6 antibody (Genscript). Lysates were generated from triplicate cultures of strains DH101, DH103, and DH106 (left [file pgen.1007007.s004.tif]

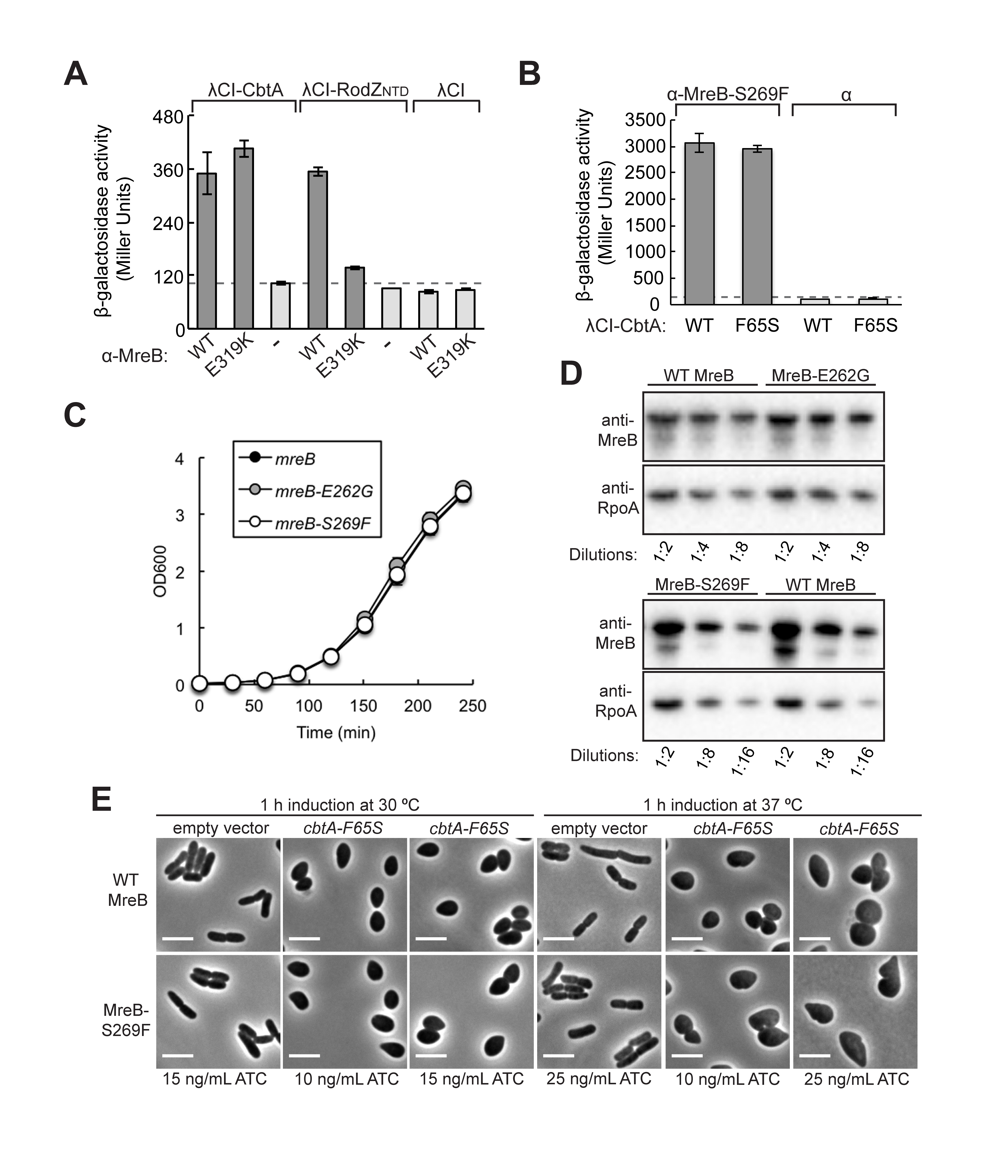

Supplement: S4 Fig — Two-hybrid interactions of wild-type α-MreB and α-MreB-E319K (RodZ-interface mutant) with λCI-CbtA and λCI-RodZNTD (residues 2–84) are shown in (A), and the interactions of α-MreB-S269F with wild-type and mutant λCI-CbtA are shown in (B). Reporter strain cells containing compatible plasmids encoding the indicated λCI-CbtA variant, λCI-RodZNTD, or λCI and the indicated α-MreB variant or wild-type α were grown in the presence of 100 μM IPTG and assayed for β-galactosidase. Bars represent the average of triplicate values; error bars represent standard deviation. Dashed line designates highest basal lacZ expression, i.e. the Miller Unit value of the highest empty vector control. (C) mreB, mreB-E262G, and mreB-S269F strains exhibit comparable growth rates. Growth curve analysis was performed on strains DH118/pFB149 (mreB), DH118/pDH278 (mreB-E262G), and DH118/pDH332 (mreB-S269F). Four replicate cultures of each strain were grown in LB supplemented with 250 μM IPTG at 37°C over several hours. Each point represents the average of four replicate values; error bars represent standard deviation. Note that the symbols for the wild-type mreB strain are not visible because they are hidden by the symbols for the mreB-S269F strain. (D) Western blot analysis of MreB variants. Cultures of DH118/pFB149 and DH118/pDH278 (mreB-E262G) (top blot) were grown in LB + 250 μM IPTG at 30°C, and cultures of DH118/pFB149 and DH118/pDH332 (bottom blot) were grown in LB + 250 μM IPTG at 37°C. Cells were harvested and lysed after reaching mid-log phase. MreB, MreB-E262G, and MreB-S269F levels were assayed by Western blot analysis using anti-serum specific to MreB. Several dilutions of cell lysates are shown (1:2, 1:4, 1:8 for the top blot; 1:2, 1:8, 1:16 for the bottom blot). RpoA from the same samples was detected on separate blots and serves as a loading control. (E) Effect of MreB substitution S269F on cell morphology phenotypes in the presence or absence of overproduced CbtA-F65S. Phase contra [file pgen.1007007.s005.tif]

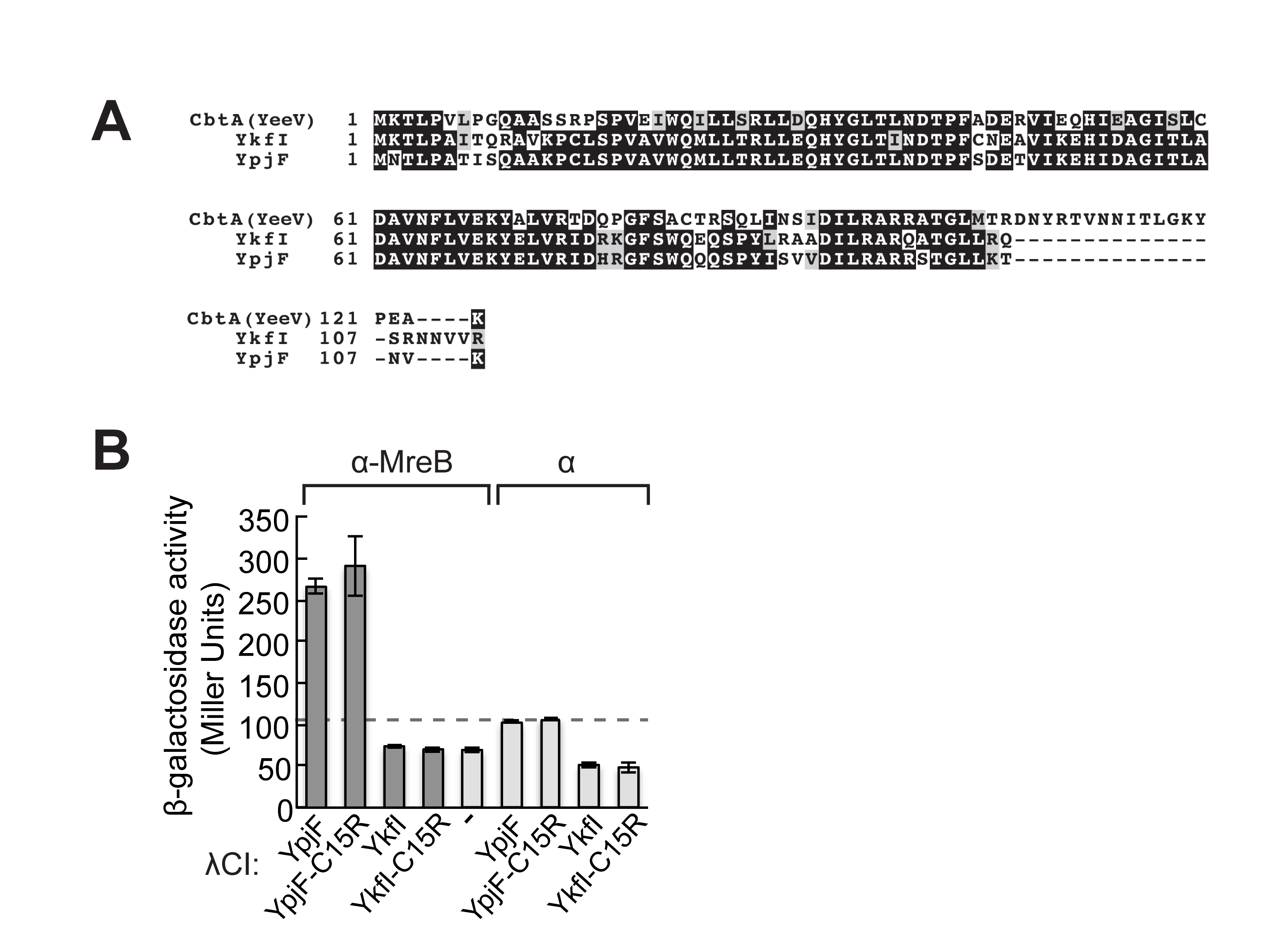

Supplement: S5 Fig — (A) Shown is a multiple sequence alignment of CbtA, YpjF, and YkfI amino acid sequences from E. coli K12 (Ecocyc). YpjF and YkfI are 78% identical; CbtA is 58% identical to YkfI and 62% identical to YpjF. The alignment was made using TCoffee, and the figure was prepared in Boxshade. (B) Two-hybrid interactions of λCI-YpjF-C15R and λCI-YkfI-C15R with α-MreB. Reporter strain cells containing compatible plasmids encoding the indicated λCI toxin fusion variant and α-MreB (or wild-type α) were grown in the presence of 100 μM IPTG and assayed for β-galactosidase. Bars represent the average of triplicate values; error bars represent standard deviation. Dashed line designates highest basal lacZ expression, i.e. the Miller Unit value of the highest empty vector control. (TIF) [file pgen.1007007.s006.tif]
