# Supplementary material for: CbtA toxin of Escherichia coli inhibits cell division and cell elongation via direct and independent interactions with FtsZ and MreB
Source: PLoS Genet. 2017 Sep 20;13(9):e1007007. doi: 10.1371/journal.pgen.1007007 (PMC5624674; doi:10.1371/journal.pgen.1007007)
Supplement: S1 Table — Two-hybrid interactions of α-MreB variants bearing the indicated substitutions with λCI-CbtA and λCI-RodZNTD (residues 2–84) are shown. Reporter strain cells containing compatible plasmids encoding the indicated α-MreB variant or wild-type α and either λCI-CbtA or λCI-RodZNTD were grown in the presence of 100 μM IPTG (for λCI-CbtA interaction) or 25 μM IPTG (for λCI-RodZNTD interaction) and assayed in triplicate for β-galactosidase. Average Miller Unit values were calculated from biological triplicates from a single representative experiment. Fold-change values were calculated by dividing the average Miller Unit value of the strain producing both fusion proteins of interest (e.g. α-MreB-K77D + λCI-CbtA) by the relevant empty vector control average Miller Unit value (either α + λCI-CbtA or α + λCI-RodZNTD). % wild-type interaction was calculated by dividing the fold-change value of the relevant mutant interaction by the fold-change value of the wild-type interaction and multiplying by 100. (PDF) [file pgen.1007007.s007.pdf]

S1 Table. Summary of two-hybrid interactions of α-MreB double-protofilament interface mutants.

α-MreB mutant	λCI-CbtA[✚]	λCI-RodZ$_{NTD}$[❖]
WT	+++	+++
K77D	++	++
D78K	++	+++
F84A	-	++
V121E	+++	+++
R124D	++	+++
A125D	++	++
I126V*	-	+++
R127D	+	-
E128K	++++	+++
V173A*	-	++`
R188D	++++	+++
D192K	-	++
E196G*	+	+++
E196K	+	+++
N200A	+++	+++
R204D	++	++++
E262G*	-	++
E262K	+	+++
G266E	++++	++++
S269F	+++++	+++
V273E	++++	++++
S284D	+++	++++

- less than 40% of wild-type interaction; + 40-59% of wild-type interaction; ++ 60-89% of wild-type interaction; +++ 90-100% of wild-type interaction; ++++ 101-200% of wild-type interaction; +++++ greater than 200% of wild-type interaction

[✚]λCI-CbtA interactions were assessed at 100 µM IPTG

[❖]λCI-RodZ$_{NTD}$ interactions were assessed at 25 µM IPTG

* These mutants were identified in our original two-hybrid screen
