# Supplementary material for: CbtA toxin of Escherichia coli inhibits cell division and cell elongation via direct and independent interactions with FtsZ and MreB
Source: PLoS Genet. 2017 Sep 20;13(9):e1007007. doi: 10.1371/journal.pgen.1007007 (PMC5624674; doi:10.1371/journal.pgen.1007007)
Supplement: S2 Table — (PDF) [file pgen.1007007.s008.pdf]

S2 Table. Bacterial strains used in this study.

Strain	Description	Reference/Source
***E. coli* strains:**		
NEB5-α F'I^{q}	DH5-α derivative containing an F' (Tet resistant) bearing *lacI^{q}*	New England Biolabs
BW27785	Δ*(araB-araD)567* Δ*lacZ4787*(::rrnB-3) LAM- Δ*(araH-araF)570*(::FRT) ΔaraEp-532::FRT φ(*Pcp18-araE534*) Δ(*rhaB-rhaD)568 hsdR514*	[1,2]
NP1 (TB28 *zapA-gfp frt*)	TB28 (MG1655 Δ*lacIZYA*::*frt*) producing ZapA-GFP from the native chromosomal locus. Linked *cat* cassette has been removed by FLP recombinase.	[3]
TB28 HKHC488	TB28 with plasmid pCH488 (*plac:sfgfp-ftsZ bla*) integrated into the chromosome at the attHK002 site.	H. Cho and T. Bernhardt
FW102 O_L2-62	FW102 [4] containing an F' (Kan resistant) bearing the *plac*O_L2–62-*lacZ* fusion in which the λCI operator is centered at position –62 upstream of the *lac* promoter.	[5]
BN30 (FW102 O_L2-42)	FW102 [4] containing an F' (Kan resistant) bearing the *plac*OL_2–42-*lacZ* fusion in which the λCI operator is centered at position –42 upstream of the *lac* promoter.	[6]
CH45/pDB346	*ftsZ^{0}/λPR-ftsZ, cI857; ftsZ* expression is repressed upon shift to 30 ºC ; plasmid confers resistance to Spec	[7,8]
FB30/pFB174	TB28 (MG1655 Δ*lacIZYA*::*frt*) *ΔmreBCD::kan* transformed with Cm resistant plasmid pFB174 (*pBAD-mreBCD-LE)*	[9]
DH73	BW27785 with *ftsZ-L169P* allele at the endogenous *ftsZ* locus. Mutation is not linked to a selectable marker	This study
DH118/pFB149	BW27785 *ΔmreBCD::kan* transformed with Carb resistant plasmid pFB149 (*plac-mreBCD-LE)*	Strain: this study<br>Plasmid: [9]
DH118/pDH278	BW27785 *ΔmreBCD::kan* transformed with Carb resistant plasmid pDH278 (*plac-mreB-E262G mreCD-LE)*	This study
DH118/pDH332	BW27785 *ΔmreBCD::kan* transformed with Carb resistant plasmid pDH332 (*plac-mreB-S269F mreCD-LE)*	This study

S2 Table (continued). Bacterial strains used in this study.

Strain	Description	Reference/Source
***B. subtilis* strains:**	All PY79 derivatives	[10]
DH84	*ycgO:: pHYPERSPANK-his$_6$-gfp erm*	This study
DH85	*ycgO:: pHYPERSPANK-his$_6$-cbtA-gfp erm*	This study
DH98	*ftsZ spec (spec* cassette is immediately downstream of the wildtype *ftsZ* ORF)	This study
DH99	*ftsZ (loop^{Eco}) spec (spec* cassette is immediately downstream of the *ftsZ* locus. The wt *ftsZ* allele is replaced with a chimeric *ftsZ* encoding *Eco* residues 168-182 in place of *Bsu* residues 169-183 in the context of full-length *Bsu ftsZ.*)	This study
DH100	*ftsZ spec, ycgO:: pHYPERSPANK-his$_6$-gfp erm*	This study
DH101	*ftsZ spec, ycgO:: pHYPERSPANK-his$_6$-cbtA-gfp erm*	This study
DH102	*ftsZ (loop^{Eco}) spec, ycgO:: pHYPERSPANK-his$_6$-gfp erm*	This study
DH103	*ftsZ (loop^{Eco}) spec, ycgO:: pHYPERSPANK-his$_6$-cbtA-gfp erm*	This study
DH104	*ftsZ spec, ycgO:: pHYPERSPANK-his$_6$-cbtA-F65S-gfp erm*	This study
DH106	*ftsZ (loop^{Eco}) spec, ycgO:: pHYPERSPANK-his$_6$-cbtA-F65S-gfp erm*	This study

1. Khlebnikov A, Datsenko KA, Skaug T. Homogeneous expression of the PBAD promoter in *Escherichia coli* by constitutive expression of the low-affinity high-capacity AraE transporter. Microbiology. 2001;(147):3241–7.

2. Garrity SJ, Sivanathan V, Dong J, Lindquist S, Hochschild A. Conversion of a yeast prion protein to an infectious form in bacteria. Proc Natl Acad Sci U S A. National Acad Sciences; 2010 Jun 8;107(23):10596–601.

3. Peters NT, Dinh T, Bernhardt TG. A Fail-Safe Mechanism in the Septal Ring Assembly Pathway Generated by the Sequential Recruitment of Cell Separation Amidases and Their Activators. J Bacteriol. 2011 Aug 25;193(18):4973–83.

4. Whipple FW. Genetic analysis of prokaryotic and eukaryotic DNA-binding proteins in *Escherichia*

coli. Nucleic Acids Res. Oxford University Press; 1998 Aug 15;26(16):3700–6.

5. Deaconescu AM, Chambers AL, Smith AJ, Nickels BE, Hochschild A, Savery NJ, et al. Structural Basis for Bacterial Transcription-Coupled DNA Repair. Cell. 2006 Feb;124(3):507–20.

6. Castang S, Dove SL. High-order oligomerization is required for the function of the H-NS family member MvaT in *Pseudomonas aeruginosa*. Mol Microbiol. 2010 Sep 24;78(4):916–31.

7. Raskin DM, de Boer PA. The MinE ring: an FtsZ-independent cell structure required for selection of the correct division site in E. coli. Cell. 1997 Nov 28;91(5):685–94.

8. Skoog K, Daley DO. The *Escherichia coli* Cell Division Protein ZipA Forms Homodimers Prior to Association with FtsZ. Biochemistry. 2012 Feb 21;51(7):1407–15.

9. Bendezu FO, de Boer PAJ. Conditional Lethality, Division Defects, Membrane Involution, and Endocytosis in *mre* and *mrd* Shape Mutants of *Escherichia coli*. J Bacteriol. 2008 Feb 20;190(5):1792–811.

10. Youngman PJ, Perkins JB, Losick R. Genetic transposition and insertional mutagenesis in *Bacillus subtilis* with *Streptococcus faecalis* transposon Tn917. Proc Natl Acad Sci U S A. National Academy of Sciences; 1983 Apr;80(8):2305–9.
