# Supplementary material for: CbtA toxin of Escherichia coli inhibits cell division and cell elongation via direct and independent interactions with FtsZ and MreB
Source: PLoS Genet. 2017 Sep 20;13(9):e1007007. doi: 10.1371/journal.pgen.1007007 (PMC5624674; doi:10.1371/journal.pgen.1007007)
Supplement: S3 Table — (PDF) [file pgen.1007007.s009.pdf]

S3 Table. Plasmids used in this study.

Plasmid	Description	Reference/Source
Bacterial two-hybrid constructs:		
pBRα	Encodes the full-length α subunit of RNAP under the control of tandem p*lacUV5* and p*lpp* promoters; confers resistance to Carb	[1]
pACλCI	Encodes λCI (residues 1-236) under the control of *lacUV5* promoter; confers resistance to Cm	[1]
pBRα -βflap (831–1057)	Encodes residues 1-248 of α fused by a three-alanine linker residues 831–1057 of the β subunit of *E. coli* RNAP under the control of tandem p*lacUV5* and p*lpp* promoters; confers resistance to Carb; used to generate NotI/BamHI-digested backbone to construct various α fusions.	[2]
pACλCI-βflap (831–1057)	Encodes λCI (residues 1-236) fused via three alanines to residues 831–1057 of the β subunit of *E. coli* RNAP under the control of *lacUV5* promoter; confers resistance to Cm; used to generate NotI/BamHI-digested backbone to construct various λCI fusions.	[2]
pBRα-FtsZ (pMT153)	Encodes residues 1-248 of α fused by a three-alanine linker to full-length *E. coli* FtsZ under the control of tandem p*lacUV5* and p*lpp* promoters; confers resistance to Carb	This study
pBRα-FtsZΔ66 (pDH198)	pBRα-FtsZ encoding *E. coli* FtsZ residues 2-317; the last 66 residues are deleted	This study
pBRα-FtsZ mutants isolated in two-hybrid screen:<br>pBRα-FtsZ-L169P<br>pBRα-FtsZ-L179R<br>pBRα-FtsZ-S177P<br>pBRα-FtsZ-D180N<br>pBRα-FtsZ-F182S	pBRα-FtsZ encoding the indicated substitution in the FtsZ moiety of the fusion	This study
pBRα-FtsZ-D180K (pDH160)	pBRα-FtsZ encoding the D180K substitution in the FtsZ moiety of the fusion	This study
pACλCI-FtsZ (pMT152)	Encodes λCI fused by a three-alanine linker to full-length *E. coli* FtsZ under the control of *lacUV5* promoter; confers resistance to Cm	This study
pACλCI-*Bsu* FtsZ (pDH62)	Encodes λCI fused by a three-alanine linker to full-length *B. subtilis* FtsZ under the control of *lacUV5* promoter; confers resistance to Cm	This study
pACλCI-*Bsu* FtsZ (loop^{Eco}) (pDH69)	pACλCI-*Bsu* FtsZ with residues 169-183 (ILEIVDKNTPMLEAF) replaced with *Eco* FtsZ residues 168-182 (LLKVLGRGISLDAF) in the *Bsu* FtsZ moiety of the fusion.	This study

pBRα-MreB (pMT151)	Encodes residues 1-248 of α fused by a three-alanine linker to *E. coli* MreB under the control of tandem p*lacUV5* and p*lpp* promoters; confers resistance to Carb	This study
pBRα-MreB mutants isolated in two-hybrid screen:<br>pBRα-MreB-I126V (pDH257)<br>pBRα-MreB-V173A (pDH256)<br>pBRα-MreB-E196G (pDH259)<br>pBRα-MreB-E262G (pDH258)	pBRα-MreB encoding the indicated substitution in the MreB moiety of the fusion	This study
Additional pBRα-MreB mutants:<br>pBRα-MreB-K77D (pDH301)<br>pBRα-MreB-D78K (pDH302)<br>pBRα-MreB-F84A (pDH300)<br>pBRα-MreB-V121E (pDH293)<br>pBRα-MreB-R124D (pDH298)<br>pBRα-MreB-A125D (pDH299)<br>pBRα-MreB-R127D (pDH294)<br>pBRα-MreB-E128K (pDH295)<br>pBRα-MreB-R188D (pDH307)<br>pBRα-MreB-D192K (pDH303)<br>pBRα-MreB-E196K (pDH271)<br>pBRα-MreB-N200A (pDH309)<br>pBRα-MreB-R204D (pDH306)<br>pBRα-MreB-E262K (pDH272)<br>pBRα-MreB-G266E (pDH308)<br>pBRα-MreB-S269F (pDH305)<br>pBRα-MreB-V273E (pDH310)<br>pBRα-MreB-S284D (pDH316)	pBRα-MreB encoding the indicated substitution in the MreB moiety of the fusion	This study
pACλCI-MreB (pMT150)	Encodes λCI fused by a three-alanine linker to full-length *E. coli* MreB under the control of *lacUV5* promoter; confers resistance to Cm	This study
pBRα-CbtA (pMT155)	Encodes residues 1-248 of α fused by a three alanine linker to CbtA under the control of tandem p*lacUV5* and p*lpp* promoters; confers resistance to Carb	This study
pBRα-YpjF (pMT174)	Encodes residues 1-248 of α fused by a three alanine linker to YpjF under the control of tandem p*lacUV5* and p*lpp* promoters; confers resistance to Carb	This study
pBRα-YkfI (pMT173)	Encodes residues 1-248 of α fused by a three alanine linker to YkfI under the control of tandem p*lacUV5* and p*lpp* promoters; confers resistance to Carb	This study
pACλCI-CbtA (pMT154)	Encodes λCI fused by a three-alanine linker to CbtA under the control of *lacUV5* promoter; confers resistance to Cm	This study
pACλCI-CbtA-F65S (pMT180)	pACλCI-CbtA encoding the F65S substitution in the CbtA moiety of the fusion	This study

pACλCI-CbtA-R15C (pDH246)	pACλCI-CbtA encoding the R15C substitution in the CbtA moiety of the fusion	This study
pACλCI-CbtA-V48E (pDH200)	pACλCI-CbtA encoding the V48E substitution in the CbtA moiety of the fusion	This study
pACλCI-YpjF (pMT170)	Encodes λCI fused by a three-alanine linker to YpjF under the control of *lacUV5* promoter; confers resistance to Cm	This study
pACλCI-YpjF-F65S (pMT192)	pACλCI-YpjF encoding the F65S substitution in the YpjF moiety of the fusion	This study
pACλCI-YpjF-C15R (pDH267)	pACλCI-YpjF encoding the C15R substitution in the YpjF moiety of the fusion	This study
pACλCI-YkfI (pMT169)	Encodes λCI fused by a three-alanine linker to YkfI under the control of *lacUV5* promoter; confers resistance to Cm	This study
pACλCI-YkfI-F65S (pDH206)	pACλCI-YkfI encoding the F65S substitution in the YkfI moiety of the fusion	This study
pACλCI-YkfI-C15R (pDH266)	pACλCI-YkfI encoding the C15R substitution in the YkfI moiety of the fusion	This study
pACλCI-ZipA$_{CTD}$ (pDH159)	Encodes λCI fused by a three-alanine linker to residues 186-328 of ZipA under the control of *lacUV5* promoter; confers resistance to Cm	This study
pACλCI-RodZ$_{NTD}$ (pDH238)	Encodes λCI fused by a three-alanine linker to the NTD of RodZ (residues 2-84) under the control of *lacUV5* promoter; confers resistance to Cm	This study
Toxin expression constructs:		
p3-37	Derivative of pCA24N; Encodes full-length His$_6$-YkfI-GFP under the control of *pT5-lac* promoter; pBR origin; confers resistance to Cm	[3]
pMT136	Derivative of pCA24N; Empty vector control plasmid encoding full-length His$_6$-GFP under the control of *pT5-lac* promoter; pBR origin; confers resistance to Cm	This study
pMT138	Derivative of pCA24N; Encodes full-length His$_6$-YpjF-GFP under the control of *pT5-lac* promoter; pBR origin; confers resistance to Cm	This study
pMT139	Derivative of pCA24N; Encodes full-length His$_6$-CbtA-GFP under the control of *pT5-lac* promoter; pBR origin; confers resistance to Cm	This study
pMT144	Derivative of pCA24N; Encodes full-length His$_6$-YkfI-F65S-GFP under the control of *pT5-lac* promoter; pBR origin; confers resistance to Cm	This study

pMT146	Derivative of pCA24N; Encodes full-length His_6-CbtA-F65S-GFP under the control of *pT5-lac* promoter; pBR origin; confers resistance to Cm	This study
pMT188	Derivative of pCA24N; Encodes full-length His_6-YpjF-F65S-GFP under the control of *pT5-lac* promoter; pBR origin; confers resistance to Cm	This study
pDH253	Derivative of pCA24N; Encodes full-length His_6-CbtA-R15C-GFP under the control of *pT5-lac* promoter; pBR origin; confers resistance to Cm	This study
pDH262	Derivative of pCA24N; Encodes full-length His_6-CbtA-R15C/F65S-GFP under the control of *pT5-lac* promoter; pBR origin; confers resistance to Cm	This study
pBAD33	*araC*; pACYC 184 origin; *pBAD* promoter with no insert; confers resistance to Cm	[4]
pDH212	*araC*; pACYC 184 origin; *pBAD-cbtA-F65S*; confers resistance to Cm	This study
pDH289	*araC*; pACYC 184 origin; *pBAD-ypjF-F65S*; confers resistance to Cm	This study
pSG360	Empty vector used for cloning genes downstream of a p*lacUV5* promoter with an additional *lacO* site; pCDF origin; confers resistance to Spec/Strep; *lacI^q*	S. Goldman
pDH325	pSG360 derivative encoding untagged CbtA*;* confers resistance to Spec/Strep	This study
pDH326	pSG360 derivative encoding untagged CbtA-F65S*;* confers resistance to Spec/Strep	This study
pDH327	pSG360 derivative encoding untagged CbtA-R15C*;* confers resistance to Spec/Strep	This study
pDH328	pSG360 derivative encoding untagged CbtA-R15C/F65S*;* confers resistance to Spec/Strep	This study
pSG369	Empty vector used for cloning genes downstream of a p*tet* promoter; pCDF origin; confers resistance to Spec/Strep; *tetR*	S. Goldman
pDH335	pSG369 derivative encoding untagged CbtA-F65S; confers resistance to Spec/Strep	This study
pDH337	pSG369 derivative encoding untagged CbtA-R15C/F65S; confers resistance to Spec/Strep	This study
Other constructs:		
pCX41	Bears a temperature-sensitive origin of replication and a HindIII/ClaI fragment containing wild-type *ftsZ* and flanking homology to *ftsA* and *lpxC*; confers resistance to Cm (10 μg/mL)	[5]; T. Bernhardt
pCX41-F268C	pCX41 derivative bearing *ftsZ-F268C*	T. Bernhardt

pCX41-D180N (pDH33)	pCX41 derivative bearing *ftsZ-D180N*	This study
pCX41-S177P (pDH34)	pCX41 derivative bearing *ftsZ-S177P*	This study
pCX41-L169P (pDH35)	pCX41 derivative bearing *ftsZ-L169P*	This study
pDR3	Encodes full-length *E. coli* FtsZ under the control of *lac* promoter; ColE1 origin with *lacI^q*; confers resistance to Carb	[6,7]; T. Bernhardt
pDR3-D180N (pDH27)	pDR3 derivative encoding FtsZ-D180N	This study
pDR3-S177P (pDH28)	pDR3 derivative encoding FtsZ-S177P	This study
pDR3-F182S (pDH29)	pDR3 derivative encoding FtsZ-F182S	This study
pDR3-L169P (pDH30)	pDR3 derivative encoding FtsZ-L169P	This study
pDH84	Encodes His_6-GFP under the control of *pHYPERSPANK;* confers resistance to Carb in *E. coli* and to MLS in *B. subtilis*	This study
pDH85	Encodes His_6-CbtA-GFP under the control of *pHYPERSPANK;* confers resistance to Carb in *E. coli* and to MLS in *B. subtilis*	This study
pDH102	Encodes His_6-CbtA-F65S-GFP under the control of *pHYPERSPANK;* confers resistance to Carb in *E. coli* and to MLS in *B. subtilis*	This study
pDR111	Used for integration of sequences into *B. subtilis amyE* locus; confers resistance to Carb in *E. coli* and Spec in *B. subtilis*	D. Rudner
pMLB1113	Used as empty vector control for *mreB* complementation; ColE1 origin; Encodes LacZ under the control of the *lac* promoter; *lacI^q*; confers resistance to Carb	[8]; T. Bernhardt
pFB149	ColE1 origin; Encodes wild-type MreBCD under the control of the *lac* promoter. Residues LE are appended to the end of the MreD sequence; *lacI^q*; confers resistance to Carb	[6]; T. Bernhardt
pDH278	pFB149 derivative encoding MreB-E262G and wild-type MreCD	This study
pDH332	pFB149 derivative encoding MreB-S269F and wild-type MreCD	This study

1. Dove SL, Joung JK, Hochschild A. Activation of prokaryotic transcription through arbitrary protein-protein contacts. Nature. 1997 Apr 10;386(6625):627–30.

2. Deighan P, Diez CM, Leibman M, Hochschild A, Nickels BE. The bacteriophage λ Q antiterminator protein contacts the β-flap domain of RNA polymerase. Proc Natl Acad Sci U S A. National Acad Sciences; 2008 Oct 7;105(40):15305–10.

3. Kitagawa M, Ara T, Arifuzzaman M, Ioka-Nakamichi T, Inamoto E, Toyonaga H, et al. Complete set of ORF clones of *Escherichia coli* ASKA library (A Complete Set of *E. coli* K-12 ORF Archive): Unique Resources for Biological Research. DNA Res. 2006 Jan 9;12(5):291–9.

4. Guzman LM, Belin D, Carson MJ, Beckwith J. Tight regulation, modulation, and high-level expression by vectors containing the arabinose P_{BAD} promoter. J Bacteriol. American Society for

Microbiology (ASM); 1995 Jul;177(14):4121–30.

5. Wang XD, de Boer PA, Rothfield LI. A factor that positively regulates cell division by activating transcription of the major cluster of essential cell division genes of *Escherichia coli*. EMBO J. 1991 Nov;10(11):3363–72.

6. Bendezu FO, de Boer PAJ. Conditional Lethality, Division Defects, Membrane Involution, and Endocytosis in *mre* and *mrd* Shape Mutants of *Escherichia coli*. J Bacteriol. 2008 Feb 20;190(5):1792–811.

7. Bernhardt TG, de Boer PAJ. SlmA, a Nucleoid-Associated, FtsZ Binding Protein Required for Blocking Septal Ring Assembly over Chromosomes in *E. coli*. Mol Cell. 2005 May;18(5):555–64.

8. de Boer PA, Crossley RE, Rothfield LI. A division inhibitor and a topological specificity factor coded for by the minicell locus determine proper placement of the division septum in *E. coli*. Cell. 1989 Feb 24;56(4):641–9.
