# Supplementary material for: CbtA toxin of Escherichia coli inhibits cell division and cell elongation via direct and independent interactions with FtsZ and MreB
Source: PLoS Genet. 2017 Sep 20;13(9):e1007007. doi: 10.1371/journal.pgen.1007007 (PMC5624674; doi:10.1371/journal.pgen.1007007)
Supplement: S4 Table — (PDF) [file pgen.1007007.s010.pdf]

S4 Table. Oligonucleotides used in this study.

Name	Sequence (5' to 3')	Description
oDH30	TCCCTGCTGAATGCGTTTGGCGCAGCG	For overlap PCR to generate *ftsZ-D180N*
oDH31	CGCTGCGCCAAACGCATTCAGCAGGG	For overlap PCR to generate *ftsZ-D180N*
oDH32	GCGGTATCCCCCTGCTGGATGCGTTTGG	For overlap PCR to generate *ftsZ-S177P*
oDH33	CCAAACGCATCCAGCAGGGGGATACCG	For overlap PCR to generate *ftsZ-S177P*
oDH34	GACAAACTGCCGAAAGTTCTGGGCCGCGG	For overlap PCR to generate *ftsZ-L169P*
oDH35	CCGCGGCCCAGAACTTTCGGCAGTTTG	For overlap PCR to generate *ftsZ-L169P*
oDH36	GCATTAAAGCTTCGCCAACAAGGGGTTAAACATCAC CTGG	Forward primer to clone *ftsZ* alleles into pCX41; contains HindIII site
oDH37	GCTTATCATCGATGCCCAAGCCCGCGAGA GCAG	Reverse primer to clone *ftsZ* alleles into pCX41; contains ClaI site
oDH70	TATATAGCGGCCGCATTTGAACCAATGGAAC	Forward primer for amplification of *ftsZ;* contains a NotI site
oDH71	TATATAGGATCCTTAATCAGCTTGCTTACGC	Reverse primer for amplification of *ftsZ*; contains BamHI site
oDH86	TATATAGCGGCCGCATTGGAGTTCGAAACAAAC ATAG	Forward primer for amplification of *Bsu ftsZ;* contains a NotI site
oDH87	TATATAGGATCCTTAGCCGCGTTTATTACGG	Reverse primer for amplification of *Bsu ftsZ*; contains BamHI site
oDH101	CAGGGAGATACCGCGGCCCAGAACTTTCAGCAGAC GGTCGTTCGGGATCACGATC	Reverse primer for overlap PCR to generate *Bsu ftsZ (loop^{Eco})*
oDH102	TGGGCCGCGGTATCTCCCTGCTGGATGCGTTTCGC GAAGCGGATAACGTAC	Forward primer for overlap PCR to generate *Bsu ftsZ (loop^{Eco})*
oDH108	ATATATATGCTAGCTTATTTGTATAGTTCATCCATGC CATGTGTAATC	Reverse primer to clone *cbtA* alleles into *B. subtilis* integration constructs; contains NheI site
oDH116	ATATATAAGCTTACATAAGGAGGAACTACTATGAGAG GATCTCACCATCACCATC	Forward primer to clone *cbtA* alleles into *B. subtilis* integration constructs; contains HindIII site
oDH124	ATATATGTCGACGTCTTAGGAGGATTTAGCATGTTG GAGTTCGAAACAAACATAG	*B. subtilis ftsZ* sequencing primer
oDH125	ATATATGGATCCTTAGCCGCGTTTATTACGGTTTC	*B. subtilis ftsZ* sequencing primer
oDH127	TGACATGGTATTCGTGACAGC	*B. subtilis ftsZ* sequencing primer
oDH130	CTCCGCTGCGTTGAAAGAG	For construction of strain DH98
oDH131	GGGCTGCAGGAATTCTTAGCCGCGTTTATTACGGTT TC	For construction of strain DH98
oDH132	TAATAAACGCGGCTAAGAATTCCTGCAGCCCTGGCG	For construction of strain DH98
oDH133	ATTTTGTCCTTTACAGATCCCCCTATGCAAGGGTT	For construction of strain DH98
oDH134	TTGCATAGGGGGATCTGTAAAGGACAAAATCGTTTT G	For construction of strain DH98
oDH135	AGATGTCACACTCACATTG	For construction of strain DH98
oDH141	GATTTGCTGAAAAAGCTG	For construction of strain DH99
oDH142	GTTTGTTTCGAACTCCAACATGCTAAATCCTCCTAAT CTGCC	For construction of strain DH99
oDH143	TTAGGAGGATTTAGCATGTTGGAGTTCGAAACAAAC ATAG	For construction of strain DH99
oDH144	GAAGCACCTTTTCCACTTCG	For construction of strain DH99
oDH167	AAGCACATAAAAATATTCTG	Amplifies *B. subtilis ftsA ftsZ*
oDH168	GCCAGTCACGTTACGTTATTAG	Amplifies *B. subtilis ftsA ftsZ*
oDH172	ATAATAAACAGAGCAAAATG	*B. subtilis ftsZ* sequencing primer
oDH173	TTATAACATGTATTCACGAAC	*B. subtilis ftsZ* sequencing primer

oDH237	TATATAGGATCCTCAGGCGTTGGCGTCTTTG	Reverse primer for amplification of *zipA-CTD;* contains a BamHI site
oDH238	TATATAGCGGCCGCAGATAAACCGAAGCGCAAAG	Forward primer for amplification of *zipA-CTD;* contains a NotI site
oDH239	CCGCGGTATCTCCCTGCTGAAAGCGTTTGGCGCAG CGAACG	For overlap PCR to generate *ftsZD180K*
oDH240	CGTTCGCTGCGCCAAACGCTTTCAGCAGGGAGATA CCGCGG	For overlap PCR to generate *ftsZD180K*
oDH279	TATATAGGATCCTTACATGCCGATACCTGTCGC	Reverse primer to clone *ftsZΔ66*; BamHI site
oDH285	ATATATTCTAGATTATTTCGCCTCCGGATACTTAC	Reverse primer used to amplify *cbtA* for pBAD33; contains XbaI site
oDH286	ATATATCATATGAAAACATTACCTGTATTACCCGGG	Forward primer used to amplify *cbtA* for pBAD33; contains NdeI site
oDH289	AATTTCCTGCAGAATACCG	Anneals downstream of chromosomal *mreD*; used for PCR verification of *ΔmreBCD*
oDH307	AAGTAAGCGGATTTTCTTTTCC	Anneals upstream of chromosomal *mreB*; used for PCR verification of *ΔmreBCD*
oDH336	ATATATGCGGCCGCAAATACTGAAGCCACGCACG	Forward primer for amplification of *rodZ-NTD*; contains NotI site
oDH337	ATATATGGATCCTTATTCCAGCCCTGGCAGCAG	Reverse primer for amplification of *rodZ-NTD*; contains BamHI site
oDH344	ATATATGCGGCCGCAAAAACTTTACCTGCAATAACT CAGCGGGCGGTGAAGCCCCGTCTGTCACCCGTGG CTG	Forward mutagenic primer to introduce C15R into *ykfI*; contains NotI site
oDH345	ATATATGCGGCCGCAAACACTCTACCTGCTACAAT TTCGCAGGCGGCGAAGCCCCGCCTGTCGCCAGTG GCTG	Forward mutagenic primer to introduce C15R into *ypjF*; contains NotI site
oDH346	GGTGGTGACCGTTTCGACAAAGCTATCATCAACTAT GTG	*mreB* mutagenic forward primer; E196K
oDH347	CACATAGTTGATGATAGCTTTGTCGAAACGGTCACC ACC	*mreB* mutagenic reverse primer; E196K
oDH348	ATCCTCGAAGCACTGCAGAAACCGCTGACCGGTAT TGTG	*mreB* mutagenic forward primer; E262K
oDH349	CACAATACCGGTCAGCGGTTTCTGCAGTGCTTCGA GGAT	*mreB* mutagenic reverse primer; E262K
oDH355	AAGTTATGCGTATTCTCG	Anneals between *mreB* and *mreC*; used to sequence pFB149
oDH357	GGTGGTACCACTGAAGCTGCTGTTATCTCCTTG	*mreB* mutagenic forward primer; V173A
oDH358	CAAGGAGATAACAGCAGCTTCAGTGGTACCACC	*mreB* mutagenic reverse primer; V173A
oDH359	GTTGAACGCCGCGCAGTTCGTGAATCCGCG	*mreB* mutagenic forward primer; I126V
oDH360	CGCGGATTCACGAACTGCGCGGCGTTCAAC	*mreB* mutagenic reverse primer; I126V
oDH361	GTGGTGACCGTTTCGACGGAGCTATCATCAACTATG	*mreB* mutagenic forward primer; E196G
oDH362	CATAGTTGATGATAGCTCCGTCGAAACGGTCACCAC	*mreB* mutagenic reverse primer; E196G
oDH363	CTCGAAGCACTGCAGGGACCGCTGACCGGTATTG	*mreB* mutagenic forward primer; E262G
oDH364	CAATACCGGTCAGCGGTCCCTGCAGTGCTTCGAG	*mreB* mutagenic reverse primer; E262G
oDH369	GCGTCGAGCCGCTGATCAG	Anneals within *mreD* sequence downstream of BamHI site
oDH372	ATATATTCTAGACAGCTTTCAGGATTATCCCTTAGTA	Forward primer to amplify *mreB*

	TGTTGAAAAAATTTCGTG	sequence for pFB149 derivatives; contains XbaI site
oDH373	CAGGAAACAGCTATGACCATG	Sequencing primer for pFB149 derivatives; anneals upstream of *mreB* and XbaI site
oDH374	TGTGCTGCAAGGCGATTAAG	Sequencing primer for pFB149 derivatives; anneals downstream of *mreD*
oDH375	CGGCGGCGACCTGTTCAG	Sequencing primer for pFB149 derivatives; anneals upstream of *mreC*
oDH380	ATATATCATATGAACACTCTACCTGCTACAATTTCG	Forward primer used to amplify *ypjF* for pBAD33; contains NdeI site
oDH381	ATATATTCTAGATTATTTCACATTAGTTTTTAG	Reverse primer used to amplify *cbtA* for pBAD33; contains XbaI site
oDH388	GCCGGTTGGCGCGACCCAGGAAGAACGCCGCGCAATTCG	*mreB* mutagenic forward primer; V121E
oDH389	CGAATTGCGCGGCGTTCTTCCTGGGTCGCGCCAACCGGC	*mreB* mutagenic reverse primer; V121E
oDH390	CCCAGGTTGAACGCCGCGCAGACCGTGAATCCGCGCAGGG	*mreB* mutagenic forward primer; R127D
oDH391	CCCTGCGCGGATTCACGGTCTGCGCGGCGTTCAACCTGGG	*mreB* mutagenic reverse primer; R127D
oDH392	GAACGCCGCGCAATTCGTAAATCCGCGCAGGGCGCTGG	*mreB* mutagenic forward primer; E128K
oDH393	CCAGCGCCCTGCGCGGATTTACGAATTGCGCGGCGTTC	*mreB* mutagenic reverse primer; E128K
oDH399	CGACCCAGGTTGAACGCGACGCAATTCGTGAATCCGCG	*mreB* mutagenic forward primer; R124D
oDH400	CGCGGATTCACGAATTGCGTCGCGTTCAACCTGGGTCG	*mreB* mutagenic reverse primer; R124D
oDH401	GACCCAGGTTGAACGCCGCGACATTCGTGAATCCGCGCAG	*mreB* mutagenic forward primer; A125D
oDH402	CTGCGCGGATTCACGAATGTCGCGGCGTTCAACCTGGGTC	*mreB* mutagenic reverse primer; A125D
oDH403	GACGGCGTTATCGCCGACGCCTTCGTGACTGAAAAAATG	*mreB* mutagenic forward primer; F84A
oDH404	CATTTTTTCAGTCACGAAGGCGTCGGCGATAACGCCGTC	*mreB* mutagenic reverse primer; F84A
oDH405	GCTGCCATTCGCCCAATGGACGACGGCGTTATCGCCGAC	*mreB* mutagenic forward primer; K77D
oDH406	GTCGGCGATAACGCCGTCGTCCATTGGGCGAATGGCAGC	*mreB* mutagenic reverse primer; K77D
oDH407	GCCATTCGCCCAATGAAAAAAGGCGTTATCGCCGACTTC	*mreB* mutagenic forward primer; D78K
oDH408	GAAGTCGGCGATAACGCCTTTTTTCATTGGGCGAATGGC	*mreB* mutagenic reverse primer; D78K
oDH409	CTGTGCGCATTGGTGGTAAACGTTTCGACGAAGCTATC	*mreB* mutagenic forward primer; D192K
oDH410	GATAGCTTCGTCGAAACGTTTACCACCAATGCGCACAG	*mreB* mutagenic reverse primer; D192K
oDH413	CGCTGACCGGTATTGTGTTCGCGGTAATGGTTGCACTG	*mreB* mutagenic forward primer; S269F
oDH414	CAGTGCAACCATTACCGCGAACACAATACCGGTCAGCG	*mreB* mutagenic reverse primer; S269F
oDH415	CATCAACTATGTGCGTGATAATTACGGTTCTCTGATCG	*mreB* mutagenic forward primer; R204D
oDH416	CGATCAGAGAACCGTAATTATCACGCACATAGTTGATG	*mreB* mutagenic reverse primer; R204D

oDH417	GTTTACTCCTCTTCTGTGGACATTGGTGGTGACCGT TTCG	*mreB* mutagenic forward primer; R188D
oDH418	CGAAACGGTCACCACCAATGTCCACAGAAGAGGAG TAAAC	*mreB* mutagenic reverse primer; R188D
oDH419	CTGCAGGAACCGCTGACCGAAATTGTGAGCGCGGT AATG	*mreB* mutagenic forward primer; G266E
oDH420	CATTACCGCGCTCACAATTTCGGTCAGCGGTTCCTG CAG	*mreB* mutagenic reverse primer; G266E
oDH421	GTTTCGACGAAGCTATCATCGCATATGTGCGTCGTA ATTACG	*mreB* mutagenic forward primer; N200A
oDH422	CGTAATTACGACGCACATATGCGATGATAGCTTCGT CGAAAC	*mreB* mutagenic reverse primer; N200A
oDH423	GTATTGTGAGCGCGGTAATGGAAGCACTGGAACAG TGCCCG	*mreB* mutagenic forward primer; V273E
oDH424	CGGGCACTGTTCCAGTGCTTCCATTACCGCGCTCA CAATAC	*mreB* mutagenic reverse primer; V273E
oDH434	GTGCCCGCCGGAACTGGCTGACGACATCTCCGAGC GCGG	*mreB* mutagenic forward primer; S284D
oDH435	CCGCGCTCGGAGATGTCGTCAGCCAGTTCCGGCGG GCAC	*mreB* mutagenic reverse primer; S284D
oDH438	CCGGCATTCCAGTCGTTGTTGCTAAAGACCCGCTG ACCTGTGTGG	*mreB* mutagenic forward primer; E319K
oDH439	CCACACAGGTCAGCGGGTCTTTAGCAACAACGACT GGAATGCCGG	*mreB* mutagenic reverse primer; E319K
oDH446	ATATATGAATTCAAGGAGATATACCATGAAAACATT ACCTGTATTACCCG	Forward primer to clone *cbtA* variants into pSG360; contains EcoRI site; includes RBS
oDH447	ATATATAAGCTTTCATTTCGCCTCCGGATACTTACC CAG	Reverse primer to clone *cbtA* variants into pSG360; contains HindIII site
oPDR31	CCCTCTAGAAATAATTTTGTTTAACTTTAAGAAGGAG ATATACATATGTTTGAACCAATGGAACTTAC	For cloning *ftsZ* alleles into pDR3; contains XbaI site
oPDR32	GCCAAGCTTGTCGACTCTTAATCAGCTTGCTTACGC AGG	For cloning *ftsZ* alleles into pDR3; contains HindIII site
oPDR33	GAATTCCCGGGGATCTCGATCCCG	Sequencing primer for pDR3 derivatives
oPDR34	ATGTGCTGCAAGGCGATTAAG	Sequencing primer for pDR3 derivatives
oSG623	GGGCCATCGATGCCGCGGCATCTAGAGGCCTATG	Annealed with oSG624 to form in-frame linker with MCS for pMT136
oSG624	AGGCCTCTAGATGCCGCGGCATCGATGGCCCTCA	Annealed with oSG623 to form in-frame linker with MCS for pMT136
oSG625	AGGAGAAATTAACTATGAGAG	Sequencing primer for pCA24N-derived plasmids
oSG626	AACATCACCATCTAATTCAACAAG	Sequencing primer for pCA24N-derived plasmids
oSG639	GGGGACAAGTTTGTACAAAAAAGCAGGCTTCAACG TTAACACTCTACCTGCTACAATTTC	Amplifies *ypjF* sequence (no start codon); contains AclI site
oSG640	GGGGACCACTTTGTACAAGAAAGCTGGGTCTCTAGA TTTCACATTAGTTTTTAGCAAG	Amplifies *ypjF* sequence (no stop codon); contains XbaI site
oSG641	GGGGACAAGTTTGTACAAAAAAGCAGGCTTCAACG TTAAAACATTACCTGTATTACCCG	Amplifies *cbtA* sequence (no start codon); contains AclI site
oSG642	GGGGACCACTTTGTACAAGAAAGCTGGGTCTCTAG ATTTCGCCTCCGGATACTTAC	Amplifies *cbtA* sequence (no stop codon); contains XbaI site
oSG659	GGGGACAAGTTTGTACAAAAAAGCAGGCTTCAACG TTAAAACTTTACCTGCAATAACTCAGCGG	Amplifies *ykfI* sequence (no start codon); contains AclI site
oSG660	GGGGACCACTTTGTACAAGAAAGCTGGGTCTCTAG ATCGTACTACGTTGTTACGGC	Amplifies *ykfI* sequence (no stop codon); contains XbaI site
oSG663	CCCTAGCCGATGCCGTGAATTCTCTGGTAGAAAAA TACGAGCTGG	Internal mutagenic primer to introduce F65S into *ykfI* sequence

oSG664	CCAGCTCGTATTTTTCTACCAGAGAATTCACGGCA TCGGCTAGGG	Internal mutagenic primer to introduce F65S into *ykfI* sequence
oSG667	TCACTGTGTGATGCGGTGAACTCTCTCGTGGAAAA ATACGCGCTGGTGCG	Internal mutagenic primer to introduce F65S into *cbtA* sequence
oSG668	CGCACCAGCGCGTATTTTTCCACGAGAGAGTTCAC CGCATCACACAGTGA	Internal mutagenic primer to introduce F65S into *cbtA* sequence
oSG723	GGGGACAAGTTTGTACAAAAAAGCAGGCTTCGCG GCCGCATTGAAAAAATTTCGTGGCATGTTTTCC	Forward primer for amplification of *mreB;* contains a NotI site
oSG724	GGGGACCACTTTGTACAAGAAAGCTGGGTCGGAT CCTTACTCTTCGCTGAACAGGTCGCC	Reverse primer for amplification of *mreB*; contains BamHI site
oSG727	GGGGACAAGTTTGTACAAAAAAGCAGGCTTCGCG GCCGCAAAAACATTACCTGTATTACCCGGG	Forward primer for amplification of *cbtA;* contains a NotI site
oSG728	GGGGACCACTTTGTACAAGAAAGCTGGGTCGGAT CCTTATTTCGCCTCCGGATACTTACC	Reverse primer for amplification of *cbtA*; contains BamHI site
oSG729	GGGGACAAGTTTGTACAAAAAAGCAGGCTTCGCG GCCGCAAAAACTTTACCTGCAATAACTCAGCGG	Forward primer for amplification of *ykfI;* contains a NotI site
oSG730	GGGGACCACTTTGTACAAGAAAGCTGGGTCGGAT CCTTATCGTACTACGTTGTTACGGC	Reverse primer for amplification of *ykfI*; contains BamHI site
oSG731	GGGGACAAGTTTGTACAAAAAAGCAGGCTTCGCG GCCGCAAACACTCTACCTGCTACAATTTCGC	Forward primer for amplification of *ypjF;* contains a NotI site
oSG732	GGGGACCACTTTGTACAAGAAAGCTGGGTCGGAT CCTTATTTCACATTAGTTTTTAGCAAGCCGG	Reverse primer for amplification of *ypjF*; contains BamHI site
pBRα_F	GAACAGCGTACCGACCTGGAC	Sequencing primer for all pBRα fusion constructs
pBRα_R	CCTATATCGCCGACATCACC	Sequencing primer for all pBRα fusion constructs
pACλCI_F	GATCAGGGATAGCGGTCAGG	Sequencing primer for all pACλCI fusion constructs
pACλCI_R	CCTACATCTGTATTAACGAAGC	Sequencing primer for all pACλCI fusion constructs
